# Lie Group Statistics and Lie Group Machine Learning Based on Souriau Lie Groups Thermodynamics & Koszul-Souriau-Fisher Metric: New Entropy Definition as Generalized Casimir Invariant Function in Coadjoint Representation

**DOI:** 10.3390/e22060642

**Published:** 2020-06-09

**Authors:** Frédéric Barbaresco

**Affiliations:** Key Technology Domain PCC (Processing, Control & Cognition) Representative, Thales Land & Air Systems, Voie Pierre-Gilles de Gennes, F91470 Limours, France; frederic.barbaresco@thalesgroup.com

**Keywords:** Lie groups thermodynamics, Lie group machine learning, Kirillov representation theory, coadjoint orbits, moment map, covariant Gibbs density, maximum entropy density, Souriau-Fisher metric, generalized Casimir invariant function

## Abstract

In 1969, Jean-Marie Souriau introduced a “Lie Groups Thermodynamics” in Statistical Mechanics in the framework of Geometric Mechanics. This Souriau’s model considers the statistical mechanics of dynamic systems in their “space of evolution” associated to a homogeneous symplectic manifold by a Lagrange 2-form, and defines in case of non null cohomology (non equivariance of the coadjoint action on the moment map with appearance of an additional cocyle) a Gibbs density (of maximum entropy) that is covariant under the action of dynamic groups of physics (e.g., Galileo’s group in classical physics). Souriau Lie Group Thermodynamics was also addressed 30 years after Souriau by R.F. Streater in the framework of Quantum Physics by Information Geometry for some Lie algebras, but only in the case of null cohomology. Souriau method could then be applied on Lie groups to define a covariant maximum entropy density by Kirillov representation theory. We will illustrate this method for homogeneous Siegel domains and more especially for Poincaré unit disk by considering SU(1,1) group coadjoint orbit and by using its Souriau’s moment map. For this case, the coadjoint action on moment map is equivariant. For non-null cohomology, we give the case of Lie group SE(2). Finally, we will propose a new geometric definition of Entropy that could be built as a generalized Casimir invariant function in coadjoint representation, and Massieu characteristic function, dual of Entropy by Legendre transform, as a generalized Casimir invariant function in adjoint representation, where Souriau cocycle is a measure of the lack of equivariance of the moment mapping.

  *La thèse de Kirillov, parue en 1962, a suscité immédiatement beaucoup d’intérêt…En outre, quantité de notions naturelles concernant les représentations s’interprètent géométriquement en terme d’orbites coadjointes: restriction à un sous-groupe, induction unitaire, produit tensoriel, mesure de Plancherel, la topologie de l’ensemble représentations unitaires irréductibles… Kirillov s’est vite convaincu, et il a convaincu la communauté mathématique que cette « méthode des orbites » devait être applicable à des groupes bien plus généraux que les groupes nilpotents. Il n’a pas hésité à aborder le cas des groupes de Lie connexes quelconques. Evidemment, des difficultés considérables ont surgi immédiatement. Néanmoins, Kirillov a indiqué une voie d’accès, qui ensuite a été largement utilisée.*

- **Jacques Dixmier, Brèves remarques sur l’œuvre de A.A. Kirillov**

  *On comprend ainsi comment Lagrange a pu développer les lois de la Mécanique des systèmes formés de solides sans s’occuper des variations de la température de ces corps et Fourier traiter des variations de la température de ces mêmes corps solides sans s’occuper de leur mouvement; comment on peut étudier le mouvement de la Terre, assimilée à un solide rigide, sans se préoccuper de la température de cet astre et étudier le refroidissement du globe terrestre sans se préoccuper de son mouvement. Une telle indépendance entre les problèmes qui ressortissent à la Mécanique et les problèmes qui ressortissent à la Théorie de la chaleur n’existe plus lorsque les systèmes auxquels on a affaire ne sont plus des systèmes classiques; si, par exemple, au lieu de regarder la Terre comme un solide rigide, d’état invariable, on tient compte des changements de volume, de forme, d’état physique et chimique qui accompagnent son refroidissement, on ne peut plus séparer le problème du mouvement de la Terre et le problème du refroidissement terrestre. … On sait que cette forme de relations supplémentaires avait été introduite par Newton et les géomètres du XVIIIème siècle dans la théorie du son. Ces considérations montrent que les questions qui ressortissent à la Thermodynamique ont dû solliciter l’attention des physiciens dès qu’on a voulu aborder l’étude des systèmes autres que des systèmes classiques; et, en fait, c’est la théorie de la propagation du son dans l’air qui a provoqué Laplace à créer la Thermodynamique.*

- **P. Duhem, L’intégrale des forces vives en thermodynamique, JMPA 4:5-19, 1898[1,2,3,4]**

  *Sous cette aspiration, la physique qui était d’abord une science des “agents” doit devenir une science des “milieux”. C’est en s’adressant à des milieux nouveaux que l’on peut espérer pousser la diversification et l’analyse des phénomènes jusqu’à en provoquer la géométrisation fine et complexe, vraiment intrinsèque…Sans doute, la réalité ne nous a pas encore livré tous ses modèles, mais nous savons déjà qu’elle ne peut en posséder un plus grand nombre que celui qui lui est assigné par la théorie mathématique des groupes*

- **Gaston Bachelard, Etude sur l’Evolution d’un problème de Physique –La propagation thermique dans les solides, 1928**

## 1. Introduction

The previous French quotes by the Mathematician Jacques Dixmier, the Physicist Pierre Duhem, and the Philosopher Gaston Bachelard are important to introduce the epistemological context of models that will be developed in the paper. Jacques Diximer refers to Alexander Kirillov seminal idea of coadjoint orbits method to consider Lie group representation model. Pierre Duhem makes comments to the origin of the gap between the theory of heat and the theory of Mechanics. Finally, Gaston Bachelard make prediction that new Thermodynamics foundations will be given by groups. We will try in this paper, to prove that these ideas could be reconciled by the Souriau model of Lie groups Thermodynamics through the mathematical structure of Lie algebra cohomology.

After a the state of the art and trends in Machine Learning based on Information Geometry, we will present, in this introduction, the main objective of this paper to jointly apply models from geometric statistical mechanics and tools from Information geometry to solve “Gauss density” definition problem for statistics on Lie groups and homogeneous manifolds. We will also present use-cases motivation for Lie group machine learning illustrating for Doppler statistics analysis with SU(1,1) statistics, and for kinematics data analysis with SE(2) statistics.

### 1.1. State of the Art and Trends in Machine Learning Based on Information Geometry

The classical simple gradient descent used in Deep Learning has two drawbacks: the use of the same non-adaptive learning rate for all parameter components, and a non-invariance with respect to parameter re-encoding inducing different learning rates. As the parameter space of multilayer networks forms a Riemannian space equipped with Fisher information metric, instead of the usual gradient descent method, the natural gradient or Riemannian gradient method, which takes account of the geometric structure of the Riemannian space, is more effective for learning. The natural gradient preserves this invariance to be insensitive to the characteristic scale of each parameter direction. The Fisher metric defines a Riemannian metric as the Hessian of two dual potential functions (the Entropy and the log-partition function). Yann Ollivier and Gaétan Marceau-Caron provided in 2016 [5] the first experimental results on non-synthetic data sets for the quasi-diagonal Riemannian Natural gradient descents for neural networks introduced previously by Yann Ollivier in [6] (MNIST, SVHN, and FACE data sets). The quasi-diagonal Riemannian algorithms consistently beat simple stochastic gradient gradient descents by a varying margin. The computational overhead with respect to simple backpropagation is around a factor 2, and reach their final performance quickly, thus requiring fewer training epochs and a smaller total computation time. The main goal of natural gradient is to obtain invariance properties, such as, for a neural network, insensitivity of the training algorithm to whether a logistic or tanh activation function is used, or insensitivity to simple changes of variables in the parameters, such as scaling some parameters. In 2017, same authors have introduced the resulting natural Langevin dynamics [7] combining the advantages of natural gradient descent and Fisher-preconditioned Langevin dynamics for large neural networks, validated on MNIST with Fisher matrix preconditioning. With all invariance properties of natural gradient, this Langevin Dynamics avoids overfitting as a regularization method, and replaces classical methods based on a controlled amount of noise to stochastic gradient descents, that ensures convergence to the Bayesian posterior on model parameters. The theoretically optimal covariance of the noise is the inverse Fisher metric, and Y. Ollivier and G. Marceau-Caron have shown how to implement this in practice with neural networks using efficient Fisher metric approximations. In 2017, Yann Ollivier has also introduced TANGO algorithm (True Asymptotic Natural Gradient Optimization) [8], which converges to a true natural gradient descent in the limit of small learning rates, without explicit Fisher matrix estimation, and where in large dimension, small learning rates will be required to approximate the natural gradient well. Y. Ollivier has also shown that it is possible to get arbitrarily close to exact natural gradient descent with a lightweight algorithm. About natural gradient for Deep Learning, we can refer to [9,10]. This year, Shun-ichi Amari [11] has given an elementary geometrical proof that any target function is realized in a sufficiently small neighborhood of any randomly connected deep network, provided the width (the number of neurons in a layer) is sufficiently large.

In this paper, we will introduce how to extend these approaches for data as elements of Lie groups or data lying on a homogeneous manifold where a Lie group acts transitively. This extension is considered in the framework and interconnexion of Souriau “Lie groups Thermodynamics”, Information Geometry and Kirillov representation theory [12] to define probability densities for Lie groups, as Souriau covariant Gibbs densities (density of Maximum of Entropy). We will develop this case for the matrix Lie group SU(1,1) (case with null cohomology) through the computation of Souriau’s moment map, and Kirillov’s orbit method. We will also develop the method for SE(2) Lie group (case with non-null cohomology) where a Souriau cocycle should be taken into account due to the defect of equivariance of the coadjoint action on the moment map.

Supervised learning approaches are based on neural networks whose parameters are estimated by natural gradient algorithms. Non-supervised algorithm are based on clustering by using technics called “k-means” or “Mean-shift” using distance between elements of the dataset. In both cases, if we want to extend these approaches for Lie groups dataset, we have to extend the notion of Gaussian densities and distance between elements. We propose to use Geometric Statistical Model coming from Geometric Statistical Mechanics to introduce “Gauss density” of Lie group elements. Jointly, we can associate a natural distance between these Lie group elements on the Symplectic manifold by means of KKS 2-form, introducing a natural Riemannian metric associated to Fisher Metric from Information Geometry. The objective of this paper is to explain how to use Geometric Statistical Mechanics tools in this context.

### 1.2. Objectives of this Paper

The purpose of this article is multiple. The work of Professor Jean-Marie Souriau is well known in the field of “Geometric Mechanics” of which he is one of the founders with his book “structure of dynamic systems” published in 1969, and in which he introduced the foundations of Symplectic Geometry. Inside this book, chapter IV dealing with the extension of Geometric Mechanics to Statistical Mechanics, has been little read or misunderstood by this community. We have discovered that this model was part of and generalized another discipline, which is called Information Geometry. We have demonstrated in other previous articles that one could generalize Fisher metric (invariant metric used in Information Geometry) for Lie groups, with this model. It is therefore a question of rehabilitating the work of Jean-Marie Souriau in a broader framework, which concerns statistics and machine learning extended to objects considered as elements of a Lie group or a homogeneous manifold.

The second goal is to solve with these new tools problems that were still unsolved in statistics and machine learning. These unresolved problems concern the definition and calculation of the expression of probability densities, playing the role of Gaussian density, for elements of a Lie group or elements of homogeneous manifolds. In this article, we completely solve the problem for 2 Lie groups very useful in machine learning but also in physics, the Lie groups SU(1,1) and SE(2). The calculation is not a simple application of the Souriau model, because it is necessary to establish the “moment map” associated with these groups and define a Laplace transform on their coadjoint orbits of these groups (action of the group on the dual space of Lie algebra). In a second step, we must use Information Geometry to write these covariant Gibbs densities in the correct parametrization which parametrizes the generalized Gaussian law from statistical moments on the homogeneous symplectic manifold associated with coadjoint orbits. In the case SU(1,1), which corresponds to a case of null cohomology (equivariance of the coadjoint operator on the moment application), as the homogeneous symplectic manifold to the coadjoint orbit is the Poincaré unit disk, we solve jointly, an open problem to define mathematically the notion of Gaussian density in this disk in hyperbolic geometry. With the property that this density is by construction invariant under the action of the group SU(1,1), which is the condition sine qua none to preserve the symmetries and the invariance of the associated Fisher metric. We show that this model achieves a breakthrough in machine learning, because we have a Gibbs density and a Fisher metric invariant by change of parametrization and invariant under the action of symmetries. Gibbs density allows us to extend the classical supervised statistical machine learning algorithms, and Fisher metric allows us to adress unsupervised learning problem as k-means problems in metric space. The model opens the way to machine learning for Lie groups with multiple applications in robotics, sensor signal processing, image processing.

In the last part of the article, based on this model, we give a new “geometric” definition of Entropy by showing that Entropy is an invariant Casimir function in coadjoint representation. The Casimir functions have been widely studied within the framework of Poisson structures and manifolds [13,14,15,16]. This characterization of Entropy is new, because previously Entropy was defined axiomatically. Using this Casimir function property, we show that it is possible to use full geometric approaches to construct the Entropy function only from the structure coefficients of the Lie group associated with the symmetries involved. We show that we can also introduce an Euler-Poincaré equation and its stochastic variant to study other open problems in statistics and thermodynamics. The application of this Casimir characterization, which is demonstrated in this article, are developed in another twin article published in the same special issue with François Gay-Balmaz [17].

### 1.3. Motivation of Lie Group Machine Learning with Use-Cases

Machine learning is a field of study of artificial intelligence, which is based on statistical approaches to give computers the ability to “learn” from data, that is, to classify data from observations in a supervised or non-supervised way. Machine learning generally has two phases. The first consists in estimating a model from data, called observations. This so-called “training” phase is generally carried out before the practical use of the model. The second phase corresponds to the start of production: the model being determined, new data can then be classified. According to the information available during the learning phase, learning is qualified in different ways. If the data is labeled (that is, the task response is known for that data), it is supervised learning. We speak of classification if the labels are discrete, or of regression if they are continuous. In the most general case, without a label, we seek to determine the underlying structure of the data (which can be a probability density) and it is then unsupervised learning. Machine learning can be applied to different types of data, such as graphs, trees, curves, or more simply feature vectors, which can be continuous or discrete. We propose to extend the approach, when datasets are element of matrix lie groups.

Learning algorithms can be categorized according to their learning mode. For supervised learning, the classes are predetermined and the examples known, and then the system learns to classify according to a classification model. An expert must label examples beforehand. The process takes place in two phases. For unsupervised learning, when the system or operator has only examples, but no label, and the number of classes and their nature have not been predetermined, we speak of unsupervised learning or clustering. No expert is required. The algorithm must discover for itself the more or less hidden structure of the data, by data partitioning and data clustering. The system must cluster the data according to their available attributes, to classify them into homogeneous groups of examples. Similarity is generally calculated according to a distance function between pairs of examples.

**Figure 1:**
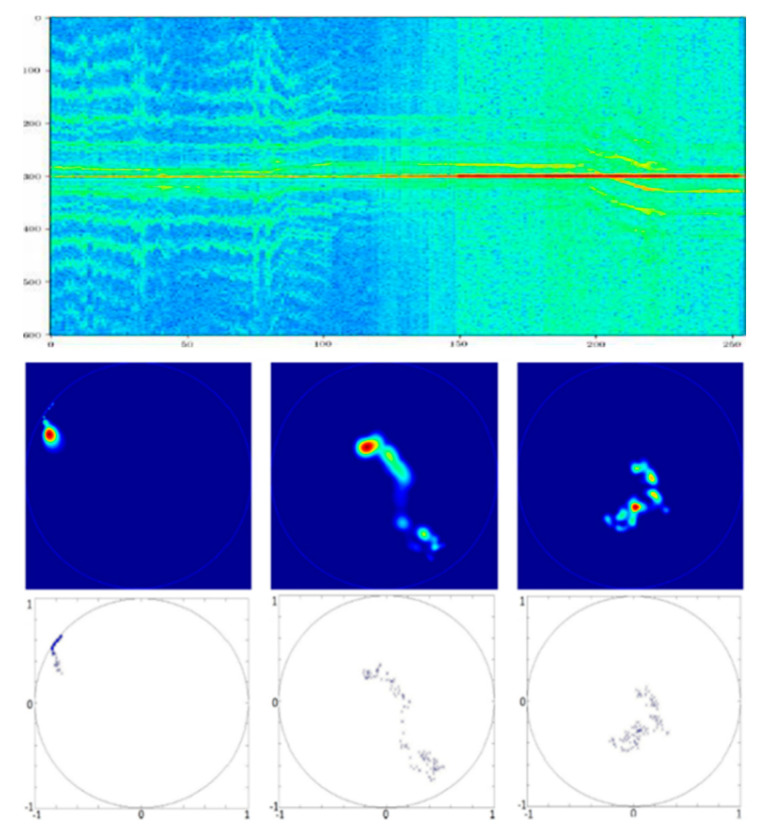
Illustration of Statistics of Doppler Spectrum as densities of points in Poincaré polydisc where SU(1,1) Lie group act transitively. Top is a time-doppler frequency spectrum of a drone. Bottom is a Doppler spectrum fluctuation coded as densities in Poincaré Polydisk.

We will illustrate two problems of Machine Learning on Lie groups coming from Radar Industry. Target recognition on Radar micro-Doppler data could be modeled by a problem of classification of dataset considered as elements of SU(1,1) Lie group (see Figure 1). Radar complex time series of micro-Doppler observation of data are classically processed on sliding time window to estimate their associated covariance matrices that are characterized by a Toeplitz Hermitian Positive-definitiveness structure. Using a well-known Verblunsky/Trench Theorem, we can parametrize all Toeplitz Hermitian Positive Definite Covariance matrices of stationary Radar Time series in a product space with a real positive axis (for signal power) and a Poincaré polydisk (for Doppler Spectrum shape). If we consider the Poincaré Unit Disk as an homogeneous space where SU(1,1) Lie group acts transitively. Each data in Poincaré unit disk of this polydisk could be then coded by SU(1,1) matrix Lie group element. We have transformed the problem into a statistical learning challenge processing data of SU(1,1) matrix Lie group. Another exemple considers flying object recognition on their kinematics coded in SE(2) or SE(3) Lie Groups. 3D (or 2D) trajectories could be coded by SE(3) (or SE(2)) Lie group time series provided through Invariant Extended Kalman Filter (IEKF) Radar Tracker, that locally estimates displacement of Frenet-Seret frame. Object kinematics will be then coded by time series of SE(3) (or SE(2)) matrix Lie groups characterizing local rotation/translation of Frenet frame along the drone 3D (or 2D) trajectory. Statistics of this SE(3) (or SE(2)) Lie group elements will characterize flight mechanics of different kinds of object (birds, drones, …).

SU(1,1) or SE(2) are also fundamental tools in Image Processing (Sub-Riemannian Geometry of vision with SE(2)), in robotics (rigid bodies statistical analysis with SE(2)), in Natural Langage Processing (methods of graph-embedding in Poincaré disk with SU(1,1)), …. For instance, SU(1,1) Lie group which acts on Poincaré unit Disk is highly studied to embed isometrically a graph in an hyperbolic space. It is used by GAFAM (Google, Facebook, …) for Natural Language Processing by reducing graph analysis to a Machine Learning problem in Hyperbolic Poincaré Unit Disk. Hyperbolic Neural network [18] have been developed in this framework. SU(1,1) Lie group is also fundamental in Quantum physics to describe Coherent states of an electron in a magnetic field for instance [19] and Coherent states in Quantum Optics [20] (some statistical photon-counting aspects of SU(1,1) coherent states are emphasized). SE(2) Lie group is especially fundamental for Geometry of Vision considering sub-Riemannian approaches of the Citti-Petitot-Sarti Model [21] but also also in neuroimagery [22].

#### 1.3.1. SU(1,1) Lie Group Machine Learning for Doppler Data Statistics Analysis

Lie group structure appears naturally on Doppler data, if we consider time series of locally stationary signal and their associated covariance matrix. Covariance matrix is Toeplitz Hermitian Positive Definite. Based on Theorem due to Verblunsky [23,24] and Trench [25], we can parametrize Hermitian Positive Definite Matrix in product space involving the Poincaré unit Polydisk:

(1) $$\begin{array}{l} \varphi :THDP(n)\to R_{+}^{*}\times D^{n-1}\\ \;R_{n}\mapsto \left(P_{0},\mu_{1},\ldots ,\mu_{n-1}\right) \end{array}$$

where *D* is the Poincaré Unit Disk:

(2) D={z=x+iy∈C/|z|<1}

The Poincaré unit disk is an homogeneous bounded domain where the Lie group SU(1,1) act transitively. This Matrix Group is given by:

(3) $$SU(1,1)=\left\{\left[\begin{array}{cc} a & b\\ b^{*} & a^{*} \end{array}\right]/{\left|a\right|}^{2}-{\left|b\right|}^{2}=1,\;a,b\in C\right\}$$

where SU(1,1) acts on the Poincaré Unit Disk by:

(4) $$g\in SU(1,1)\Rightarrow g.z=\frac{az+b}{b^{*}z+a}$$

with Cartan Decomposition of SU(1,1)

(5) $$\begin{array}{l} \left(\begin{array}{cc} a & b\\ b^{*} & a^{*} \end{array}\right)=\left|a\right|\left(\begin{array}{cc} 1 & z\\ z^{*} & 1 \end{array}\right)\left(\begin{array}{cc} a/\left|a\right| & 0\\ 0 & a^{*}/\left|a\right| \end{array}\right)\\ \mathrm{with}\;z=b{\left(a^{*}\right)}^{-1},\left|a\right|={\left(1-{\left|z\right|}^{2}\right)}^{-1/2} \end{array}$$

We can observe that $z=b{\left(a^{*}\right)}^{-1}$ could be considered as action of g∈SU(1,1) on the centre on the unit disk $z=g.0=b{\left(a^{*}\right)}^{-1}$. The principal idea is that we can code any point $z=b{\left(a^{*}\right)}^{-1}$ in the unit disk by an element of the Lie group SU(1,1). Main advantage is that the point position is no longer coded by coordinates but intrinsically by transformation from the orogin 0 to this point. Finally, a covariance matrix of a stationary signal could be coded by (*n*−1) Matrix SU(1,1) Lie group elements:

(6) $$\begin{array}{l} \;\mathrm{THPD}\to R_{+}^{*}\times D^{n-1}\;\to R_{+}^{*}\times SU{\left(1,1\right)}^{n-1}\\ R_{n}\mapsto \left(P_{0},\mu_{1},\ldots ,\mu_{n-1}\right)\mapsto \left(P_{0},\left[\begin{array}{cc} a_{1} & b_{1}\\ b_{1}^{*} & a_{1}^{*} \end{array}\right],\ldots ,\left[\begin{array}{cc} a_{n-1} & b_{n-1}\\ b_{n-1}^{*} & a_{n-1}^{*} \end{array}\right]\right) \end{array}$$

#### 1.3.2. SE(2) and SE(3) Lie Groups Machine Learning for Kinematics Data Statistics Analysis

**Figure 2:**
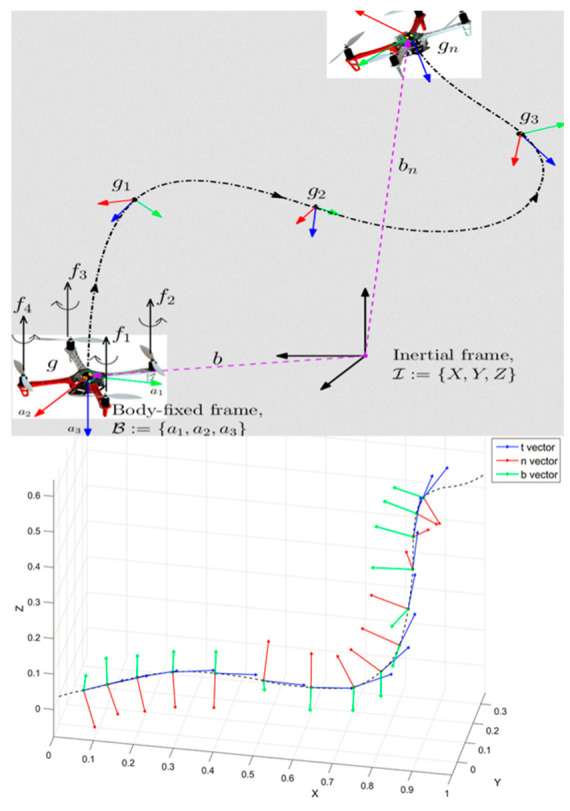
Illustration of times series of local Frenet-Serret Frame where SE(3) Lie group act between two successive frames.

When we consider a 3D trajectory of a mobile target, we can describe this curve by a time evolution of the local Frenet-Serret frame (local frame with tangent vector, normal vector and binormal vector) as illustrated in Figure 2. This frame evolution is described by the Frenet-Serret formula that gives the kinematic properties of the target moving along the continuous, differentiable curve in 3D Euclidean space ℝ^3^. More specifically, the formulas describe the derivatives of the so-called tangent, normal, and binormal unit vectors in terms of each other.

(7) $$\frac{d}{dt}\left(\begin{array}{c} \vec{t}\\ \vec{n}\\ \vec{b} \end{array}\right)=\left[\begin{array}{ccc} 0 & \kappa & 0\\ -\kappa & 0 & \gamma \\ 0 & -\gamma & 0 \end{array}\right]\left(\begin{array}{c} \vec{t}\\ \vec{n}\\ \vec{b} \end{array}\right)\;\mathrm{with}\;\left\{ \begin{array}{l} \kappa :\mathrm{curvature}\\ \gamma :\mathrm{torsion} \end{array}\right.$$

We will consider motions determined by exponentials of paths in the Lie algebra. Such a motion is determined by a unit speed space-curve τ(t). Now in a Frenet-Serret motion a point in the moving body moves along the curve and the coordinate frame in the moving body remains aligned with the tangent $\vec{t}$, normal $\vec{n}$, and binormal $\vec{b}$, of the curve. Using the 4-dimensional representation of the Lie group SE(3), the motion can be specified as:

(8) $$G(t)=\left(\begin{array}{cc} R(t) & \tau (t)\\ 0 & 1 \end{array}\right)\in SE(3)$$

where τ(t) is the curve and the rotation matrix R(t) has the unit vectors $\vec{t}$, $\vec{n}$, and $\vec{b}$ as columns:

(9) $$R(t)=\left(\begin{array}{ccc} \vec{t} & \vec{n} & \vec{b} \end{array}\right)\in SO(3)$$

If we introduce the Darboux vector $\vec{\omega }=\gamma \vec{t}+\kappa \vec{b}$ that we can rewritte from Frenet-Serret Formulas:

(10) $$\frac{d\vec{t}}{dt}=\vec{\omega }\times \vec{t}\;,\;\frac{d\vec{n}}{dt}=\vec{\omega }\times \vec{n}\;,\;\frac{d\vec{b}}{dt}=\vec{\omega }\times \vec{b}$$

Then, we can write with Ω is the 3 × 3 anti-symmetric matrix corresponding to $\vec{\omega }$:

(11) $$\frac{dR}{dt}=\Omega R$$

We note that $\frac{d\tau (t)}{dt}=\vec{t}$ and $\frac{d\vec{\omega }}{dt}=\frac{d\gamma }{dt}\vec{t}+\frac{d\kappa }{dt}\vec{b}$.

The instantaneous twist of the motion G(t) is given by:

(12) $$S_{d}=\frac{dG(t)}{dt}G^{-1}(t)=\left(\begin{array}{cc} \Omega & \upsilon \\ 0 & 0 \end{array}\right)$$

This is the Lie algebra element corresponding to the tangent vector to the curve G(t). It is well known that elements of the Lie algebra se(3) can be described as lines with a pitch. The fixed axode of a motion G(t)∈SE(3) is given by the axis of $S_{d}$ as t varies. The instantaneous twist in the moving reference frame is given by $S_{b}=G^{-1}(t)S_{d}G(t)$, that is, by the adjoint action on the twist in the fixed frame. The instantaneous twist $S_{b}$ can also be found from the relation:

(13) $$S_{b}=G^{-1}(t)\frac{dG(t)}{dt}$$

(14) $$S_{b}=G^{-1}\frac{dG}{dt}=\left(\begin{array}{cc} R^{T} & -R^{T}\tau \\ 0 & 1 \end{array}\right)\left(\begin{array}{cc} \Omega R & \vec{t}\\ 0 & 0 \end{array}\right)=\left(\begin{array}{cc} R^{T}\Omega R & R\vec{t}\\ 0 & 0 \end{array}\right)$$

We can observe that we could describe a 3D trajectory by a time series of *SE(3)* Lie group elements:

(15) $$SE(3)=\left\{\left[\begin{array}{cc} R & \tau \\ 0 & 1 \end{array}\right]/R\in SO(3),\tau \in R^{3}\right\}$$

with

(16) $$SO(3)=\left\{R/R^{T}R=RR^{T}=I,\det^{2}R=1\right\}$$

Then, the trajectory will be given by the following time series of *SE(3)* elements:

(17) $$\left\{\left[\begin{array}{cc} R_{1} & \tau_{1}\\ 0 & 1 \end{array}\right],\left[\begin{array}{cc} R_{2} & \tau_{2}\\ 0 & 1 \end{array}\right],\ldots ,\left[\begin{array}{cc} R_{n} & \tau_{n}\\ 0 & 1 \end{array}\right]\right\}\in SE{\left(3\right)}^{n}$$

## 2. New Results Introduced in the Paper

The paper is structured in two parts:

- ***1st Part on “Gauss Density on Lie groups”***: This part is totally new in Machine learning with an extension of “Gauss densities” (defined as Maximum Entropy model) for Lie groups coupling both Souriau model (introduced in statistical physics domain), with Information Geometry in Geometric Machine Learning domain. We illustrate with two use-cases SU(1,1) and SE(2) that are the most useful Lie groups in Image Processing (Sub-Riemannian Geometry of vision with SE(2)), in robotics (rigid bodies statistical analysis with SE(2)), in Natural Langage Processing (SU(1,1) with methods of graph-embedding in Poincaré disk), …. Some tentatives have been developed to define noise on Lie groups by adding additional Gaussian components on elements of the Lie algebra [26,27,28,29], but these models are not mathematically correct because they do not preserve the symmetries and the moment map associated to these symmetries by the Noether Theorem.
- ***2nd part on “Entropy definition extension as Casimir Function”*:** This part gives a new geometric definition of Entropy as invariant Casimir function in coadjoint representation, explaining the invariance of entropy under the affine coadjoint action on moment map in the dual space of Lie algebra. This definition was not in the paper of Souriau. With this new definition, we can compute Entropy only by structure constraints given by the Lie group. It opens the door to new generalization of Maximum Entropy method and first of all computation of “Gaussian densities” for any Lie group. Applications of this new property is not developed in this paper but in a twin paper in the same special issue [17]. We refert to M. Gromov papers to consider more geometric structures of Entropy [30,31].

The main new results of this paper are the introduction of “Gauss density” for Lie groups or data on homogeneous space where a Lie groups acts transitively, and the full computation for SU(1,1) Lie group. This group acts transitively on the Poincaré unit disk, and so we have also solved an open problem related to Gauss density on this homogeneous space. For this purpose, the main approach has considered an extended definition of classical “Gauss density”, as introduced by Jaynes, in term of density of Maximum Entropy. In this way, the initial problem was transfert to a new one related to the good definition of Entropy for Lie groups. To address this problem, first, we have recalled the classical Euclidean case, where the Entropy S(η) could be defined as the Legendre transform of minus the log-partition function $\Phi (\theta )=-\log\int_{R}e^{-\langle \theta ,y\rangle }dy$ (defined by Laplace transform) by the following equation $S(\eta )=\langle \theta ,\eta \rangle -\Phi (\theta )\;\mathrm{with}\;\eta_{i}=\frac{\partial \Phi (\theta )}{\partial \theta_{i}}\;\mathrm{and}\;\theta_{i}=\frac{\partial S(\eta )}{\partial \eta_{i}}$. The next step was to explain how to extend the log-partition function for Lie groups. We have then considered the Laplace transform in the framework of Lie group representation theory as introduced by Alexander Kirillov and Geometric Statical Mechanics as modeled by Jean-Marie Souriau. We have preserved the same Legendre structure, and have defined the Entropy S(Q), parametrized on the dual space of the Lie algebra $Q\in g^{*}$ (called geometric heat)**,** as Legendre transform of minus of the log-partition function $\Phi (\beta )=-\log\int_{M}e^{-\langle J(\xi ),\beta \rangle }d\lambda_{\omega }$, parametrized on the Lie algebra by β∈g (called geometric Planck Temperature), from a Laplace transform defined on the homogeneous symplectic manifold (associated to the Lie group by the Kirrilov-Kostant-Souriau 2-form called KKS 2-form in the litterature). By introducing the moment map $J:M\to g^{*}$, fundamental tool of representation theory introduced by Souriau, we were able to define the log-partition function on the coadjoint orbit of the Lie group, $\Phi (\beta )=-\log\int_{g^{*}}e^{-\langle J(\xi ),\beta \rangle }d\lambda_{\omega }$. The entropy is then given by the Legendre transform $S(Q)=\langle Q,\beta \rangle -\Phi (\beta )\;\mathrm{with}\;Q=\frac{\partial \Phi (\beta )}{\partial \beta }\in g^{*}\;\mathrm{and}\;\beta =\frac{\partial S(Q)}{\partial Q}\in g$. We have then defined the Gauss density for Lie groups as the density that maximizes this Entropy S(Q) under the constraint of its associated first moment $Q=\frac{\partial \Phi (\beta )}{\partial \beta }=\int_{M}J(\xi )p_{Gibbs}(\xi )d\lambda_{\omega }$. The Gauss density is then established by analogy with thermodynamics as the Gibbs density $p_{Gibbs}(\xi )=e^{\Phi (\beta )-\langle J(\xi ),\beta \rangle }=\frac{e^{-\langle J(\xi ),\beta \rangle }}{\int_{M}e^{-\langle J(\xi ),\beta \rangle }d\lambda_{\omega }}$. But this is not enough, because this density is not given in the good parametrization. We have proposed to express the Gibbs density with respect to the 1st statistical moment Q (statistical mean of moment map) by inverting the relation $Q=\frac{\partial \Phi (\beta )}{\partial \beta }=\Theta \left(\beta \right)$. The Gibbs density $p_{Gibbs,Q}(\xi )=e^{\Phi (\beta )-\langle J(\xi ),\Theta^{-1}\left(Q\right)\rangle }$ with $\beta =\Theta^{-1}\left(Q\right)$ will provide the extended definition of Gauss density in final good parametrization.

For the time being, no “Gaussian density” was defined on Poincaré unit disk with the mandatory property to be covariant under the action of *SU(1,1)* Lie group that acts transitively on this homogeneous bounded domain. We have applied the previous model via computation of moment map and developed the full computation of this extended Gauss density for *SU(1,1)* Lie group, $SU(1,1)=\left\{\left(\begin{array}{cc} a & b\\ b^{*} & a^{*} \end{array}\right)/a,b\in C,\;{\left|a\right|}^{2}-{\left|b\right|}^{2}=1\right\}$ and then deduced as consequence the gauss density for the Poincaré unit disk considered as the homogeneous symplectic manifold associated to the coadjoint orbit of the *SU(1,1)* Lie group via KKS 2 form. Considering the Lie algebra $su(1,1)=\left\{\left(\begin{array}{cc} ir & \eta \\ \eta^{*} & -ir \end{array}\right)/r\in R,\eta \in C\right\}$ and the dual space of the Lie algebra $su{\left(1,1\right)}^{*}=\left\{\left(\begin{array}{cc} z & x+iy\\ -x+iy & -z \end{array}\right)/x,y,z\in R\right\}$, we have computed the moment map $J:D\to su^{*}(1,1)$ defined by $J(z).u_{i}=J_{i}(z,z^{*})$, that maps D the Poincaré unit disk into a coadjoint orbit in $su^{*}(1,1)$, $J(z)=J_{1}\left(z,z^{*}\right)u_{1}^{*}+J_{2}\left(z,z^{*}\right)u_{2}^{*}+J_{3}\left(z,z^{*}\right)u_{3}^{*}=\rho \left(\begin{array}{cc} \frac{1+{\left|z\right|}^{2}}{1-{\left|z\right|}^{2}} & -2\frac{z^{*}}{1-{\left|z\right|}^{2}}\\ 2\frac{z}{1-{\left|z\right|}^{2}} & -\frac{1+{\left|z\right|}^{2}}{1-{\left|z\right|}^{2}} \end{array}\right)\in g^{*}$ The moment map J is a diffeomorphism of D onto one sheet of the two-sheeted hyperboloid in $su^{*}(1,1)$, determined by the following equation $J_{1}^{2}-J_{2}^{2}-J_{3}^{2}=\rho^{2}\;,\;J_{1}\geq \rho \;\mathrm{with}$ $J_{1}u_{1}^{*}+J_{2}u_{2}^{*}+J_{3}u_{3}^{*}\in su^{*}(1,1)$. But the full *SU(1,1)* Lie group is not related to any equilibrium Gibbs state (the open subset of the Lie algebra, associated to this Gibbs state is empty). We have then considered one-parameter subgroups of the Lie group SU(1,1) such that the open subset $\Lambda_{\beta }=\left\{\beta \in g/\int_{D}e^{-\langle J(z),\beta \rangle }d\lambda (z)<+\infty \right\}\;$ is not empty. In the neighborhood of the identity element, the elements of g∈SU(1,1) can be written as the exponential of an element β of its Lie algebra. If we make the remark that we have the following relation $\beta^{2}=\left(\begin{array}{cc} ir & \eta \\ \eta^{*} & -ir \end{array}\right)\left(\begin{array}{cc} ir & \eta \\ \eta^{*} & -ir \end{array}\right)=\left({\left|\eta \right|}^{2}-r^{2}\right)I$, we can developed the exponential map by a Taylor expansion of the exponential function, which is given by the following relation $g=\exp\left(\varepsilon \beta \right)=\sum_{k=0}^{\infty }\frac{{\left(\varepsilon \beta \right)}^{k}}{k!}=\left(\begin{array}{cc} \cosh\left(\varepsilon R\right)+ir\frac{\sinh\left(\varepsilon R\right)}{R} & \eta \frac{\sinh\left(\varepsilon R\right)}{R}\\ \eta^{*}\frac{\sinh\left(\varepsilon R\right)}{R} & \cosh\left(\varepsilon R\right)-ir\frac{\sinh\left(\varepsilon R\right)}{R} \end{array}\right)\mathrm{with}\;R^{2}={\left|\eta \right|}^{2}-r^{2}$. 

We can observe that one condition is that ${\left|\eta \right|}^{2}-r^{2}>0$ then the subset to consider is given by the subset $\Lambda_{\beta }=\left\{\beta =\left(\begin{array}{cc} ir & \eta \\ \eta^{*} & -ir \end{array}\right),r\in R,\eta \in C/{\left|\eta \right|}^{2}-r^{2}>0\right\}\;$ such that $\int_{D}e^{-\langle J(z),\beta \rangle }d\lambda (z)<+\infty$. Finally, we have computed the covariant Gibbs density in the unit disk given by $\beta \in \Lambda_{\beta }\;$ and by the moment map of the Lie group SU(1,1), that could be expressed in the following equation: $p_{Gibbs}\left(z\right)=\frac{e^{-\langle J\left(z\right),\beta \rangle }}{\int_{D}e^{-\langle J\left(z\right),\beta \rangle }d\lambda (z)}==\frac{e^{-\langle \rho \left(\begin{array}{cc} \frac{1+{\left|z\right|}^{2}}{\left(1-{\left|z\right|}^{2}\right)} & \frac{-2z^{*}}{\left(1-{\left|z\right|}^{2}\right)}\\ \frac{2z}{\left(1-{\left|z\right|}^{2}\right)} & -\frac{1+{\left|z\right|}^{2}}{\left(1-{\left|z\right|}^{2}\right)} \end{array}\right),\left(\begin{array}{cc} ir & \eta \\ \eta^{*} & -ir \end{array}\right)\rangle }}{\int_{D}e^{-\langle J\left(z\right),\beta \rangle }d\lambda (z)}\;\mathrm{with}\;d\lambda (z)=2i\rho \frac{dz\wedge dz^{*}}{{\left(1-{\left|z\right|}^{2}\right)}^{2}}$. To write the final Gibbs density with respect to its statistical moment, we rewrite the density with Q=E[J(z)], by $\beta =\Theta^{-1}\left(Q\right)\in g$ where $Q=\frac{\partial \Phi \left(\beta \right)}{\partial \beta }=\Theta \left(\beta \right)\in g^{*}$ and $Q=E\left[J(z)\right]=E\left[\rho \left(\begin{array}{cc} \frac{1+{\left|w\right|}^{2}}{\left(1-{\left|w\right|}^{2}\right)} & \frac{-2w^{*}}{\left(1-{\left|w\right|}^{2}\right)}\\ \frac{2w}{\left(1-{\left|w\right|}^{2}\right)} & -\frac{1+{\left|w\right|}^{2}}{\left(1-{\left|w\right|}^{2}\right)} \end{array}\right)\right]$.

To extend this approach for covariant Gibbs density on Siegel Unit Disk $SD=\left\{Z\in M_{pq}\left(C\right)/I_{p}-ZZ^{+}>0\right\}$, that is a classical matrix extension of Poincaré unit Disk, we have proposed to consider G=SU(p,q) unitary group and the homogeneous space $G/K=SU(p,q)/S\left(U(p),U(q)\right)\;\mathrm{with}\;K=S\left(U(p)\times U(q)\right)=\left\{\left(\begin{array}{cc} A & 0\\ 0 & D \end{array}\right)/A\in U(p),D\in U(q),\det(A)\det(D)=1\right\}$ and the moment map given by $J\left(Z\right)=i\lambda \left(\begin{array}{cc} {\left(I_{p}-ZZ^{+}\right)}^{-1}\left(-pZZ^{+}-qI_{p}\right) & \left(p+q\right)Z{\left(I_{q}-Z^{+}Z\right)}^{-1}\\ -(p+q){\left(I_{q}-Z^{+}Z\right)}^{-1}Z^{+} & \left(pI_{q}+qZ^{+}Z\right){\left(I_{q}-Z^{+}Z\right)}^{-1} \end{array}\right)$.

After SU(1,1) Lie group (case with null cohomology), we have considered the same model for SE(2) Lie group with non-null cohomology that needs the use of symplectic one-cocycle to manage the defect of cohomology. We have considered the special Euclidean group $SE(2)=\left\{\left[\begin{array}{cc} R_{\varphi } & \tau \\ 0 & 1 \end{array}\right]/R_{\varphi }\in SO\left(2\right),\tau \in R^{2}\right\}$ with $SO(2)=\left\{R_{\varphi }=\left[\begin{array}{cc} \cos\varphi & -\sin\varphi \\ \sin\varphi & \cos\varphi \end{array}\right]/\varphi \in R\right\}$, and the Lie algebra se(2) of SE(2) $\left(\xi ,u\right)\in se(2)=R\times R^{2}\Rightarrow \left[\begin{array}{cc} -\xi ℑ & u\\ 0 & 0 \end{array}\right]\in se\left(2\right)$ with $ℑ=\left[\begin{array}{cc} 0 & 1\\ -1 & 0 \end{array}\right]$, to define the moment map $J_{\left(\xi ,u\right)}(x):R^{2}\to se^{*}\left(2\right)$ that is given by the expression $J_{\left(\xi ,u\right)}(x)=J(x).\left(\xi ,u\right)$ with $J(x)=-2\left(\frac{1}{2}{\left\|x\right\|}^{2},-ℑx\right),\;x\in R^{2}$. Then, the Gibbs density is deduced for generalized temperature $\beta \in \Omega =\left\{\left(b,Β\right)\in se(2)/b<0,Β\in R^{2}\right\}$ by $p_{Gibbs}\left(x\right)=\frac{e^{-\langle J\left(x\right),\beta \rangle }}{\int_{R^{2}}e^{-\langle J\left(x\right),\beta \rangle }d\lambda (x)}=\frac{e^{\frac{1}{2}b{\left\|x\right\|}^{2}-Β.ℑx}}{\int_{R^{2}}e^{\frac{1}{2}b{\left\|x\right\|}^{2}-Β.ℑx}d\lambda (x)}$, with the log-partition function given by the following expression $\Phi \left(\beta \right)=\log\int_{R^{2}}e^{\frac{1}{2}b{\left\|x\right\|}^{2}-Β.ℑx}d\lambda (x)=\log\left(-\frac{2\pi }{b}e^{-\frac{1}{2b}{\left\|B\right\|}^{2}}\right)$ with $Q=\frac{\partial \Phi \left(\beta \right)}{\partial \beta }=\left(\frac{1}{b}-\frac{{\left\|Β\right\|}^{2}}{2b^{2}},\frac{1}{b}Β\right)=\Theta \left(\beta \right)$ and where $Q\in \Omega^{*}=\left\{\left(m,M\right)\in se^{*}(2)/m+\frac{{\left\|M\right\|}^{2}}{2}<0\right\}$. To obtain the good parametrization related to statical moments, we have inverted the relation $\beta =\Theta^{-1}\left(Q\right)=\left({\left(m+\frac{1}{2}{\left\|M\right\|}^{2}\right)}^{-1},{\left(m+\frac{1}{2}{\left\|M\right\|}^{2}\right)}^{-1}M\right)$, to provide the covariant Gibbs density parametrized by $\left(m,M\right)=E\left(J(x)\right)=E\left[-2\left(\frac{1}{2}{\left\|x\right\|}^{2},-ℑx\right)\right]=\left[-E\left({\left\|x\right\|}^{2}\right),2ℑE(x)\right]$. The final Gauss density for *SE(2)* is then $p_{Gibbs}\left(x\right)=\frac{e^{\frac{\frac{1}{2}{\left\|x\right\|}^{2}-M.ℑx}{\left(m+\frac{1}{2}{\left\|M\right\|}^{2}\right)}}}{\int_{R^{2}}e^{\frac{\frac{1}{2}{\left\|x\right\|}^{2}-M.ℑx}{\left(m+\frac{1}{2}{\left\|M\right\|}^{2}\right)}}d\lambda (x)}$.

We conclude the paper by a deeper study of Souriau model structure. We observe that Souriau Entropy S(Q) defined on coadjoint orbit of the group has a property of invariance $S\left(Ad_{g}^{\#}(Q)\right)=S\left(Q\right)$ with respect to Souriau affine definition of coadjoint action $Ad_{g}^{\#}(Q)=Ad_{g}^{*}(Q)+\theta \left(g\right)$ where θ(g) is called the Souriau cocyle. In the framework of Souriau Lie groups Thermodynamics, we can then characterize the Entropy as a generalized Casimir invariant function in coadjoint representation, and Massieu characteristic function (or log-partition function), dual of Entropy by Legendre transform, as a generalized Casimir function in adjoint representation. When *M* is a Poisson manifold, a function on *M* is a Casimir function if and only if this function is constant on each symplectic leaf (the non-empty open subsets of the symplectic leaves are the smallest embedded manifolds of *M* which are Poisson submanifolds) [15]. Classically, the Entropy is defined axiomatically as Shannon or von Neumann Entropies without any geometric structures constraints. In this paper, the Entropy is also presented as solution of the Casimir equation ${\left(ad_{\frac{\partial S}{\partial Q}}^{*}Q\right)}_{j}+\Theta {\left(\frac{\partial S}{\partial Q}\right)}_{j}=C_{ij}^{k}ad_{{\left(\frac{\partial S}{\partial Q}\right)}^{i}}^{*}Q_{k}+\Theta_{j}=0$ with $\tilde{\Theta }\left(X,Y\right)=\langle \Theta (X),Y\rangle =J_{\left[X,Y\right]}-\left\{J_{X},J_{Y}\right\}=-\langle d\theta (X),Y\rangle$, X,Y∈g, where $\Theta (X)=T_{e}\theta \left(X(e)\right)$ appears in case of non-null cohomology (non-equivariance of coadjoint operator on the moment map), with θ(g) the Souriau Symplectic cocycle. The dual space of the Lie algebra foliates into coadjoint orbits that are also the level sets on the entropy. The KKS (Kostant-Kirillov Souriau) 2-form, and the Souriau-Koszul-Fisher metric transform each orbit into a homogeneous Symplectic manifold. The information manifold foliates into level sets of the entropy that could be interpreted in the framework of Thermodynamics by the fact that motion remaining on this complex surfaces is non-dissipative, whereas motion transversal to these surfaces is dissipative, where the dynamic is given by $\frac{dQ}{dt}={\left\{Q,H\right\}}_{\tilde{\Theta }}=ad_{\frac{\partial H}{\partial Q}}^{*}Q+\Theta \left(\frac{\partial H}{\partial Q}\right)$ with stable equilibrium when $H=S\Rightarrow \frac{dQ}{dt}={\left\{Q,S\right\}}_{\tilde{\Theta }}=ad_{\frac{\partial S}{\partial Q}}^{*}Q+\Theta \left(\frac{\partial S}{\partial Q}\right)=0$. We have finally also observed that $dS={\tilde{\Theta }}_{\beta }\left(\frac{\partial H}{\partial Q},\beta \right)dt$ where ${\tilde{\Theta }}_{\beta }\left(\frac{\partial H}{\partial Q},\beta \right)=\tilde{\Theta }\left(\frac{\partial H}{\partial Q},\beta \right)+\langle Q,\left[\frac{\partial H}{\partial Q},\beta \right]\;\rangle$, showing that Entropy production is linked with Souriau tensor related to Fisher metric.

The Casimir equations that we have introduced in non-zero cohomology case are consequences of the constancy of the entropy on adjoint orbits of the Lie algebra and of the equivariance of the map between the set of generalized temperatures and the dual space of the Lie algebra, as introduced by Jean-Marie in his 1974 paper. We explained this fact in the paper by starting elaboration of Casimir equations from the Souriau equation. Casimir equations are then presented in this context, as a fully equivalent form written in a new way, especially in the framework of Souriau Lie groups Thermodynamics. Souriau has not observed that the Entropy is an invariant Casimir function in coadjoint representation, but we can assume that he was fully aware of this invariant structure.

From Souriau equation $\langle Q,\left[\beta ,Z\right]\rangle +\tilde{\Theta }\left(\beta ,Z\right)=0$ published in 1974, we have rewritten as direct consequence this equation on a Casimir form $ad_{\frac{\partial S}{\partial Q}}^{*}Q+\Theta \left(\frac{\partial S}{\partial Q}\right)=0$. This equation preserves the geometric structures included in Souriau equation but allow us to consider the Entropy from the point of view of Casimir invariant function. The concept of Entropy and the concept of Casimir function were, for the time being, two disjoint concepts that have been developed independently in the past. There is a large literature on Casimir function, especially the russian one that have characterized properties of Casimir function. We refer to Igor V. Shirokov who has proposed a method for constructing invariants of the coadjoint representation of Lie groups with an arbitrary dimension and structure based on local symplectic coordinates on the coadjoint orbits. With Oleg L. Kurnyavko, Igor V. Shirokov has also proposed a general method for constructing invariant Casimir functions. The second reference is about A.T. Fomenko and V.V. Trofimov who have also deeply studied Casimir functions (but in case of null cohomology) and have developed the following equation that we can write for Entropy in null cohomology case $S\left(Ad_{e^{t\xi }}^{*}Q\right)=S(Q)+\sum_{n=1}^{\infty }\frac{{\left(-\phi (\xi )\right)}^{n}S}{n!}(Q).t^{n}$ with ϕ:g→Vec(Γ) a representation of Lie algebras defined on basis $\left(e_{1},e_{2},\ldots ,e_{n}\right)$ in g. We refer to a twin paper [17] developing consequences of this new definition of Entropy as an invariant Casimir function. In this twin paper, we study the associated Euler-Poincaré equation $\frac{dQ}{dt}=ad_{\frac{\partial H}{\partial Q}}^{*}Q+\Theta \left(\frac{\partial H}{\partial Q}\right)$ and the stochastic extension based on a new Stratonovich differential equation for the stochastic process given by the following relation by mean of Souriau’s symplectic cocycle $dQ+\left[ad_{\frac{\partial H}{\partial Q}}^{*}Q+\Theta \left(\frac{\partial H}{\partial Q}\right)\right]dt+\sum_{i=1}^{N}\left[ad_{\frac{\partial H_{i}}{\partial Q}}^{*}Q+\Theta \left(\frac{\partial H_{i}}{\partial Q}\right)\right]\circ dW_{i}\left(t\right)=0$. These kind of stochastic equations have been also studied by Alexis Arnaudon and Daryl Holm but only in the restricted case of null-cohomology [32].

We give references from classical textbooks (as Souriau book and papers) to preprints because different approaches have been developed in parallel to address Lie groups statistics, as soon as mid of last century, but without bridges between these disciplines which have developed specific tools to address this problem. We have limited these references to main and important documents, which are characterized as seminal and as tutorial of their domains. We have preserved references in French, because some works as Souriau Lie groups Thermodynamics model have not been yet largely spread towards the different communities.

## 3. Learning Inference Lie Groups Thermodynamics and Covariant Gibbs Density

We identify the Riemanian metric introduced by Souriau based on cohomology, in the framework of “Lie groups thermodynamics” as an extension of classical Fisher metric introduced in information geometry. We have observed that Souriau metric preserves Fisher metric structure as the Hessian of the minus logarithm of a partition function, where the partition function is defined as a generalized Laplace transform on a sharp convex cone. Souriau’s definition of Fisher metric extends the classical one in case of Lie groups or homogeneous manifolds. Souriau has developed this “Lie groups thermodynamics” theory in the framework of homogeneous symplectic manifolds in geometric statistical mechanics for dynamical systems, but as observed by Souriau, these model equations are no longer linked to the symplectic manifold but equations only depend on the Lie group and the associated cocycle [33,34]. This analogy with Fisher metric opens potential applications in machine learning, where the Fisher metric is used in the framework of information geometry, to define the “natural gradient” tool for improving ordinary stochastic gradient descent sensitivity to rescaling or changes of variable in parameter space. In machine learning revised by natural gradient of information geometry, the ordinary gradient is designed to integrate the Fisher matrix. Amari has theoretically proved the asymptotic optimality of the natural gradient compared to classical gradient. With the Souriau approach, the Fisher metric could be extended, by Souriau-Fisher metric, to design natural gradients for data on homogeneous manifolds. Information geometry has been derived from invariant geometrical structure involved in statistical inference. The Fisher metric defines a Riemannian metric as the Hessian of two dual potential functions, linked to dually coupled affine connections in a manifold of probability distributions. With the Souriau model, this structure is extended preserving the Legendre transform between two dual potential function parametrized in Lie algebra of the group acting transentively on the homogeneous manifold.

### 3.1. Inference by Natutal Gradient and Legendre Structure

Classically, to optimize the parameter θ of a probabilistic model, based on a sequence of observations $y_{t}$, is an online gradient descent:

(18) $$\theta_{t}\leftarrow \theta_{t-1}-\eta_{t}\frac{\partial l_{t}{\left(y_{t}\right)}^{T}}{\partial \theta }$$

with learning rate $\eta_{t}$, and the loss function $l_{t}=-\log p\left(y_{t}/{\hat{y}}_{t}\right)$. This simple gradient descent has a first drawback of using the same non-adaptive learning rate for all parameter components, and a second drawback of non invariance with respect to parameter re-encoding inducing different learning rates. Amari has introduced the natural gradient to preserve this invariance to be insensitive to the characteristic scale of each parameter direction. The gradient descent could be corrected by $I{\left(\theta \right)}^{-1}$ where I is the Fisher information matrix with respect to parameter θ, given by:

(19) $$I\left(\theta \right)=\left[g_{ij}\right]\;\mathrm{with}\;g_{ij}={\left[-E_{y∼p(y/\theta )}\left[\frac{\partial^{2}\log p\left(y/\theta \right)}{\partial \theta_{i}\partial \theta_{j}}\right]\right]}_{ij}$$

with natural gradient:

(20) $$\theta_{t}\leftarrow \theta_{t-1}-\eta_{t}I{\left(\theta \right)}^{-1}\frac{\partial l_{t}{\left(y_{t}\right)}^{T}}{\partial \theta }$$

Amari has proved that the Riemannian metric in an exponential family is the Fisher information matrix defined by:

(21) $$g_{ij}=-{\left[\frac{\partial^{2}\Phi }{\partial \theta_{i}\partial \theta_{j}}\right]}_{ij}\;\mathrm{with}\;\Phi (\theta )=-\log\int_{R}e^{-\langle \theta ,y\rangle }dy$$

and the dual potential, the Shannon entropy, is given by the Legendre transform:

(22) $$S(\eta )=\langle \theta ,\eta \rangle -\Phi (\theta )\;\mathrm{with}\;\eta_{i}=\frac{\partial \Phi (\theta )}{\partial \theta_{i}}\;\mathrm{and}\;\theta_{i}=\frac{\partial S(\eta )}{\partial \eta_{i}}$$

We can observe that $\Phi (\theta )=-\log\int_{R}e^{-\langle \theta ,y\rangle }dy=-\log\psi \left(\theta \right)$ is linked with the cumulant generating function.

J.L. Koszul and E. Vinberg have introduced an affinely invariant Hessian metric on a sharp convex cone through its characteristic function:

(23) $$\begin{array}{l} \Phi_{\Omega }(\theta )=-\log\int_{\Omega^{*}}e^{-\langle \theta ,y\rangle }dy=-\log\psi_{\Omega }(\theta )\;\mathrm{with}\;\theta \in \Omega \;\mathrm{sharp}\;\mathrm{convex}\;\mathrm{cone}\\ \psi_{\Omega }(\theta )=\int_{\Omega^{*}}e^{-\langle \theta ,y\rangle }dy\;\mathrm{with}\;\mathrm{Koszul-Vinberg}\;\mathrm{Characteristic}\;\mathrm{function} \end{array}$$

Jean-Louis Koszul has introduced the following forms

1st Koszul form:

(24) $$\alpha =d\Phi_{\Omega }(\theta )=-d\log\psi_{\Omega }(\theta )$$

2nd Koszul form:

(25) $$\gamma =D\alpha =Dd\log\psi_{\Omega }(\theta )$$

with the following property of positive definitiveness:

(26) $$\begin{array}{l} \left(Dd\log\psi_{\Omega }(x)\right)(u)=\frac{1}{\psi_{\Omega }{\left(u\right)}^{2}}\left[\int_{\Omega^{*}}F{\left(\xi \right)}^{2}d\xi .\int_{\Omega^{*}}G{\left(\xi \right)}^{2}d\xi -{\left(\int_{\Omega^{*}}F(\xi ).G(\xi )d\xi \right)}^{2}\right]>0\\ \mathrm{with}\;F(\xi )=e^{-\frac{1}{2}\langle x,\xi \rangle }\;\mathrm{and}\;G(\xi )=e^{-\frac{1}{2}\langle x,\xi \rangle }\langle u,\xi \rangle \end{array}$$

Koszul has defined the following Diffeomorphism:

(27) $$\eta =\alpha =-d\log\psi_{\Omega }(\theta )=\int_{\Omega^{*}}\xi p_{\theta }(\xi )d\xi \;\mathrm{with}\;p_{\theta }(\xi )=\frac{e^{-\langle \xi ,\theta \rangle }}{\int_{\Omega^{*}}e^{-\langle \xi ,\theta \rangle }d\xi }$$

with preservation of Legendre transform:

(28) $$S_{\Omega }(\eta )=\langle \theta ,\eta \rangle -\Phi_{\Omega }(\theta )\;\mathrm{with}\;\eta =d\Phi_{\Omega }(\theta )\;\mathrm{and}\;\theta =dS_{\Omega }(\eta )$$

### 3.2. Souriau Lie Groups Thermodynamique and Souriau-Koszul-Fisher Metric

This relations have been extended by Jean-Marie Souriau in geometric statistical mechanics, where he developed a “Lie groups thermodynamics” of dynamical systems where the (maximum entropy) Gibbs density is covariant with respect to the action of the Lie group. In the Souriau model, previous structures of information geometry are preserved:

(29) $$I(\beta )=-\frac{\partial^{2}\Phi }{\partial \beta^{2}}\;\mathrm{with}\;\Phi (\beta )=-\log\int_{M}e^{-\langle U(\xi ),\beta \rangle }d\lambda_{\omega }\;\mathrm{and}\;U:M\to g^{*}$$

(30) $$S(Q)=\langle Q,\beta \rangle -\Phi (\beta )\;\mathrm{with}\;Q=\frac{\partial \Phi (\beta )}{\partial \beta }\in g^{*}\;\mathrm{and}\;\beta =\frac{\partial S(Q)}{\partial Q}\in g$$

In the Souriau Lie groups thermodynamics model, β is a “geometric” (Planck) temperature, element of Lie algebra g of the group, and Q is a “geometric” heat, element of the dual space of the Lie algebra $g^{*}$ of the group. Souriau has proposed a Riemannian metric that we have identified as a generalization of the Fisher metric:

(31) $$I\left(\beta \right)=\left[g_{\beta }\right]\;\mathrm{with}\;g_{\beta }\left(\left[\beta ,Z_{1}\right],\left[\beta ,Z_{2}\right]\right)={\tilde{\Theta }}_{\beta }\left(Z_{1},\left[\beta ,Z_{2}\right]\right)$$

(32) $$\mathrm{with}\;{\tilde{\Theta }}_{\beta }\left(Z_{1},Z_{2}\right)=\tilde{\Theta }\left(Z_{1},Z_{2}\right)+\langle Q,ad_{Z_{1}}(Z_{2})\;\rangle \;\mathrm{where}\;ad_{Z_{1}}(Z_{2})=\left[Z_{1},Z_{2}\right]$$

Souriau has proved that all co-adjoint orbit of a Lie group given by $O_{F}=\left\{Ad_{g}^{*}F=g^{-1}Fg,g\in G\right\}\mathrm{subset}\;\mathrm{of}\;g^{*},F\in g^{*}$ carries a natural homogeneous symplectic structure by a closed *G*-invariant 2-form. If we define $K=Ad_{g}^{*}={\left(Ad_{g^{-1}}\right)}^{*}$ and $K_{*}(X)=-{\left(ad_{X}\right)}^{*}$ with $\langle Ad_{g}^{*}F,Y\rangle =\langle F,Ad_{g^{-1}}Y\rangle ,\forall g\in G,Y\in g,F\in g^{*}$ where if X∈g, $Ad_{g}(X)=gXg^{-1}\in g$, the G-invariant 2-form is given by the following expression $\sigma_{\Omega }\left(ad_{X}F,ad_{Y}F\right)=B_{F}\left(X,Y\right)=\langle F,\left[X,Y\right]\rangle ,X,Y\in g$. Souriau Foundamental Theorem is that « Every symplectic manifold on which a Lie group acts transitively by a Hamiltonian action is a covering space of a coadjoint orbit ». We can observe that for Souriau model, Fisher metric is an extension of this 2-form in non-equivariant case $g_{\beta }\left(\left[\beta ,Z_{1}\right],\left[\beta ,Z_{2}\right]\right)=\tilde{\Theta }\left(Z_{1},\left[\beta ,Z_{2}\right]\right)+\langle Q,\left[Z_{1},\left[\beta ,Z_{2}\right]\right]\;\rangle$.

The Souriau additional term $\tilde{\Theta }\left(Z_{1},\left[\beta ,Z_{2}\right]\right)$ is generated by non-equivariance through Symplectic cocycle. The tensor $\tilde{\Theta }$ used to define this extended Fisher metric is defined by the moment map J(x), application from M (homogeneous symplectic manifold) to the dual space of the Lie algebra $g^{*}$, given by:

(33) $$\tilde{\Theta }(X,Y)=J_{\left[X,Y\right]}-\left\{J_{X},J_{Y}\right\}$$

(34) $$\mathrm{with}\;J(x):M\to g^{*}\;\mathrm{such}\;\mathrm{that}\;J_{X}(x)=\langle J(x),X\rangle ,\;X\in g$$

This tensor $\tilde{\Theta }$ is also defined in tangent space of the cocycle $\theta \left(g\right)\in g^{*}$ (this cocycle appears due to the non-equivariance of the coadjoint operator $Ad_{g}^{*}$, action of the group on the dual space of the lie algebra; the action of the group on the dual space of the Lie algebra is modified with a cocycle so that the momentu map becomes equivariant relative to this new affine action):

(35) $$Q\left(Ad_{g}(\beta )\right)=Ad_{g}^{*}(Q)+\theta \left(g\right)$$

$\theta \left(g\right)\in g^{*}$ is called nonequivariance one-cocycle, and it is a measure of the lack of equivariance of the moment map.

(36) $$\begin{array}{l} \tilde{\Theta }\left(X,Y\right):g\times g\to ℜ\;\mathrm{with}\;\Theta (X)=T_{e}\theta \left(X(e)\right)\\ \;X,Y\mapsto \langle \Theta (X),Y\rangle \end{array}$$

The cocycle should verify:

(37) $$\begin{array}{l} \theta (st)=J(\left(st\right).x)-Ad_{st}^{*}J(x)\\ \theta (st)=\left[J(s.\left(t.x\right))-Ad_{s}^{*}J\left(t.x\right)\right]+\left[Ad_{s}^{*}J\left(t.x\right)-Ad_{s}^{*}Ad_{t}^{*}J(x)\right]\\ \theta (st)=\theta (s)+Ad_{s}^{*}\left[J\left(t.x\right)-Ad_{t}^{*}J(x)\right]\\ \theta (st)=\theta (s)+Ad_{s}^{*}\theta (t) \end{array}$$

We can also compute tangent of one-cocycle θ at neutral element, to compute 2-cocycle Θ:

(38) $$\begin{array}{l} \zeta \in g,\theta_{\zeta }\left(s\right)=\langle \theta \left(s\right),\zeta \rangle =\langle J\left(s.x\right),\zeta \rangle -\langle Ad_{s}^{*}J\left(x\right),\zeta \rangle =\langle J\left(s.x\right),\zeta \rangle -\langle J\left(x\right),Ad_{s^{-1}}\zeta \rangle \\ T_{e}\theta_{\zeta }\left(\xi \right)=\langle T_{x}J.\xi_{p}(x),\zeta \rangle +\langle J\left(x\right),ad_{\xi }\zeta \rangle \;\mathrm{with}\;\xi_{p}=X_{\langle J,\xi \rangle }\\ T_{e}\theta_{\zeta }\left(\xi \right)=X_{\langle J(x),\xi \rangle }\left[\langle J(x),\zeta \rangle \right]+\langle J\left(x\right),\left[\xi ,\zeta \right]\rangle \\ T_{e}\theta_{\zeta }\left(\xi \right)=-\left\{\langle J,\xi \rangle ,\langle J,\zeta \rangle \right\}+\langle J(x),\left[\xi ,\zeta \right]\rangle =\Theta \left(\xi \right) \end{array}$$

We can also write: $T_{x}J\left(\xi_{p}(x)\right)=-ad_{\xi }^{*}J(x)+\Theta \left(\xi ,.\right)$

By differentiating the equation on affine action, we have:

(39) $$dJ(Xx)=ad_{X}J(x)+d\theta (X)\;,\;x\in M,X\in g$$

(40) $$\begin{array}{l} \langle dJ(Xx),Y\rangle =\langle ad_{X}J(x),Y\rangle +\langle d\theta (X),Y\rangle ,\;x\in M,X,Y\in g\\ \langle dJ(Xx),Y\rangle =\langle J(x),\left[X,Y\right]\rangle +\langle d\theta (X),Y\rangle =\left\{\langle J,X\rangle ,\langle J,Y\rangle \right\}(x)\\ \;\langle J(x),\left[X,Y\right]\rangle -\left\{\langle J,X\rangle ,\langle J,Y\rangle \right\}(x)=-\langle d\theta (X),Y\rangle \end{array}$$

It can be then deduced that the tensor could be also written:

(41) $$\tilde{\Theta }(X,Y)=J_{\left[X,Y\right]}-\left\{J_{X},J_{Y}\right\}=-\langle d\theta (X),Y\rangle \;,\;X,Y\in g$$

with the cocycle property:

(42) $$\tilde{\Theta }\left(\left[X,Y\right],Z\right)+\tilde{\Theta }\left(\left[X,Y\right],Z\right)+\tilde{\Theta }\left(\left[X,Y\right],Z\right)=0\;,\;X,Y,Z\in g$$

By noting the action of the group on the dual space of the Lie algebra:

(43) $$G\times g^{*}\to g^{*},(s,\xi )\mapsto s\xi =Ad_{s}^{*}\xi +\theta (s)$$

Associativity is also derived:

(44) $$\begin{array}{l} \left(s_{1}s_{2})\xi =Ad_{s_{1}s_{2}}^{*}\xi +\theta (s_{1}s_{2})=Ad_{s_{1}}^{*}Ad_{s_{2}}^{*}\xi +\theta (s_{1})+Ad_{s_{1}}^{*}\theta (s_{2}\right)\\ (s_{1}s_{2})\xi =Ad_{s_{1}}^{*}\left(Ad_{s_{2}}^{*}\xi +\theta (s_{2})\right)+\theta (s_{1})=s_{1}\left(s_{2}\xi \right)\;,\;\forall s_{1},s_{2}\in G,\xi \in g^{*} \end{array}$$

This study of the moment map J equivariance, and the existence of an affine action of *G* on $g^{*}$, whose linear part is the coadjoint action, for which the moment J is equivariant, is at the cornerstone of Souriau theory of geometric mechanics and Lie groups thermodynamics.

### 3.3. Souriau Entropy and Souriau-Fisher-Koszul Metric Invariance under the Action of the Group and Covariant Souriau Gibbs Density

In Souriau’s Lie groups thermodynamics, the invariance by re-parameterization in information geometry has been replaced by invariance with respect to the action of the group. When an element of the group g acts on the element β∈g of the Lie algebra, given by adjoint operator $Ad_{g}$. Under the action of the group $Ad_{g}(\beta )$, the entropy S(Q) and the Fisher metric I(β) are invariant:

(45) $$\beta \in g\to Ad_{g}(\beta )\Rightarrow \left\{ \begin{array}{l} S\left[Q\left(Ad_{g}(\beta )\right)\right]=S\left(Q\right)\\ I\left[Ad_{g}(\beta )\right]=I\left(\beta \right) \end{array}\right.$$

In the framework of Lie group action on a symplectic manifold, equivariance of moment map could be studied to prove that there is a unique action *a*(.,.) of the Lie group G on the dual $g^{*}$ of its Lie algebra for which the moment map J is equivariant, that means for each x∈M:

(46) $$J\left(\Phi_{g}(x)\right)=a(g,J(x))=Ad_{g}^{*}\left(J(x)\right)+\theta (g)$$

When coadjoint action is not equivariant, the symmetry is broken, and new “cohomological” relations should be verified in Lie algebra of the group. A natural equilibrium state will thus be characterized by an element of the Lie algebra of the Lie group, determining the equilibrium temperature β. The entropy s(Q), parametrized by Q the geometric heat (mean of energy U, element of the dual space of the Lie algebra) is defined by the Legendre transform of the Massieu potential Φ(β) parametrized by β (Φ(β) is the minus logarithm of the partition function $\psi_{\Omega }\left(\beta \right)$).

A Gibbs state, in the usual sense, is a statistical state at which the entropy is stationary with respect to all infinitesimal variations of the statistical state for which the mean value of the energy remains constant. In the sense of Souriau, a generalized Gibbs state is a statistical state at which the entropy is stationary with respect to all infinitesimal variations of the statistical state for which the mean value of the moment map remains constant. This generalization is very natural, since the energy can be considered as the moment map of the Hamiltonian action of the one-dimensional Lie group of time translations. Furthermore, each generalized Gibbs state is associated to an element of the Lie algebra of the group, called by Souriau a generalized temperature, and that the set of possible generalized temperature is not, in general the whole Lie algeba, but an open convex subset of the Lie algebra, which may be empty, for which some integrals encountered in the expression of the generalized Gibbs state are normally convergent. So, for some Lie groups, generalized Gibbs states do not exist, and there is no Souriau Lie groups thermodynamics.

Souriau has then defined a Gibbs density that is covariant under the action of the group:

(47) $$\begin{array}{l} p_{Gibbs}(\xi )=e^{\Phi (\beta )-\langle U(\xi ),\beta \rangle }=\frac{e^{-\langle U(\xi ),\beta \rangle }}{\int_{M}e^{-\langle U(\xi ),\beta \rangle }d\lambda_{\omega }}\;,\;\mathrm{with}\;\Phi (\beta )=-\log\int_{M}e^{-\langle U(\xi ),\beta \rangle }d\lambda_{\omega }\\ Q=\frac{\partial \Phi (\beta )}{\partial \beta }=\frac{\int_{M}U(\xi )e^{-\langle U(\xi ),\beta \rangle }d\lambda_{\omega }}{\int_{M}e^{-\langle U(\xi ),\beta \rangle }d\lambda_{\omega }}=\int_{M}U(\xi )p(\xi )d\lambda_{\omega } \end{array}$$

**Figure 3:**
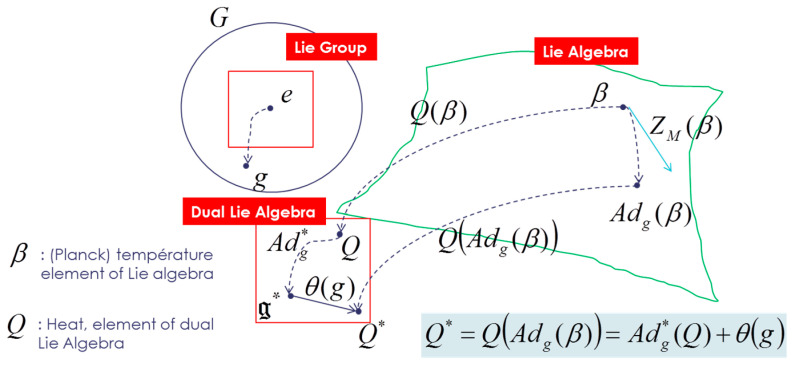
Fondamental Equation of Souriau Lie groups Thermodynamics. *Q* is the geometric heat in dual Lie algebra, *β* is the geometric temperature in Lie algebra.

**Figure 4:**
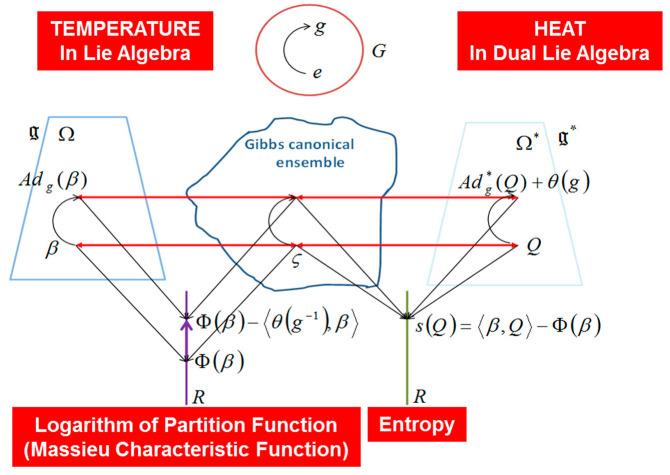
Souriau Model of Lie Groups Thermodynamics.

We can express the Gibbs density with respect to Q by inverting the relation $Q=\frac{\partial \Phi (\beta )}{\partial \beta }=\Theta \left(\beta \right)$. Then $p_{Gibbs,Q}(\xi )=e^{\Phi (\beta )-\langle U(\xi ),\Theta^{-1}\left(Q\right)\rangle }$ with $\beta =\Theta^{-1}\left(Q\right)$. All Souriau equations of Lie groups Thermodynamics are illustrated in Figure 3 and Figure 4.

Souriau completed his “geometric heat theory” by introducing a 2-form in the Lie algebra, that is a Riemannian metric tensor in the values of adjoint orbit of β, [β,Z] with Z an element of the Lie algebra. This metric is given for (β,Q):

(48) $$g_{\beta }\left(\left[\beta ,Z_{1}\right],\left[\beta ,Z_{2}\right]\right)=\langle \Theta \left(Z_{1}\right),\left[\beta ,Z_{2}\right]\rangle +\langle Q,\left[Z_{1},\left[\beta ,Z_{2}\right]\right]\rangle$$

where Θ is a cocycle of the Lie algebra, defined by $\Theta =T_{e}\theta$ with θ a cocycle of the Lie group defined by $\theta (M)=Q\left(Ad_{M}(\beta )\right)-Ad_{M}^{*}Q$.

**Figure 5:**
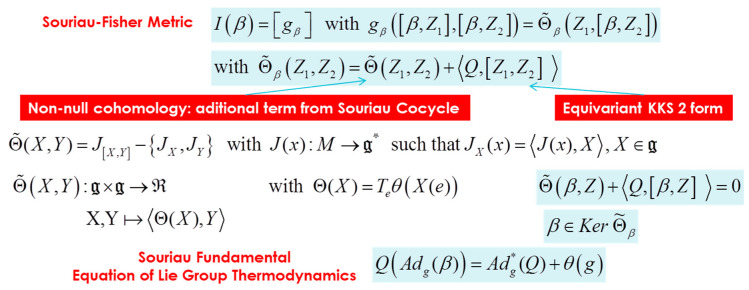
Souriau-Fisher metric as extension of KKS 2-form in case of non-null Cohomogy.

We observe that Souriau Riemannian metric, introduced with symplectic cocycle, is a generalization of the Fisher metric, that we call the Souriau-Fisher metric, that preserves the property to be defined as a Hessian of the partition function logarithm $g_{\beta }=-\frac{\partial^{2}\Phi }{\partial \beta^{2}}=\frac{\partial^{2}\log\psi_{\Omega }}{\partial \beta^{2}}$ as in classical information geometry. We will establish the equality of two terms, between Souriau definition based on Lie group cocycle Θ and parameterized by “geometric heat” *Q* (element of the dual space of the Lie algebra) and “geometric temperature” *β* (element of Lie algebra) and hessian of characteristic function $\Phi \left(\beta \right)=-\log\psi_{\Omega }(\beta )$ with respect to the variable *β* (as illustrated in Figure 5):

(49) $$g_{\beta }\left(\left[\beta ,Z_{1}\right],\left[\beta ,Z_{2}\right]\right)=\langle \Theta \left(Z_{1}\right),\left[\beta ,Z_{2}\right]\rangle +\langle Q,\left[Z_{1},\left[\beta ,Z_{2}\right]\right]\rangle =\frac{\partial^{2}\log\psi_{\Omega }}{\partial \beta^{2}}$$

If we differentiate this relation of Souriau theorem $Q\left(Ad_{g}(\beta )\right)=Ad_{g}^{*}(Q)+\theta \left(g\right)$, this relation occurs:

(50) $$\frac{\partial Q}{\partial \beta }\left(-\left[Z_{1},\beta \right],.\right)=\tilde{\Theta }\left(Z_{1},\left[\beta ,.\right]\right)+\langle Q,Ad_{.Z_{1}}(\left[\beta ,.\right])\rangle ={\tilde{\Theta }}_{\beta }\left(Z_{1},\left[\beta ,.\right]\right)$$

(51) $$-\frac{\partial Q}{\partial \beta }\left(\left[Z_{1},\beta \right],Z_{2}.\right)=\tilde{\Theta }\left(Z_{1},\left[\beta ,Z_{2}\right]\right)+\langle Q,Ad_{.Z_{1}}(\left[\beta ,Z_{2}\right])\rangle ={\tilde{\Theta }}_{\beta }\left(Z_{1},\left[\beta ,Z_{2}\right]\right)$$

(52) $$\Rightarrow -\frac{\partial Q}{\partial \beta }=g_{\beta }\left(\left[\beta ,Z_{1}\right],\left[\beta ,Z_{2}\right]\right)$$

As the entropy is defined by the Legendre transform of the characteristic function, a dual metric of the Fisher metric is also given by the hessian of “geometric entropy” S(Q) with respect to the dual variable given by *Q:* $\frac{\partial^{2}S(Q)}{\partial Q^{2}}$.

For the maximum entropy density (Gibbs density), the following three terms coincide: $\frac{\partial^{2}\log\psi_{\Omega }}{\partial \beta^{2}}$ that describes the convexity of the log-likelihood function, $I(\beta )=-E\left[\frac{\partial^{2}\log p_{\beta }(\xi )}{\partial \beta^{2}}\right]$ the Fisher metric that describes the covariance of the log-likelihood gradient, whereas $I(\beta )=E\left[\left(\xi -Q\right){\left(\xi -Q\right)}^{T}\right]=Var(\xi )$ that describes the covariance of the observables. We can also observe that the Fisher metric $I(\beta )=-\frac{\partial Q}{\partial \beta }$ is exactly the Souriau metric defined through symplectic cocycle:

(53) $$I(\beta )={\tilde{\Theta }}_{\beta }\left(Z_{1},\left[\beta ,Z_{2}\right]\right)=g_{\beta }\left(\left[\beta ,Z_{1}\right],\left[\beta ,Z_{2}\right]\right)$$

The Fisher metric $I(\beta )=-\frac{\partial^{2}\Phi (\beta )}{\partial \beta^{2}}=-\frac{\partial Q}{\partial \beta }$ has been considered by Souriau as a *generalization of* “*heat capacity*”. Souriau called it K the “*geometric capacity*”.

### 3.4. Covariant Souriau Gibbs Density and Information Manifold Foliation

R.F. Streater has studied in 1999, Information Geometry for some Lie algebra where for certain unitary representation of a Lie algebra, he has defined the statistical manifold of states as convex cone for which the partition function is finite, making reference to Bogoliubov-Kubo-Mori metric. But Streater has only developed the case with null cohomology for so (3) and sl (2,R) Lie alebras. Nevertheless, as observed by R.F. Streater in his paper “**Information Geometry for some Lie algebras**” [35], referring to Kirillov work and Roger Balian paper, “We can expect further natural structures to arise in this case. Indeed, it is known (*) that ***the dual to the Lie algebra****, which parametrizes the state-space in this case, **foliates into coadjoint orbits; there are also the level sets on the entropy**; **Kirillov form, and the BKM (Bogoliubov-Kubo-Mori) metric, together make each orbit into kähler space**, along the lines proposed by Kostant. **Motion along these holomorphic directions is nondissipative. The transversal to the orbits is a real half-line, which represents the dissipative direction**…We study the case of sl (2,R) in the discrete series of representations. We show **the information manifold foliates into level sets of the entropy**, each being isometric to H, the Poincaré upper half-plane… The states of constant entropy are the hyperboloids and* β *is the dissipative coordinate… For an integrable system described by a Lie algebra in a traceable representation, we find that **the information manifold foliates into complex spaces; the level sets of entropy can be given a complex structure by the method of Kostant**. Motion remaining on the complex surfaces is nondissipative, whereas motion transversal to these surfaces is dissipative. In information geometry, the state is parametrized by the canonical coordinates. Which function of them is measured by a thermometer? In our models, it is reasonable to designate* 1/β *to be the temperature; it is a dissipative coordinate, and it increases with time, showing that the system is thermalizing*”.

## 4. Mathematical Definition of Souriau Moment Map

Previously, we have introduced the concept of Souriau’s moment map. In this chapter, we will introduce a mathematical definition of this tool, as defined in Souriau’s book [36] with modern notations [37,38,39,40,41]. Other details on moment map are also given in Jean-Louis Koszul’s Book [42].

### 4.1. Operations on Vector Fields

Consider a map $F:X\subset R^{M}\to Y\subset R^{N}$, y=F(x), the derivative of F at x∈X, $DF:X\to R^{N\times M}$ is given by:

(54) $$\left(\begin{array}{c} \delta y^{1}\\ \vdots \\ \delta y^{N} \end{array}\right)=\left(\begin{array}{ccc} \frac{\partial y^{1}}{\partial x^{1}} & \cdots & \frac{\partial y^{1}}{\partial x^{M}}\\ \vdots & \ddots & \vdots \\ \frac{\partial y^{N}}{\partial x^{1}} & \cdots & \frac{\partial y^{N}}{\partial x^{M}} \end{array}\right)\left(\begin{array}{c} \delta x^{1}\\ \vdots \\ \delta x^{M} \end{array}\right)=DF(x)(\delta x)=\underset{t\to 0}{Lim}\frac{F(x+t\delta x)-F(x)}{t}$$

Second derivative is given by the linear map $D^{2}F:X\to R^{N\times M\times M}$:

(55) $$\delta \left[\frac{\partial y}{\partial x}\right]=\frac{\partial^{2}y}{\partial x^{2}}(\delta x)=D^{2}F(x)(\delta x)$$

Consider a vector Field V on $X\subset R^{M}$ defined by: $V:X\subset R^{M}\to R^{M}$, operations on vector fields are given by adjoint action and Lie bracket:

(56) $$Ad_{F}V(y)={\frac{d}{dt}\left[F\circ e^{tV}\circ F^{-1}\right](y)|}_{t=0}=DF(x)\left(V(x)\right)\;\mathrm{with}\;x=F^{-1}(y)$$

(57) $$\left[U,V\right](x)={\frac{d}{ds}Ad_{e^{sU}}V(x)|}_{s=0}=DU(x)\left(V(x)\right)-DV(x)\left(U(x)\right)$$

0-form is a scalar, 1-form are row $\omega =\left(\omega_{1}\cdots \omega_{M}\right)$ in dual space. 2-forms can be regarded as antisymmetric matrices $\left(\omega_{ij}\right)$ with $\omega \left(u,v\right)=u^{t}\left(\begin{array}{ccc} \omega_{11} & \cdots & \omega_{1M}\\ \vdots & \ddots & \vdots \\ \omega_{M1} & \cdots & \omega_{MM} \end{array}\right)v$. m-forms are all scalar multiples of the standard volume form vol, defined by $Vol\left(v_{1},\cdots ,v_{m}\right)=\det\left(\mathrm{matrix}\;\mathrm{with}\;\mathrm{columns}\;v_{1},\ldots ,v_{m}\right)$.

### 4.2. Derivative Rules by Sophus Lie, Elie Cartan and Henri Cartan

With the following classical definitions:

- ***Pull back***: $F^{*}\omega$ is a p-form on X
  (58) $$F^{*}\omega \left(v_{1},\cdots ,v_{p}\right)=\omega_{F(x)}\left(DF(x)(v_{1}),\cdots ,DF(x)(v_{p})\right)$$
- ***Interior product***: $i_{V}\omega$ is the (p−1)form on M obtained by inserting V(x) as the first argument of ω
  (59) $$i_{V}\omega \left(v_{2},\cdots v_{p}\right)=\omega \left(V(x),v_{2},\cdots ,v_{p}\right)$$
- ***Exterior product:***θ∧ω is the (p + 1)-form on X where ω is a p-form and θ is a 1-form on M (where the hat indicates a term to be omitted):
  (60) $$\theta \wedge \omega \left(v_{0},\cdots ,v_{p}\right)=\sum_{i=0}^{p}{\left(-1\right)}^{i}\theta (v_{i})\omega \left(v_{0},\cdots ,{\hat{v}}_{i},\cdots ,v_{p}\right)$$
- ***Lie derivative***: $L_{V}\omega$ is a p-form on M, and $L_{V}\omega =0$ if the flow of V consists of symmetries of ω:
  (61) $$L_{V}\omega \left(v_{1},\cdots ,v_{p}\right)={\frac{d}{dt}e^{tV*}\omega \left(v_{1},\cdots ,v_{p}\right)|}_{t=0}$$
  dω is the (p+1)-form on M defined by taking the ordinary derivative of ω and then antisymmetrizing:
- ***Exterior derivative:***
  (62) $$d\omega \left(v_{0},\cdots ,v_{p}\right)=\sum_{i=0}^{p}{\left(-1\right)}^{i}\frac{\partial \omega }{\partial x}(v_{i})(v_{0},\cdots ,{\hat{v}}_{i},\cdots ,v_{p})$$
  (63) $$p=0,{\left[d\omega \right]}_{i}=\partial_{i}\omega \;;\;p=1,{\left[d\omega \right]}_{ij}=\partial_{i}\omega_{j}-\partial_{j}\omega_{i}\;;\;p=2,{\left[d\omega \right]}_{ijk}=\partial_{i}\omega_{jk}+\partial_{j}\omega_{ki}+\partial_{k}\omega_{ij}$$

From these definitions, the properties of the exterior and Lie Derivative were established by Sophus Lie, Elie Cartan, and Henri Cartan:

(64) $$L_{V}\omega =di_{V}\omega +i_{V}d\omega$$

***(Elie Cartan equation)***

(65) $$i_{\left[U,V\right]}\omega =i_{V}L_{U}\omega -L_{U}i_{V}\omega$$

***(Henri Cartan equation)***

(66) $$L_{\left[U,V\right]}\omega =L_{V}L_{U}\omega -L_{U}L_{V}\omega$$

***(Sophus Lie equation)***

### 4.3. Souriau Moment Map

Considering Manifolds and Lie groups, We define the tangent bundle TX of X as the disjoint union of the $T_{x}X$, or the set of all pairs $\left(\begin{array}{c} \delta x\\ x \end{array}\right)$ with x∈X and $\delta x\in T_{x}X$. If F:X→Y is a smooth map between manifolds, its tangent map is the map:

(67) $$F_{*}\left(\begin{array}{c} \delta x\\ x \end{array}\right)=\left(\begin{array}{c} DF(x)(\delta x)\\ F(x) \end{array}\right)$$

A Lie group is a group G with a manifold structure such that the product (g,h)↦gh and the inversion $g\mapsto g^{-1}$ are smooth maps from G×G (resp. G) to G. Its Lie algebra is the tangent space $g=T_{e}G$ at the identity element. A smooth action of G on a manifold X is a group morphism:

(68) $$\begin{array}{l} \Phi :G\times X\to Diff(X)\\ \;\left(g,x\right)\mapsto g.x \end{array}$$

The orbit of x∈X is G(x)={g.x:g∈G}.

The tangent space to an orbit at x:

$T_{x}G(x)=\left\{Z(x):Z\in g\right\}=g/g_{x}$ with $Z(x)={\frac{d}{dt}e^{tZ}(x)|}_{t=0}$ and where

(69) $$g_{x}=\left\{Z\in g:Z(x)=0\right\}$$

Let (M,σ) be a connected symplectic manifold. A vector field η on M is called symplectic if its flow preserves the 2-form: $L_{\eta }\sigma =0$. If we use Elie Cartan’s formula, we can deduce that $L_{\eta }\sigma =di_{\eta }\sigma +i_{\eta }d\sigma =0$ but as dσ=0 then $di_{\eta }\sigma =0$. We observe that the 1-form $i_{\eta }\sigma$ is closed. When this 1-form is exact, there is a smooth function x↦H on M with:

(70) $$i_{\eta }\sigma =-dH$$

This vector field η is called Hamiltonian and could be defined as symplectic gradient $\eta =\nabla_{Symp}H$.

Let a Lie group G that acts on M and that also preserve σ. A moment map exists if these infinitesimal generators are actually hamiltonian, so that a map $J:M\to g^{*}$ exists with:

$i_{Z_{X}}\sigma =-dH_{Z}$ where

(71) $$H_{Z}=\langle J(x),Z\rangle$$

The Poisson bracket of two functions H, $H^{\prime }$ is defined by:

(72) $$\begin{array}{l} \left\{H,H^{\prime }\right\}=\sigma \left(\eta ,\eta^{\prime }\right)=\sigma \left(\nabla_{Symp}H^{\prime },\nabla_{Symp}H\right)\;\mathrm{with}\;i_{\eta }\sigma =-dH\;\mathrm{and}\\ \;i_{\eta^{\prime }}\sigma =-dH^{\prime } \end{array}$$

If G is connected, then the moment map is G-equivariant if and only if it satisfies $\left\{H_{Z},H_{Z^{\prime }}\right\}=H_{\left[Z,Z^{\prime }\right]}$.

Souriau has proved thet every coadjoint orbit of a Lie group is a homogeneous symplectic manifold when endowed with the KKS 2-form $\sigma \left(Z(x),Z^{\prime }(x)\right)=\langle x,\left[Z^{\prime },Z\right]\rangle$, and conversely, every homogeneous symplectic manifold of a connected Lie group *G* is, up to a possible covering, a coadjoint orbit of some central extension of *G*. σ is G-invariant.

## 5. Poincaré Unit Disk, SU(1,1) Lie Group and Souriau Moment Map

We will introduce Souriau moment map for SU(1,1)/K group that acts transitively on Poincaré Unit Disk, based on moment map. More details on computation of moment map for SU(1,1)/K Lie group is given in Appendix A of this document.

### 5.1. Poincaré Unit Disk and SU(1,1) Lie Group

The group of complex unimodular pseudo-unitary matrices SU(1,1), is the set of elements u such that [43,44,45,46,47,48,49,50,51,52]:

(73) $$uMu^{+}=M\;\mathrm{with}\;M=\left(\begin{array}{cc} +1 & 0\\ 0 & -1 \end{array}\right)$$

We can show that the most general matrix u belongs to the Lie group given by:

(74) $$G=SU(1,1)=\left\{\left(\begin{array}{cc} a & b\\ b^{*} & a^{*} \end{array}\right)/{\left|a\right|}^{2}-{\left|b\right|}^{2}=1,\;a,b\in C\right\}$$

Its Cartan decomposition is given by:

(75) $$\left(\begin{array}{cc} a & b\\ b^{*} & a^{*} \end{array}\right)=\left|a\right|\left(\begin{array}{cc} 1 & z\\ z^{*} & 1 \end{array}\right)\left(\begin{array}{cc} a/\left|a\right| & 0\\ 0 & a^{*}/\left|a\right| \end{array}\right)\;\mathrm{with}\;z=b{\left(a^{*}\right)}^{-1},\left|a\right|={\left(1-{\left|z\right|}^{2}\right)}^{-1/2}$$

(76) $$\left(\begin{array}{cc} a & b\\ b^{*} & a^{*} \end{array}\right)\left(\begin{array}{cc} 1 & z\\ z^{*} & 1 \end{array}\right)=\left|a^{\prime }\right|\left(\begin{array}{cc} 1 & z^{\prime }\\ {z^{\prime }}^{*} & 1 \end{array}\right)\left(\begin{array}{cc} a^{\prime }/\left|a^{\prime }\right| & 0\\ 0 & {a^{\prime }}^{*}/\left|a^{\prime }\right| \end{array}\right)\;\mathrm{with}\;\left\{ \begin{array}{l} a^{\prime }=bz^{*}+a\\ z^{\prime }=\frac{az+b}{b^{*}z+a^{*}} \end{array}\right.$$

SU(1,1) is associated to group of holomorphic automorphisms of the Poincaré unit disk D={z=x+iy∈C/|z|<1} in the complex plane, by considering its action on the disk as $g(z)=\left(az+b\right)/\left(b^{*}z+a^{*}\right)$. The following measure on Unit disk:

(77) $$d\mu_{0}\left(z,z^{*}\right)=\frac{1}{2\pi i}\frac{dz\wedge dz^{*}}{{\left(1-{\left|z\right|}^{2}\right)}^{2}}$$

is invariant under the action of SU(1,1) captured by the fractional holomorphic transformation:

(78) $$\frac{dz^{\prime }\wedge d{z^{\prime }}^{*}}{{\left(1-{\left|z^{\prime }\right|}^{2}\right)}^{2}}=\frac{dz\wedge dz^{*}}{{\left(1-{\left|z\right|}^{2}\right)}^{2}}$$

The complex unit disk admits a Kähler structure determined by potential function:

(79) $$\Phi \left(z^{\prime },z^{*}\right)=-\log\left(1-z^{\prime }z^{*}\right)$$

The invariant 2-form is:

(80) $$\Omega =\frac{1}{i}\frac{\partial^{2}\Phi \left(z,z^{*}\right)}{\partial z\partial z^{*}}dz\wedge dz^{*}=\frac{1}{i}\frac{dz\wedge dz^{*}}{{\left(1-{\left|z\right|}^{2}\right)}^{2}}$$

which is closed dΩ=0. This group SU(1,1) is isomorphic to the group SL(2,R) as a real Lie group, and the Lie algebra g=𝖘u(1,1) is given by:

(81) $$g=\left\{\left(\begin{array}{cc} -ir & \eta \\ \eta^{*} & ir \end{array}\right)/r\in R,\eta \in C\right\}$$

with the bases $\left(u_{1},u_{2},u_{3}\right)\in g$: $u_{1}=\frac{1}{2}\left(\begin{array}{cc} 0 & -i\\ i & 0 \end{array}\right)\;,\;u_{2}=\frac{1}{2}\left(\begin{array}{cc} 0 & 1\\ 1 & 0 \end{array}\right)\;,\;u_{3}=\frac{1}{2}\left(\begin{array}{cc} -i & 0\\ 0 & i \end{array}\right)$

with the commutation relation:

(82) $$\left[u_{3},u_{2}\right]=u_{1},\left[u_{3},u_{1}\right]=-u_{2},\left[u_{2},u_{1}\right]=-u_{3}$$

Dual base on the dual space of the Lie algebra is named $\left(u_{1}^{*},u_{2}^{*},u_{3}^{*}\right)\in g^{*}$. The dual vector space $g^{*}=𝖘u^{*}(1,1)$ can be identified with the subspace of 𝖘𝖑(2,C) of the form:

(83) $$g^{*}=\left\{\left(\begin{array}{cc} z & x+iy\\ -x+iy & -z \end{array}\right)=x\left(\begin{array}{cc} 0 & 1\\ -1 & 0 \end{array}\right)+y\left(\begin{array}{cc} 0 & i\\ i & 0 \end{array}\right)+z\left(\begin{array}{cc} 1 & 0\\ 0 & -1 \end{array}\right)/x,y,z\in R\right\}$$

Coadjoint action of g∈G on dual space of the Lie algebra $\xi \in g^{*}$ is written g.ξ.

### 5.2. Coadjoint Orbit of SU(1,1) and Souriau Moment Map

We will use results of C. Cishahayo and S. de Bièvre [53] and B. Cahen [54,55] for computation of moment map of SU(1,1). Let $r\in R^{*+}$, orbit $O\left(ru_{3}^{*}\right)$ of $ru_{3}^{*}$ for the coadjoint action of g∈G could be identified with the upper half sheet $x_{3}>0$ of $\left\{\xi =x_{1}u_{1}^{*}+x_{2}u_{2}^{*}+x_{3}u_{3}^{*}/-x_{1}^{2}-x_{2}^{2}+x_{3}^{2}=r^{2}\right\}$, the two-sheet hyperboloid. The stabilizer of $ru_{3}^{*}$ for the coadjoint action of G is torus $K=\left\{\left(\begin{array}{cc} e^{i\theta } & 0\\ 0 & e^{-i\theta } \end{array}\right),\theta \in R\right\}$. *K* induces rotations of the unit disk, and leaves 0 invariant. The stabilizer for the origin 0 of unit disk is maximal compact subgroup *K* of *SU(1,1)*. We can observe [54] that $O\left(ru_{3}^{*}\right)=G/K$. On the other hand $O\left(ru_{3}^{*}\right)=G/K$ is diffeomorphic to the unit disk D={z∈C/|z|<1}, then by composition, the Souriau moment map is given by:

(84) $$\begin{array}{l} J:D\to O\left(ru_{3}^{*}\right)\\ \;z\mapsto J(z)=r\left(\frac{z+z^{*}}{\left(1-{\left|z\right|}^{2}\right)}u_{1}^{*}+\frac{z-z^{*}}{i\left(1-{\left|z\right|}^{2}\right)}u_{2}^{*}+\frac{1+{\left|z\right|}^{2}}{\left(1-{\left|z\right|}^{2}\right)}u_{3}^{*}\right) \end{array}$$

J is linked to the natural action of G on D (by fractional linear transforms) but also the coadjoint action of G on $O\left(ru_{3}^{*}\right)=G/K$. $J^{-1}$ could be interpreted as the stereographic projection from the two-sphere $S^{2}$ onto C∪∞ [56]. In case $r=\frac{n}{2}$ where $n\in N^{+},n\geq 2$ then the coadjoint orbit is given by $O_{n}=O\left(\zeta_{n}\right)$ with $\xi_{n}=\frac{n}{2}u_{3}^{*}\in g^{*}$, with stabilizer of $\xi_{n}$ for coadjoint action the torus $K=\left\{\left(\begin{array}{cc} e^{i\theta } & 0\\ 0 & e^{-i\theta } \end{array}\right),\theta \in R\right\}$ with Lie algebra $Ru_{3}$. $O_{n}=O\left(\zeta_{n}\right)$ is associated with a holomorphic discrete series representation $\pi_{n}$ of G by the KKS (Kirillov-Kostant-Souriau) method of orbits.

(85) $$\begin{array}{l} J:D\to O_{n}\\ \;z\mapsto J(z)=\frac{n}{2}\left(\frac{z+z^{*}}{\left(1-{\left|z\right|}^{2}\right)}u_{1}^{*}+\frac{z-z^{*}}{i\left(1-{\left|z\right|}^{2}\right)}u_{2}^{*}+\frac{1+{\left|z\right|}^{2}}{\left(1-{\left|z\right|}^{2}\right)}u_{3}^{*}\right) \end{array}$$

Group G act on D by homography $g.z=\left(\begin{array}{cc} a & b\\ b^{*} & a^{*} \end{array}\right).z=\frac{az+b}{b^{*}z+a^{*}}$. ***This action corresponds with coadjoint action of*** G ***on*** $O_{n}$. The Kirillov-Kostant-Souriau 2-form of $O_{n}$ is given by:

(86) $$\Omega_{n}\left(\zeta \right)\left(X\left(\zeta \right),Y\left(\zeta \right)\right)=\langle \zeta ,\left[X,Y\right]\rangle \;,\;X,Y\in g\;\mathrm{and}\;\zeta \in O_{n}$$

and is associated in the frame by J with:

(87) $$\omega_{n}=\frac{in}{{\left(1-{\left|z\right|}^{2}\right)}^{2}}dz\wedge dz^{*}$$

with the corresponding Poisson Bracket:

(88) $$\left\{f,g\right\}=i{\left(1-{\left|z\right|}^{2}\right)}^{2}\left(\frac{\partial f}{\partial z}\frac{\partial g}{\partial z^{*}}-\frac{\partial f}{\partial z^{*}}\frac{\partial g}{\partial z}\right)$$

It has been also observed that there are 3 basic observables generating the SU(1,1) symmetry on classical level:

(89) $$\left\{ \begin{array}{l} D\to R\\ z\mapsto k_{3}(z)=\frac{1+{\left|z\right|}^{2}}{1-{\left|z\right|}^{2}} \end{array}\right.,\left\{ \begin{array}{l} D\to R\\ z\mapsto k_{1}(z)=\frac{1}{i}\frac{z-z^{*}}{1-{\left|z\right|}^{2}} \end{array}\right.,\left\{ \begin{array}{l} D\to R\\ z\mapsto k_{2}(z)=\frac{z+z^{*}}{1-{\left|z\right|}^{2}} \end{array}\right.$$

with the Poisson commutation rule:

(90) $$\left\{k_{3},k_{1}\right\}=k_{2},\left\{k_{3},k_{2}\right\}=-k_{1},\left\{k_{1},k_{2}\right\}=-k_{3}$$

$\left(k_{1},k_{2},k_{3}\right)$ vector points to the upper sheet of the two-sheeted hyperboloid in $R^{3}$ given by $k_{3}^{2}-k_{1}^{2}-k_{2}^{2}=1$, whose the stereographic projection onto the open unit disk is:

(91) $$\left\{ \begin{array}{l} \left(k_{1},k_{2},k_{3}\right)\in H^{+}\to D\\ z=\frac{k_{2}+ik_{1}}{1+k_{3}}=\sqrt{\frac{k_{3}-1}{k_{3}+1}}e^{i\arg z} \end{array}\right.$$

Under the action of $g\in G=SU(1,1)=\left\{\left(\begin{array}{cc} a & b\\ b^{*} & a^{*} \end{array}\right)/{\left|a\right|}^{2}-{\left|b\right|}^{2}=1,\;a,b\in C\right\}$:

$\left(\begin{array}{cc} k_{-} & k_{3}\\ k_{3} & k_{+} \end{array}\right)=\left(\begin{array}{cc} k_{2}+ik_{1} & k_{3}\\ k_{3} & k_{2}-ik_{1} \end{array}\right)=\frac{1}{1-{\left|z\right|}^{2}}\left(\begin{array}{cc} 2z & 1+{\left|z\right|}^{2}\\ 1+{\left|z\right|}^{2} & 2z^{*} \end{array}\right)$ is transform in:

(92) $$\left(\begin{array}{cc} k_{-}^{\prime } & k_{3}^{\prime }\\ k_{3}^{\prime } & k_{+}^{\prime } \end{array}\right)=\left(\begin{array}{cc} k_{-}\left(g^{-1}.z\right) & k_{3}\left(g^{-1}.z\right)\\ k_{3}\left(g^{-1}.z\right) & k_{+}\left(g^{-1}.z\right) \end{array}\right)=g^{-1}\left(\begin{array}{cc} k_{-} & k_{3}\\ k_{3} & k_{+} \end{array}\right){\left(g^{-1}\right)}^{t}$$

***This transform can be viewed as the co-adjoint action of*** SU(1,1) ***on the coadjoint orbit identified with*** $k_{3}^{2}-k_{1}^{2}-k_{2}^{2}=1$. We can also observe that the quotient SU(1,1)/K is isomorphic to the upper sheet of the hyperboloid described by $k_{3}^{2}-k_{1}^{2}-k_{2}^{2}=1$, by the following parametrization (τ,φ), given by $\vec{n}=\left(\cosh\tau ,\sinh\tau \cos\varphi ,\sinh\tau \sin\varphi \right)$, and its stereographic projection onto the inside of the unit disk, parametrized by $\varsigma =\tanh\frac{\tau }{2}e^{-i\varphi }$.

## 6. Covariant Gibbs Density by Souriau Thermodynamics for Poincaré Unit Disk

### 6.1. Fourier Transform, Laplace Transform and Lie Group Representation Theory

In Souriau Lie Group Thermododynamic, we have to consider Laplace Transform defined on coadjoint orbits to define Massieu Potential Function and Gibbs density. This problem has been solved in the domain of Kirillov Representation Theory. Representation theory studies abstract algebraic structures by representing their elements as linear transformations of vector spaces, and algebraic objects (Lie groups, Lie algebras) by describing its elements by matrices and the algebraic operations in terms of matrix addition and matrix multiplication, reducing problems of abstract algebra to problems in linear algebra. Representation theory generalizes Fourier analysis via harmonic analysis. The modern development of Fourier analysis during XXth century has explored the generalization of Fourier and Fourier-Plancherel formula for non-commutative harmonic analysis, applied to locally compact non-Abelian groups. This has been solved by geometric approaches based on “orbits methods” (Fourier-Plancherel formula for *G* is given by coadjoint representation of *G* in dual vector space of its Lie algebra) with many contributors (Dixmier, Kirillov, Bernat, Arnold, Berezin, Kostant, Souriau, Duflo, Guichardet, Torasso, Vergne, Paradan, etc.) [57,58,59,60,61,62,63,64,65,66,67,68].

For classical commutative harmonic analysis, we consider the following groups:

(93) $$\begin{array}{c} G={Τ}^{n}=R^{n}/Z^{n}\;\mathrm{for}\;\mathrm{Fourier}\;\mathrm{series},\;G=R^{n}\;\mathrm{for}\;\mathrm{Fourier}\;\mathrm{Transform}\\ G\;\mathrm{group}\;\mathrm{character}\;(\mathrm{linked}\;\mathrm{to}\;e^{ikx}):\chi :G\to U\;\mathrm{with}\;U=\left\{z\in C/\left|z\right|=1\right\}\\ \hat{G}=\left\{\chi /\chi_{1}.\chi_{2}(g)=\chi_{1}(g)\chi_{2}(g)\right\}\;\mathrm{and}\;\mathrm{Fourier}\;\mathrm{transform}\;\mathrm{is}\;\mathrm{given}\;\mathrm{by}:\\ \;\varphi :G\to C\;\hat{\varphi }:\hat{G}\to C\\ \;g\mapsto \varphi \left(g\right)=\int_{\hat{G}}\hat{\varphi }(\chi )\chi {\left(g\right)}^{-1}d\chi \;\chi \mapsto \hat{\varphi }\left(\chi \right)=\int_{G}\varphi (g)\chi (g)dg \end{array}$$

For non-commutative harmonic analysis, Group unitary irreductible representation is U:G→U(H) with H Hilbert space and character by $\chi_{U}(g)=trU_{g}$. Fourier transform for non-commutative group is $U_{\varphi }=\int_{G}\varphi (g)U_{g}dg$ with character $\chi_{U}(g)=trU_{\varphi }$. If we describe group element with exponential map $U_{\psi }=\int_{g}\psi (X)U_{\exp(X)}dX$, we have:

(94) $$\begin{array}{l} {\mathrm{trU}}_{\psi }=\dim\tau .\mu_{G.f}\left(\overset{\wedge }{\psi .j^{-1}}\right)\\ \overset{\wedge }{\psi .j^{-1}}:g\to g^{*},\;\mathrm{Four}.\;\mathrm{Transf}. \end{array}\;\mathrm{with}\;\left\{ \begin{array}{l} \mu_{G.f}:\;\mathrm{Liouville}\;\mathrm{meas}.\;\mathrm{on}\;O=G.f,f\in g^{*}\\ \mu_{G.f}\left(\overset{\wedge }{\psi .j^{-1}}\right):\;\mathrm{Integral}\;\mathrm{of}\;\overset{\wedge }{\psi .j^{-1}}\mathrm{wrt}\;\mu_{G.f}\; \end{array}\right.$$

where

(95) $$j\left(X\right)={\left(\det s\left(ad_{X}\right)\right)}^{1/2}\;\mathrm{with}\;s(x)=\sum_{n=0}^{\infty }\frac{1}{(2n+1)!}{\left(\frac{x}{2}\right)}^{2n}=sh\left(\frac{x}{2}\right)/\left(\frac{x}{2}\right)$$

Kirillov Character formula is:

(96) $$\chi_{U}\left(\exp(X)\right)={\mathrm{trU}}_{\exp(X)}=j{\left(X\right)}^{-1}\int_{O}e^{i\langle f,X\rangle }d\mu_{O}(f)$$

(97) $$\int_{O}e^{i\langle f,X\rangle }d\mu_{O}(f)=j(X){\mathrm{trU}}_{\exp(X)}\;\mathrm{with}\;j\left(X\right)={\left(\det\left(\frac{e^{ad_{X}/2}-e^{-ad_{X}/2}}{ad_{X}/2}\right)\right)}^{1/2}\;$$

We will use Kirillov representation theory and his character formula to compute Souriau covariant Gibbs density in the unit Poincaré disk. For any Lie group G, a coadjoint orbit $O\subset g^{*}$ has a canonical symplectic form $\omega_{0}$ given by KKS 2-form. As seen, if G is finite dimensional, the corresponding volume element defines a G-invariant measure supported on O, which can be interpreted as a tempered distribution. The Fourier transform (where *d* is the half of the dimension of the orbit O):

(98) $$ℑ(x)=\int_{O\subset g^{*}}e^{-i\langle \lambda ,x\rangle }\frac{1}{d!}d\omega_{O^{d}}\;\mathrm{with}\;\lambda \in g^{*}\;\mathrm{and}\;x\in g$$

is Ad G-invariant. When $O\subset g^{*}$ is an integral coadjoint orbit, Kirillov formula, given previously, expresses Fourier transform ℑ(x) by Kirillov character $\chi_{O}$:

(99) $$ℑ(x)=j(x)\chi_{O}\left(e^{x}\right)\;\mathrm{where}\;j(x)=\det^{1/2}\left(\frac{\sinh\left(ad\left(x/2\right)\right)}{ad\left(x/2\right)}\right)$$

$\chi_{O}$ is, as defined previously, the “*Kirillov character*” of a unitary representation associated to the orbit.

### 6.2. Souriau Covariant Gibbs Density in Poincaré Unit Disk for SU(1,1) Lie Group

In the following, we will give the full development to compute the Souriau covariant Gibbs density. As the Gibbs density is not defined for all geometric temperature, as observed by Souriau, we have used his approach by considering a one-parameter subgroup of the Lie group generated by exponential map from a one element of Lie algebra given by geometric temperature. The subset of Lie algebra where the Gibbs density is deduced from the contraints related to this one-parameter subgroup generation.

Considering the Lie group $SU(1,1)=\left\{\left(\begin{array}{cc} a & b\\ b^{*} & a^{*} \end{array}\right)/a,b\in C,\;{\left|a\right|}^{2}-{\left|b\right|}^{2}=1\right\}$ and its Lie algebra given by elements $su(1,1)=\left\{\left(\begin{array}{cc} ir & \eta \\ \eta^{*} & -ir \end{array}\right)/r\in R,\eta \in C\right\}$. A basis for this Lie algebra su(1,1) is $\left(u_{1},u_{2},u_{3}\right)\in g$ with $u_{1}=\frac{i}{2}\left(\begin{array}{cc} 1 & 0\\ 0 & -1 \end{array}\right),u_{2}=-\frac{1}{2}\left(\begin{array}{cc} 0 & 1\\ 1 & 0 \end{array}\right)\;\mathrm{and}\;u_{3}=\frac{1}{2}\left(\begin{array}{cc} 0 & -i\\ i & 0 \end{array}\right)$ with $\left[u_{1},u_{3}\right]=-u_{2},\left[u_{1},u_{2}\right]=u_{3},\left[u_{2},u_{3}\right]=-u_{1}$.

The compact subgroup is generated by $u_{1}$, while $u_{2}$ and $u_{3}$ generate a hyperbolic subgroup. The dual space of the Lie algebra is given by $su{\left(1,1\right)}^{*}=\left\{\left(\begin{array}{cc} z & x+iy\\ -x+iy & -z \end{array}\right)/x,y,z\in R\right\}$ with the basis $\left(u_{1}^{*},u_{2}^{*},u_{3}^{*}\right)\in g^{*}$ with $u_{1}^{*}=\left(\begin{array}{cc} 1 & 0\\ 0 & -1 \end{array}\right),u_{2}^{*}=\left(\begin{array}{cc} 0 & i\\ i & 0 \end{array}\right)\;\mathrm{and}\;u_{3}^{*}=\left(\begin{array}{cc} 0 & 1\\ -1 & 0 \end{array}\right)$.

Let consider D={z∈C/|z|<1} be the open unit disk of Poincaré. For each ρ>0, the pair $\left(D,\omega_{\rho }\right)$ is a symplectic homogeneous manifold with $\omega_{\rho }=2i\rho \frac{dz\wedge dz^{*}}{{\left(1-{\left|z\right|}^{2}\right)}^{2}}$, where $\omega_{\rho }$ is invariant under the action: $\begin{array}{l} SU\left(1,1\right)\times D\to D\\ \left(g,z\right)\mapsto g.z=\frac{az+b}{b^{*}z+a^{*}} \end{array}$.

This action is transitive and is globally and strongly Hamiltonian. Its generators are the hamiltonian vector fields associated to the functions:

(100) $$J_{1}(z,z^{*})=\rho \frac{1+{\left|z\right|}^{2}}{1-{\left|z\right|}^{2}}\;,\;J_{2}(z,z^{*})=\frac{\rho }{i}\frac{z-z^{*}}{1-{\left|z\right|}^{2}}\;,\;J_{3}(z,z^{*})=-\rho \frac{z+z^{*}}{1-{\left|z\right|}^{2}}$$

The associated moment map $J:D\to su^{*}(1,1)$ defined by $J(z).u_{i}=J_{i}(z,z^{*})$, maps D into a coadjoint orbit in $su^{*}(1,1)$. Then, we can write the moment map as a matrix element of $su^{*}(1,1)$:

(101) $$\begin{array}{l} J(z)=J_{1}\left(z,z^{*}\right)u_{1}^{*}+J_{2}\left(z,z^{*}\right)u_{2}^{*}+J_{3}\left(z,z^{*}\right)u_{3}^{*}=\left(\begin{array}{cc} \rho \frac{1+{\left|z\right|}^{2}}{1-{\left|z\right|}^{2}} & \rho \frac{z-z^{*}}{1-{\left|z\right|}^{2}}-\rho \frac{z+z^{*}}{1-{\left|z\right|}^{2}}\\ \rho \frac{z-z^{*}}{1-{\left|z\right|}^{2}}+\rho \frac{z+z^{*}}{1-{\left|z\right|}^{2}} & -\rho \frac{1+{\left|z\right|}^{2}}{1-{\left|z\right|}^{2}} \end{array}\right)\\ J(z)=J_{1}\left(z,z^{*}\right)u_{1}^{*}+J_{2}\left(z,z^{*}\right)u_{2}^{*}+J_{3}\left(z,z^{*}\right)u_{3}^{*}=\rho \left(\begin{array}{cc} \frac{1+{\left|z\right|}^{2}}{1-{\left|z\right|}^{2}} & -2\frac{z^{*}}{1-{\left|z\right|}^{2}}\\ 2\frac{z}{1-{\left|z\right|}^{2}} & -\frac{1+{\left|z\right|}^{2}}{1-{\left|z\right|}^{2}} \end{array}\right)\in g^{*} \end{array}$$

The moment map J is a diffeomorphism of D onto one sheet of the two-sheeted hyperboloid in $su^{*}(1,1)$, determined by $J_{1}^{2}-J_{2}^{2}-J_{3}^{2}=\rho^{2}\;,\;J_{1}\geq \rho \;\mathrm{with}\;J_{1}u_{1}^{*}+J_{2}u_{2}^{*}+J_{3}u_{3}^{*}\in su^{*}(1,1)$. We note $O_{\rho }^{+}$ the coadjoint orbit $Ad_{SU(1,1)}^{*}$ of SU(1,1), given by the upper sheet of the two-sheeted hyperboloid given by previous equation. The orbit method of Kostant-Kirillov-Souriau associates to each of these coadjoint orbits a representation of the discrete series of SU(1,1), provided that ρ is a half integer greater or equal than 1 ($\rho =\frac{k}{2},k\in N\;\mathrm{and}\;\rho \geq 1$). When explicitly executing the Kostant-Kirillov construction, the representation Hilbert spaces $H_{\rho }$ are realized as closed reproducing kernel subspaces of $L^{2}\left(D,\omega_{\rho }\right)$. The Kostant-Kirillov-Souriau orbit method shows that to each coadjoint orbit of a connected Lie group is associated a unitary irreducible representation of G acting in a Hilbert space *H*.

Souriau has oberved that action of the full Galilean group on the space of motions of an isolated mechanical system is not related to any equilibrium Gibbs state (the open subset of the Lie algebra, associated to this Gibbs state is empty). The main Souriau idea was to define the Gibbs states for one-parameter subgroups of the Galilean group. We will use the same approach, in this case We will consider action of the Lie group SU(1,1) on the symplectic manifold (*M*,*ω*) (Poincaré unit disk) and its momentum map J are such that the open subset $\Lambda_{\beta }=\left\{\beta \in g/\int_{D}e^{-\langle J(z),\beta \rangle }d\lambda (z)<+\infty \right\}\;$ is not empty. This condition is not always satisfied when (*M, ω*) is a cotangent bundle, but of course it is satisfied when it is a compact manifold. The idea of Souriau is to consider a one parameter subgroup of SU(1,1). To parametrize elements of SU(1,1) is through its Lie algebra. In the neighborhood of the identity element, the elements of g∈SU(1,1) can be written as the exponential of an element β of its Lie algebra:

(102) g=exp(εβ) with β∈g

The condition $g^{+}Mg=M\;\mathrm{for}\;M=\left(\begin{array}{cc} 1 & 0\\ 0 & -1 \end{array}\right)$ can be expanded for ε<<1 and is equivalent to $\beta^{+}M+M\beta =0$ which then implies $\beta =\left(\begin{array}{cc} ir & \eta \\ \eta^{*} & -ir \end{array}\right),r\in R,\eta \in C$. We can observe that r and $\eta =\eta_{R}+i\eta_{I}$ contain 3 degrees of freedom, as required. Also because detg=1, we get Tr(β)=0. We can then exponentiate β with exponential map to get:

(103) $$g=\exp\left(\varepsilon \beta \right)=\sum_{k=0}^{\infty }\frac{{\left(\varepsilon \beta \right)}^{k}}{k!}=\left(\begin{array}{cc} a_{\varepsilon }(\beta ) & b_{\varepsilon }(\beta )\\ b_{\varepsilon }^{*}(\beta ) & a_{\varepsilon }^{*}(\beta ) \end{array}\right)$$

If we make the remark that $\beta^{2}=\left(\begin{array}{cc} ir & \eta \\ \eta^{*} & -ir \end{array}\right)\left(\begin{array}{cc} ir & \eta \\ \eta^{*} & -ir \end{array}\right)=\left({\left|\eta \right|}^{2}-r^{2}\right)I$, we can developed the exponential map:

(104) $$g=\exp\left(\varepsilon \beta \right)=\left(\begin{array}{cc} \cosh\left(\varepsilon R\right)+ir\frac{\sinh\left(\varepsilon R\right)}{R} & \eta \frac{\sinh\left(\varepsilon R\right)}{R}\\ \eta^{*}\frac{\sinh\left(\varepsilon R\right)}{R} & \cosh\left(\varepsilon R\right)-ir\frac{\sinh\left(\varepsilon R\right)}{R} \end{array}\right)\;\mathrm{with}\;R^{2}={\left|\eta \right|}^{2}-r^{2}$$

We can observe that one condition is that ${\left|\eta \right|}^{2}-r^{2}>0$ then the subset to consider is $\Lambda_{\beta }=\left\{\beta =\left(\begin{array}{cc} ir & \eta \\ \eta^{*} & -ir \end{array}\right),r\in R,\eta \in C/{\left|\eta \right|}^{2}-r^{2}>0\right\}\;$ such that $\int_{D}e^{-\langle J(z),\beta \rangle }d\lambda (z)<+\infty$. The generalized Gibbs states of the full SU(1,1) group do not exist. However, generalized Gibbs states for the one-parameter subgroups exp(αβ), $\beta \in \Lambda_{\beta }$, of the SU(1,1) group do exist. The generalized Gibbs state associated to β remains invariant under the restriction of the action to the one-parameter subgroup of SU(1,1) generated by exp(εβ).

To go futher, we will develop the Souriau Gibbs density from the Souriau moment map J(z) and the Souriau temperature $\beta \in \Lambda_{\beta }\;$. If we note $b=\frac{1}{1-{\left|z\right|}^{2}}\left[\begin{array}{c} 1\\ -z \end{array}\right]$, we can write the moment map:

(105) $$J(z)=\rho \left(\begin{array}{cc} \frac{1+{\left|z\right|}^{2}}{1-{\left|z\right|}^{2}} & -2\frac{z^{*}}{1-{\left|z\right|}^{2}}\\ 2\frac{z}{1-{\left|z\right|}^{2}} & -\frac{1+{\left|z\right|}^{2}}{1-{\left|z\right|}^{2}} \end{array}\right)=\rho \left(2Mbb^{+}-Tr\left(Mbb^{+}\right)I\right)\;\mathrm{with}\;M=\left[\begin{array}{cc} 1 & 0\\ 0 & -1 \end{array}\right]$$

We can the write the covariant Gibbs density in the unit disk given by moment map of the Lie group SU(1,1) and geometric temperature in its Lie algebra $\beta \in \Lambda_{\beta }$:

(106) $$p_{Gibbs}\left(z\right)=\frac{e^{-\langle J\left(z\right),\beta \rangle }}{\int_{D}e^{-\langle J\left(z\right),\beta \rangle }d\lambda (z)}\;\mathrm{where}\;d\lambda (z)=2i\rho \frac{dz\wedge dz^{*}}{{\left(1-{\left|z\right|}^{2}\right)}^{2}}$$

(107) $$p_{Gibbs}\left(z\right)=\frac{e^{-\langle \rho \left(2ℑbb^{+}-Tr\left(ℑbb^{+}\right)I\right),\beta \rangle }}{\int_{D}e^{-\langle J\left(z\right),\beta \rangle }d\lambda (z)}=\frac{e^{-\langle \rho \left(\begin{array}{cc} \frac{1+{\left|z\right|}^{2}}{\left(1-{\left|z\right|}^{2}\right)} & \frac{-2z^{*}}{\left(1-{\left|z\right|}^{2}\right)}\\ \frac{2z}{\left(1-{\left|z\right|}^{2}\right)} & -\frac{1+{\left|z\right|}^{2}}{\left(1-{\left|z\right|}^{2}\right)} \end{array}\right),\left(\begin{array}{cc} ir & \eta \\ \eta^{*} & -ir \end{array}\right)\rangle }}{\int_{D}e^{-\langle J\left(z\right),\beta \rangle }d\lambda (z)}$$

To write the Gibbs density with respect to its statistical moments, we have to express the density with respect to Q=E[J(z)]. Then, we have to invert the relation between Q and β, to replace this last variable $\beta =\left(\begin{array}{cc} ir & \eta \\ \eta^{*} & -ir \end{array}\right)\in \Lambda_{\beta }$ by $\beta =\Theta^{-1}\left(Q\right)\in g$ where $Q=\frac{\partial \Phi \left(\beta \right)}{\partial \beta }=\Theta \left(\beta \right)\in g^{*}$ with $\Phi \left(\beta \right)=-\log\int_{D}e^{-\langle J\left(z\right),\beta \rangle }d\lambda (z)$, deduce from Legendre tranform. The mean moment map is given by:

(108) $$Q=E\left[J(z)\right]=E\left[\rho \left(\begin{array}{cc} \frac{1+{\left|w\right|}^{2}}{\left(1-{\left|w\right|}^{2}\right)} & \frac{-2w^{*}}{\left(1-{\left|w\right|}^{2}\right)}\\ \frac{2w}{\left(1-{\left|w\right|}^{2}\right)} & -\frac{1+{\left|w\right|}^{2}}{\left(1-{\left|w\right|}^{2}\right)} \end{array}\right)\right]\;\mathrm{where}\;w\in D$$

This mean moment map can be obtained by Karcher mean computation on the one-sheet hyperboloid corresponding to the coadjoint orbit. For the dual pairing, we can observed that $J(z)=J_{1}\left(z,z^{*}\right)u_{1}^{*}+J_{2}\left(z,z^{*}\right)u_{2}^{*}+J_{3}\left(z,z^{*}\right)u_{3}^{*}\in g^{*}$ with $J_{1}(z,z^{*})=\rho \frac{1+{\left|z\right|}^{2}}{1-{\left|z\right|}^{2}},\;J_{2}(z,z^{*})=\frac{\rho }{i}\frac{z-z^{*}}{1-{\left|z\right|}^{2}},\;J_{3}(z,z^{*})=-\rho \frac{z+z^{*}}{1-{\left|z\right|}^{2}}$ and $\beta =\beta_{1}u_{1}+\beta_{2}u_{2}+\beta_{3}u3\in g^{*}$ with $\beta =2\left(r,-\eta_{R},-\eta_{I}\right)\;,\;\eta =\eta_{R}+i\eta_{I}$.

The integral of normalization in Gibbs density could be computed through Kirillov character formula by $\chi_{m}\left(\exp\left(\left(\begin{array}{cc} x & .\\ . & -x \end{array}\right)\right)\right)=j{\left(x\right)}^{-1}\int_{O_{m-1}^{+}}e^{-i\langle \left(\begin{array}{cc} x & .\\ . & -x \end{array}\right),\left(\begin{array}{cc} ir & \eta \\ \eta^{*} & -ir \end{array}\right)\rangle }\omega_{O_{m-1}^{+}}$ where

$$j(x)=\det^{1/2}\left[\sinh\left(ad\left(\begin{array}{cc} x/2 & \\ & -x/2 \end{array}\right)\right)/ad\left(\begin{array}{cc} x/2 & \\ & -x/2 \end{array}\right)\right]=\frac{\sinh(x)}{x}$$

with following relation $\frac{e^{mx}}{1-e^{2x}}j(x)=\int_{D}e^{(m-1)x\frac{1+{\left|w\right|}^{2}}{1-{\left|w\right|}^{2}}}\frac{1}{{\left(1-{\left|w\right|}^{2}\right)}^{2}}dw\wedge dw^{*}$. 

Recently, Enrico De Micheli [69] has introduced a Laplace-type transform (the so-called Spherical Laplace Transform) with a connection to the Non-Euclidean Fourier Transform in the sense of Helgason, and the principal series of the unitary representation of *SU(1,1)*.

### 6.3. Extension to SU (p,q) Unitary Group for Siegel Unit Disk

Mode details are given in Appendix B, on parameterization of SU(1,1) and extension to SU (p,q). To address computation of covariant Gibbs density for Siegel Unit Disk, we will consider in this section SU(p,q) Unitary Group:

(109) $$G=SU(p,q)\;\mathrm{and}\;K=S\left(U(p)\times U(q)\right)=\left\{\left(\begin{array}{cc} A & 0\\ 0 & D \end{array}\right)/A\in U(p),D\in U(q),\det(A)\det(D)=1\right\}$$

We can use the following decomposition for $g\in G^{C}$:

(110) $$g=\left(\begin{array}{cc} A & B\\ C & D \end{array}\right)\in G^{C},g=\left(\begin{array}{cc} I_{p} & BD^{-1}\\ 0 & I_{q} \end{array}\right)\left(\begin{array}{cc} A-BD^{-1}C & 0\\ 0 & D \end{array}\right)\left(\begin{array}{cc} I_{p} & 0\\ D^{-1}C & I_{q} \end{array}\right)$$

and consider the action of $g\in G^{C}$ on Siegel Unit Disk $SD=\left\{Z\in M_{pq}\left(C\right)/I_{p}-ZZ^{+}>0\right\}$ given by:

(111) $$g=\left(\begin{array}{cc} A & B\\ C & D \end{array}\right)\in G^{C},g=\left(\begin{array}{cc} I_{p} & BD^{-1}\\ 0 & I_{q} \end{array}\right)\left(\begin{array}{cc} A-BD^{-1}C & 0\\ 0 & D \end{array}\right)\left(\begin{array}{cc} I_{p} & 0\\ D^{-1}C & I_{q} \end{array}\right)$$

Benjamin Cahen has study this case and introduced the moment map by identifing G-equivariantly $g^{*}$ with g by means of the Killing form β on $g^{C}$:

$$g^{*}\;G-\mathrm{equivariant}\;\mathrm{with}\;g\mathrm{by}\;\mathrm{Killing}\;\mathrm{form}\;\beta \left(X,Y\right)=2(p+q)Tr\left(XY\right)\;$$

The set of all elements of g fixed by K is 𝖍:

(112) $$\begin{array}{l} 𝖍=\left\{\mathrm{element}\;\mathrm{of}\;G\;\mathrm{fixed}\;\mathrm{by}\;K\right\}\;,\;\xi_{0}\in 𝖍,\xi_{0}=i\lambda \left(\begin{array}{cc} -qI_{p} & 0\\ 0 & pI_{q} \end{array}\right)\\ \Rightarrow \langle \xi_{0},\left[Z,Z^{+}\right]\rangle =-2i\lambda {\left(p+q\right)}^{2}Tr\left(ZZ^{+}\right),\forall Z\in D \end{array}$$

Then, we the equivatiant moment map is given by:

(113) $$\begin{array}{l} \forall X\in g^{C}\;,\;Z\in D,\psi \left(Z\right)=Ad^{*}\left(\exp\left(-Z^{+}\right)\zeta \left(\exp Z^{+}\exp Z\right)\right)\xi_{0}\\ \forall g\in G,Z\in D\;\mathrm{then}\;\psi \left(g.Z\right)=Ad_{g}^{*}\psi (Z)\\ \psi \;\mathrm{is}\;a\;\mathrm{diffeomorphism}\;\mathrm{from}\;SD\;\mathrm{onto}\;\mathrm{orbit}\;O\left(\xi_{0}\right) \end{array}$$

with:

(114) $$\psi \left(Z\right)=i\lambda \left(\begin{array}{cc} {\left(I_{p}-ZZ^{+}\right)}^{-1}\left(-pZZ^{+}-qI_{p}\right) & \left(p+q\right)Z{\left(I_{q}-Z^{+}Z\right)}^{-1}\\ -(p+q){\left(I_{q}-Z^{+}Z\right)}^{-1}Z^{+} & \left(pI_{q}+qZ^{+}Z\right){\left(I_{q}-Z^{+}Z\right)}^{-1} \end{array}\right)$$

(115) $$\zeta \left(\exp Z^{+}\exp Z\right)=\left(\begin{array}{cc} I_{p} & Z{\left(I_{q}-Z^{+}Z\right)}^{-1}\\ 0 & I_{q} \end{array}\right)$$

## 7. Lie Groups Thermodynamics for SE(2) Lie Group

After SU(1,1) Lie group with null cohomology and then without Souriau one-cocycle, we will consider Souriau model for SE(2) Lie group with non-null cohomology and then with introduction of Souriau one-cocycle [70].

We will consider first SO(2) Lie group:

(116) $$SO(2)=\left\{R_{\varphi }=\left[\begin{array}{cc} \cos\varphi & -\sin\varphi \\ \sin\varphi & \cos\varphi \end{array}\right]/\varphi \in R\right\}$$

A vector at the identity to SO(2) is given by:

(117) $${\frac{dR_{t\eta }}{dt}|}_{t=0}=-\eta ℑ\;\mathrm{with}\;ℑ=\left[\begin{array}{cc} 0 & 1\\ -1 & 0 \end{array}\right],{ℑ}^{T}={ℑ}^{-1}=-ℑ$$

We consider the special Euclidean group $SE(2)=SO\left(2\right)\times R^{2}$.

(118) $$SE(2)=\left\{\left[\begin{array}{cc} R_{\varphi } & \tau \\ 0 & 1 \end{array}\right]/R_{\varphi }\in SO\left(2\right),\tau \in R^{2}\right\}$$

the group operation is given by:

(119) $$\begin{array}{l} \left[\begin{array}{cc} R_{\varphi_{1}} & \tau_{1}\\ 0 & 1 \end{array}\right]\left[\begin{array}{cc} R_{\varphi_{2}} & \tau_{2}\\ 0 & 1 \end{array}\right]=\left[\begin{array}{cc} R_{\varphi_{1}}R_{\varphi_{2}} & R_{\varphi_{1}}\tau_{2}+\tau_{1}\\ 0 & 1 \end{array}\right]=\left[\begin{array}{cc} R_{\varphi_{1}+\varphi_{2}} & R_{\varphi_{1}}\tau_{2}+\tau_{1}\\ 0 & 1 \end{array}\right]\\ \Rightarrow \left(R_{{}_{1}},\tau_{1}\right).\left(R_{\varphi_{2}},\tau_{2}\right)=\left(R_{\varphi_{1}+\varphi_{2}},R_{\varphi_{1}}\tau_{2}+\tau_{1}\right) \end{array}$$

(120) $${\left[\begin{array}{cc} R_{\varphi_{1}} & \tau_{1}\\ 0 & 1 \end{array}\right]}^{-1}=\left[\begin{array}{cc} R_{-\varphi_{1}} & -R_{-\varphi_{1}}\tau_{1}\\ 0 & 1 \end{array}\right]\Rightarrow {\left(R_{\varphi_{1}},\tau_{1}\right)}^{-1}=\left(R_{-\varphi_{1}},-R_{-\varphi_{1}}\tau_{1}\right)$$

The Lie algebra se(2) of SE(2) has underlying vector space $R^{3}$ and Lie bracket:

(121) $$\left(\xi ,u\right)\in se(2)=R\times R^{2}\Rightarrow \left[\begin{array}{cc} -\xi ℑ & u\\ 0 & 0 \end{array}\right]\in se\left(2\right)$$

Lie bracket is given by:

(122) [(ξ,u),(η,v)]=(0,ξℑv+ηℑu)

Adjoint action of SE(2) is given by:

(123) $$\begin{array}{l} Ad_{\left(R_{\varphi },\tau \right)}\left(\xi ,u\right)=\left[\begin{array}{cc} R_{\varphi } & \tau \\ 0 & 1 \end{array}\right]\left[\begin{array}{cc} -\xi ℑ & u\\ 0 & 0 \end{array}\right]\left[\begin{array}{cc} R_{-\varphi } & -R_{-\varphi }\tau \\ 0 & 1 \end{array}\right]=\left[\begin{array}{cc} -\xi ℑ & \xi ℑ\tau +R_{\varphi }u\\ 0 & 0 \end{array}\right]\\ Ad_{\left(R_{\varphi },\tau \right)}\left(\xi ,u\right)=\left(\xi ,R_{\varphi }u+\xi ℑ\tau \right) \end{array}$$

Coadjoint action of SE(2) is given by:

(124) $$Ad_{\left(R_{\varphi },\tau \right)}^{*}\left(m,\rho \right)=\left(m+ℑR_{\varphi }\rho .\tau ,R_{\varphi }\rho \right)$$

The moment map $J:R^{2}\to se^{*}\left(2\right)$ of SE(2) is defined by:

(125) $$J_{\left(\xi ,u\right)}(x)=J(x).\left(\xi ,u\right)$$

with the right action of SE(2) on $R^{2}$:

(126) $$x.\left(R\varphi ,\tau \right)=R_{-\varphi }\left(x-\tau \right)$$

the infinitesimal generator of (ξ,u)∈se(2) has the expression:

(127) $${\left(\xi ,u\right)}_{R^{2}}(x)={\frac{d\left[x.\left(R_{t\xi },tu\right)\right]}{dt}|}_{t=0}={\frac{d\left[R_{-t\xi }\left(x-tu\right)\right]}{dt}|}_{t=0}=\xi ℑx-u$$

Let $J_{\left(\xi ,u\right)}(x):R^{2}\to se^{*}\left(2\right)$ be the moment map of this action relative to the symplectic form, we can compute it from its definition:

(128) $$\begin{array}{l} dJ_{\left(\xi ,u\right)}(x).y=-2\omega \left({\left(\xi ,u\right)}_{R^{2}},y\right)\\ \mathrm{with}\;\omega \left({\left(\xi ,u\right)}_{R^{2}},y\right)=\omega \left(\xi ℑx-u,y\right)=\left(\xi ℑx-u\right).ℑy=\left(\xi x+ℑu\right).y\\ \Rightarrow dJ_{\left(\xi ,u\right)}(x).y=-2\left(\xi x+ℑu\right).y\\ \Rightarrow J_{\left(\xi ,u\right)}(x)=-2\left(\frac{1}{2}\xi {\left\|x\right\|}^{2}+ℑu.x\right)=-2\left(\frac{1}{2}{\left\|x\right\|}^{2},-ℑx\right).\left(\xi ,u\right)\\ J_{\left(\xi ,u\right)}(x)=J(x).\left(\xi ,u\right)\Rightarrow J(x)=-2\left(\frac{1}{2}{\left\|x\right\|}^{2},-ℑx\right)\;,\;x\in R^{2} \end{array}$$

We then compute the one-cocycle of SE(2) from the moment map:

(129) $$\begin{array}{l} \theta \left(\left(R_{\varphi ,\tau }\right)\right)=J\left(0.\left(R_{\varphi },\tau \right)\right)-Ad_{\left(R_{\varphi },\tau \right)}^{*}J(0)=J\left(-R_{-\varphi }\tau \right)\\ \theta \left(\left(R_{\varphi ,\tau }\right)\right)=-2\left(\frac{1}{2}{\left\|\tau \right\|}^{2},ℑR_{-\varphi }\tau \right)=-2\left(\frac{1}{2}{\left\|\tau \right\|}^{2},R_{-\varphi -\frac{\pi }{2}}\tau \right) \end{array}$$

Coadjoint orbit of SE(2) are generated by:

(130) $$\begin{array}{l} O_{\left(m,\rho \right)}=\left\{A_{\left(R_{\varphi ,\tau }\right)}^{*}\left(m,\rho \right)+\theta \left(\left(R_{\varphi },\tau \right)\right)/\left(R_{\varphi },\tau \right)\in SE\left(2\right)\right\}\\ O_{\left(m,\rho \right)}=\left\{\left(x-R_{-\frac{\pi }{2}}\rho .\tau -{\left\|\tau \right\|}^{2},R_{-\varphi }\rho -2R_{-\varphi -\frac{\pi }{2}}\tau \right)/\left(R_{\varphi },\tau \right)\in SE\left(2\right)\right\} \end{array}$$

The Souriau Symplectic form in this case of non-null cohomology is given by:

(131) $$\begin{array}{l} \omega_{\left(m,\rho \right)\left(m^{\prime },\rho^{\prime }\right)}\left(ad_{\left(\xi ,u\right)}^{*}\left(m^{\prime },\rho^{\prime }\right)-\left(0,2ℑu\right),ad_{\left(\eta ,v\right)}^{*}\left(m^{\prime },\rho^{\prime }\right)-\left(0,2ℑv\right)\right)=\rho^{\prime }.\left(-\xi ℑv+\eta ℑu\right)+2u.ℑv\\ \mathrm{with}\;\left(m^{\prime },\rho^{\prime }\right)=\left(x-R_{-\frac{\pi }{2}}\rho .\tau -{\left\|\tau \right\|}^{2},R_{-\varphi }\rho -2R_{-\varphi -\frac{\pi }{2}}\tau \right)\in O_{\left(m,\rho \right)}\subset R^{3} \end{array}$$

With the expression of moment map, we can compute Souriau covariant Gibbs density of Maximum Entropy.

Considering the symplectic form $\omega \left(\zeta ,\upsilon \right)=\zeta .ℑ\upsilon \;\mathrm{with}\;ℑ=\left[\begin{array}{cc} 0 & 1\\ -1 & 0 \end{array}\right]$ on $R^{2}$, we have seen that the action of *SE*(2) is symplectic and admits the momentum map, $J(x)=-\left(\frac{1}{2}{\left\|x\right\|}^{2},-ℑx\right)\;,\;x\in R^{2}$.

Souriau Gibbs density is defined for generalized temperature $\beta \in \Omega =\left\{\left(b,Β\right)\in se(2)/b<0,Β\in R^{2}\right\}$ and given by:

(132) $$p_{Gibbs}\left(x\right)=\frac{e^{-\langle J\left(x\right),\beta \rangle }}{\int_{R^{2}}e^{-\langle J\left(x\right),\beta \rangle }d\lambda (x)}=\frac{e^{\frac{1}{2}b{\left\|x\right\|}^{2}-Β.ℑx}}{\int_{R^{2}}e^{\frac{1}{2}b{\left\|x\right\|}^{2}-Β.ℑx}d\lambda (x)}$$

The Massieu Potential could be computed:

(133) $$\Phi \left(\beta \right)=\log\int_{R^{2}}e^{\frac{1}{2}b{\left\|x\right\|}^{2}-Β.ℑx}d\lambda (x)=\log\left(-\frac{2\pi }{b}e^{-\frac{1}{2b}{\left\|B\right\|}^{2}}\right)$$

By derivation of Massieu potential, we can deduce expression of Heat:

(134) $$\begin{array}{l} Q\in \Omega^{*}=\left\{\left(m,M\right)\in se^{*}(2)/m+\frac{{\left\|M\right\|}^{2}}{2}<0\right\}\\ Q=\frac{\partial \Phi \left(\beta \right)}{\partial \beta }=\left(\frac{1}{b}-\frac{{\left\|Β\right\|}^{2}}{2b^{2}},\frac{1}{b}Β\right)=\Theta \left(\beta \right) \end{array}$$

We can the inverse this relation to express generalized temperature with respect to the heat:

(135) $$\beta =\Theta^{-1}\left(Q\right)=\left({\left(m+\frac{1}{2}{\left\|M\right\|}^{2}\right)}^{-1},{\left(m+\frac{1}{2}{\left\|M\right\|}^{2}\right)}^{-1}M\right)$$

We can the express the Gibbs density with respect to the Heat Q which is the mean of moment map:

(136) $$p_{Gibbs}\left(x\right)=\frac{e^{\frac{\frac{1}{2}{\left\|x\right\|}^{2}-M.ℑx}{\left(m+\frac{1}{2}{\left\|M\right\|}^{2}\right)}}}{\int_{R^{2}}e^{\frac{\frac{1}{2}{\left\|x\right\|}^{2}-M.ℑx}{\left(m+\frac{1}{2}{\left\|M\right\|}^{2}\right)}}d\lambda (x)}\;\mathrm{with}\;\left(m,M\right)=E\left(J(x)\right)=E\left[-2\left(\frac{1}{2}{\left\|x\right\|}^{2},-ℑx\right)\right]=\left[-E\left({\left\|x\right\|}^{2}\right),2ℑE(x)\right]$$

So we can rewrite the Gibbs density:

(137) $$p_{Gibbs}\left(x\right)=\frac{e^{\frac{\frac{1}{2}{\left\|x\right\|}^{2}+2E(x).Ix}{\left(-E\left({\left\|x\right\|}^{2}\right)+2{\left\|E(x)\right\|}^{2}\right)}}}{\int_{R^{2}}e^{\frac{\frac{1}{2}{\left\|x\right\|}^{2}+2E(x).Ix}{\left(-E\left({\left\|x\right\|}^{2}\right)+2{\left\|E(x)\right\|}^{2}\right)}}d\lambda (x)}$$

We can also provide a Fisher metric in dual Lie algebra as hessian of the Entropy:

(138) $$S\left(Q\right)=\langle Q,\beta \rangle -\Phi \left(\beta \right)=1+\log\left(2\pi \right)+\log\left(-m-\frac{{\left\|M\right\|}^{2}}{2}\right)$$

(139) $$I_{Fisher}\left(Q\right)={\left(m+\frac{1}{2}{\left\|M\right\|}^{2}\right)}^{-1}\left[\begin{array}{cc} I & M^{T}\\ M^{T} & \frac{1}{2}{\left\|M\right\|}^{2}-m \end{array}\right]$$

and as $\left(m,M\right)=E\left(J(x)\right)=E\left[-2\left(\frac{1}{2}{\left\|x\right\|}^{2},-ℑx\right)\right]=\left[-E\left({\left\|x\right\|}^{2}\right),2ℑE(x)\right]$, Fisher metric in dual space of Lie Algebra parameterization could be written:

(140) $$I_{Fisher}\left(Q\right)={\left(2{\left\|E(x)\right\|}^{2}-E\left({\left\|x\right\|}^{2}\right)\right)}^{-1}\left[\begin{array}{cc} I & {\left(2ℑE(x)\right)}^{T}\\ 2ℑE(x) & 2{\left\|E(x)\right\|}^{2}+E\left({\left\|x\right\|}^{2}\right) \end{array}\right]$$

## 8. New Entropy Definition as Generalized Casimir Invariant Functions for Coadjoint and Adjoint Representation

In his paper written in 1974, Jean-Marie Souriau has observed that if we consider the heat expression $Q=\frac{d\Phi }{d\beta }$, that we can write δΦ−〈Q,δβ〉=0. For each δβ tangent to the orbit, and so generated by an element Z of the Lie algebra, if we consider the relation $\Phi \left(Ad_{g}(\beta )\right)=\Phi \left(\beta \right)-\langle \theta \left(g^{-1}\right),\beta \rangle$ and we differentiate it at g=e using the property that $\tilde{\Theta }(X,Y)=-\langle d\theta (X),Y\rangle \;,\;X,Y\in g$, we obtain $\langle Q,\left[\beta ,Z\right]\rangle +\tilde{\Theta }\left(\beta ,Z\right)=0$. Souriau has stopped by this last equation, the characterization of Group action on $Q=\frac{\partial \Phi }{\partial \beta }$. Souriau has also observed that $S\left[Q\left(Ad_{g}(\beta )\right)\right]=S\left[Ad_{g}^{*}(Q)+\theta \left(g\right)\right]=S\left(Q\right)$. We propose to characterize more explicitly this invariance, by characterizing Entropy as an invariant Casimir function in coadjoint representation.

From last Souriau equation, if we use the identities $\beta =\frac{\partial S}{\partial Q}$, $ad_{\beta }Z=\left[\beta ,Z\right]$ and $\tilde{\Theta }\left(\beta ,Z\right)=\langle \Theta \left(\beta \right),Z\rangle$, then we can deduce that $\langle ad_{\frac{\partial S}{\partial Q}}^{*}Q+\Theta \left(\frac{\partial S}{\partial Q}\right),Z\rangle =0,\forall Z$. So, Entropy S(Q) should verify $ad_{\frac{\partial S}{\partial Q}}^{*}Q+\Theta \left(\frac{\partial S}{\partial Q}\right)=0$, characterizes an invariant Casimir function in case of non-null cohomology, that we propose to write with Poisson brackets, ${\left\{S,H\right\}}_{\tilde{\Theta }}\left(Q\right)=0$ where ${\left\{S,H\right\}}_{\tilde{\Theta }}\left(Q\right)=\langle Q,\left[\frac{\partial S}{\partial Q},\frac{\partial H}{\partial Q}\right]\rangle +\tilde{\Theta }\left(\frac{\partial S}{\partial Q},\frac{\partial H}{\partial Q}\right)=0$, $\forall H:g^{*}\to R,Q\in g^{*}$.

In a Poisson manifold, Casimir functions $S\in C^{\infty }(g^{*})$, in case of null cohomology, are functions whose Poisson brackets will all functions vanish, $\left\{S,H\right\}\left(Q\right)=0\;,\forall S\in C^{\infty }(g^{*}),Q\in g^{*}$. In the dual of the Lie algebra of a connected Lie group G, the Casimir functions are the $Ad^{*}$-invariant functions, because if $S,H\in C^{\infty }(g^{*})$ and $Q\in g^{*}$, then $\left\{S,H\right\}\left(Q\right)=\langle Q,\left[\frac{\partial S}{\partial Q},\frac{\partial H}{\partial Q}\right]\rangle =\langle Q,ad_{\frac{\partial S}{\partial Q}}\frac{\partial H}{\partial Q}\rangle =\langle ad_{\frac{\partial S}{\partial Q}}^{*}Q,\frac{\partial H}{\partial Q}\rangle$ vanishes for all $H\in C^{\infty }(g^{*})$ if and only if $ad_{\frac{\partial S}{\partial Q}}^{*}Q=0$. A function is S on $g^{*}$ is $Ad^{*}$-invariant if g.S=S, ∀g∈G where Lie group G acts on functions on $g^{*}$ by $\left(g.S\right)\left(Q\right)=S\left(Ad_{g}^{*}Q\right),\;Q\in g^{*},S\in C^{\infty }\left(g^{*}\right),g\in G$, and where infinitesimal characterizations of $Ad^{*}$-invariant functions on $g^{*}$, ${\frac{d}{dt}S\left(Ad_{\exp(tx)}^{*}Q\right)|}_{t=0}=\langle ad_{x}^{*}Q,\frac{\partial S}{\partial Q}\rangle =-\langle ad_{\frac{\partial S}{\partial Q}}^{*}Q,x\rangle$. The symplectic leaves of a Poisson manifold are contained in the connected components of the level sets of the Casimir functions and Casimir function is constant on a symplectic leaf. Coadjoint orbits lie on level sets of the Casimir functions, which are conserved quantities. Casimir functions Level sets are symplectic manifolds. Coadjoint motion of the moment map $Q(t)=Ad_{g(t)}^{*}Q(0)$ for a solution curve g(t)∈C(G) take place on the intersections of levels sets of the Hamiltonian and the Casimir functions. Alexis Arnaudon has studied stochastic coadjoint processes whose solutions lie on coadjoint orbits.

We have observed that ${\left\{S,H\right\}}_{\tilde{\Theta }}\left(Q\right)=\langle Q,\left[\frac{\partial S}{\partial Q},\frac{\partial H}{\partial Q}\right]\rangle +\tilde{\Theta }\left(\frac{\partial S}{\partial Q},\frac{\partial H}{\partial Q}\right)=0,\forall H:g^{*}\to R,Q\in g^{*}$, that shows that Souriau Entropy is a Casimir function in case with non-null cohomology when an additional cocycle should be taken into account. Indeed, infinitesimal variation is characterized by the following differentiation: ${\frac{d}{dt}S\left(Q\left(Ad_{\exp(tx)}\beta \right)\right)|}_{t=0}={\frac{d}{dt}S\left(Ad_{\exp(tx)}^{*}Q+\theta \left(\exp(tx)\right)\right)|}_{t=0}=-\langle ad_{\frac{\partial S}{\partial Q}}^{*}Q+\Theta \left(\frac{\partial S}{\partial Q}\right),x\rangle$. We recover extended Casimir equation in case of non-null cohomology verified by Entropy, $ad_{\frac{\partial S}{\partial Q}}^{*}Q+\Theta \left(\frac{\partial S}{\partial Q}\right)=0$, and then the generalized Casimir condition ${\left\{S,H\right\}}_{\tilde{\Theta }}\left(Q\right)=0$. Hamiltonian motion on these affine coadjoint orbits is given by the solutions of the Lie-Poisson equations with cocycle.

The identification of Entropy as an Invariant Casimir Function in Coadjoint representation is also important in Information Theory, because classically Entropy is introduced axiomatically. With this new approach, we can build Entropy by constructing the Casimir Function associated to the Lie group and also in case of non-null cohomology. Igor V. Shirokov [71,72,73,74,75] has proposed a method for constructing invariants of the coadjoint representation of Lie groups with an arbitrary dimension and structure based on local symplectic coordinates on the coadjoint orbits. The idea of the method of constructing coadjoint invariants is to construct the canonical transition to the Darboux coordinates on the orbits of the dual Lie algebra $g^{*}$ of maximal dimension dual to the Lie algebra g of the Lie group G. These relations provide invariants of the coadjoint representation of the Lie group G.

This geometric framework unifies several earlier works on the subject, including Souriau’s symplectic model of statistical mechanics, and approaches developed in Information Geometry and Quantum Information Geometry. This approach helps to identify the common geometric structures appearing in various domains from statistical mechanics to statistical learning. The emphasis is put on the role of the affine equivariance with respect to Lie group actions, as extension of the Fisher metric in presence of equivariance and the associated Lie-Poisson equations with cocycle (affine Lie-Poisson equations). The entropy of the Souriau model as a Casimir function can be used to apply a geometric model for energy preserving entropy production on Lie algebras. We can exploit the geometric framework of this new equation to build geometric numerical integrator schemes for some of the equations associated to Souriau’s model and its polysymplectic extension. This new equation is important because it introduce new structure of differential equations in case of non-null cohomology and for an arbitrary Hamiltonian $H:g^{*}\to R$: $\frac{dQ}{dt}=ad_{\frac{\partial H}{\partial Q}}^{*}Q+\Theta \left(\frac{\partial H}{\partial Q}\right)$.

The equation $\frac{dQ}{dt}=ad_{\frac{\partial H}{\partial Q}}^{*}Q+\Theta \left(\frac{\partial H}{\partial Q}\right)$ is important because it allows extending stochastic perturbation of the Lie-Poisson equation with cocycle within the setting of stochastic Hamiltonian dynamics, which preserves the affine coadjoint orbits. We can extend model for stochastic geometric modeling in fluid dynamics via variational principles described in [32,76]. This extension results in the new Stratonovich differential equation for the stochastic process $dQ+\left[ad_{\frac{\partial H}{\partial Q}}^{*}Q+\Theta \left(\frac{\partial H}{\partial Q}\right)\right]dt+\sum_{i=1}^{N}\left[ad_{\frac{\partial H_{i}}{\partial Q}}^{*}Q+\Theta \left(\frac{\partial H_{i}}{\partial Q}\right)\right]\circ dW_{i}\left(t\right)=0$.

This new equation is also very usefull for geometric symplectic Lie group integrator for Lie-Poisson equations with cocycle that preserves the affine coadjoint orbits for general Hamiltonian. This equation is also very relevant in the framework of dynamics with Casimir dissipation/production, to formulate a dynamical geometric model for dissipation/production of this Casimir. This allows to extend the general Lie algebraic approach developed in [77,78] for Casimir dissipation, to take into account of a cocycle, and to a wider class of dissipation. Paper [17] will exploit this new Casimir equation in case of non-null cohomology.

This equation $\frac{dQ}{dt}=ad_{\frac{\partial H}{\partial Q}}^{*}Q+\Theta \left(\frac{\partial H}{\partial Q}\right)$ could be used also to make the link with 2nd principle of Thermodynamique, that will be deduced from positivity of Souriau-Fisher metric:

(141) $$\begin{array}{l} S\left(Q\right)=\langle Q,\beta \rangle -\Phi \left(\beta \right)\;\mathrm{with}\;\frac{dQ}{dt}=ad_{\frac{\partial H}{\partial Q}}^{*}Q+\Theta \left(\frac{\partial H}{\partial Q}\right)\\ \frac{dS}{dt}=\langle Q,\frac{d\beta }{dt}\rangle +\langle ad_{\frac{\partial H}{\partial Q}}^{*}Q+\Theta \left(\frac{\partial H}{\partial Q}\right),\beta \rangle -\frac{d\Phi }{dt}=\langle Q,\frac{d\beta }{dt}\rangle +\langle ad_{\frac{\partial H}{\partial Q}}^{*}Q,\beta \rangle ++\langle \Theta \left(\frac{\partial H}{\partial Q}\right),\beta \rangle -\frac{d\Phi }{dt}\\ \frac{dS}{dt}=\langle Q,\frac{d\beta }{dt}\rangle +\langle Q,\left[\frac{\partial H}{\partial Q},\beta \right]\rangle +\tilde{\Theta }\left(\frac{\partial H}{\partial Q},\beta \right)-\frac{d\Phi }{dt}=\langle Q,\frac{d\beta }{dt}\rangle +{\tilde{\Theta }}_{\beta }\left(\frac{\partial H}{\partial Q},\beta \right)-\langle \frac{\partial \Phi }{\partial \beta },\frac{d\beta }{dt}\rangle \\ \frac{dS}{dt}=\langle Q,\frac{d\beta }{dt}\rangle +{\tilde{\Theta }}_{\beta }\left(\frac{\partial H}{\partial Q},\beta \right)-\langle \frac{\partial \Phi }{\partial \beta },\frac{d\beta }{dt}\rangle \;\mathrm{with}\;\frac{\partial \Phi }{\partial \beta }=Q\\ \frac{dS}{dt}={\tilde{\Theta }}_{\beta }\left(\frac{\partial H}{\partial Q},\beta \right)\geq 0,\forall H\;(\mathrm{link}\;\mathrm{to}\;\mathrm{positivity}\;\mathrm{of}\;\mathrm{Fisher}\;\mathrm{metric})\\ \mathrm{if}\;H=S\underset{\frac{\partial S}{\partial Q}=Q}{\Rightarrow }\frac{dS}{dt}={\tilde{\Theta }}_{\beta }\left(\beta ,\beta \right)=0\;\mathrm{because}\;\beta \in Ker{\tilde{\Theta }}_{\beta } \end{array}$$

Entropy production is then linked with Souriau-Fisher structure, $dS={\tilde{\Theta }}_{\beta }\left(\frac{\partial H}{\partial Q},\beta \right)dt$ with ${\tilde{\Theta }}_{\beta }\left(\frac{\partial H}{\partial Q},\beta \right)=\tilde{\Theta }\left(\frac{\partial H}{\partial Q},\beta \right)+\langle Q,\left[\frac{\partial H}{\partial Q},\beta \right]\;\rangle$ Souriau tensor related to Fisher metric.

### 8.1. Casimir Invariant and Generalized Casimir Invariant

Hendrik Brugt Gerhard Casimir, a Dutch physicist, studied what is called Casimir operators and Casimir invariants (H. Casimir and Van der Waerden studied the SU(2) group, the group of isospin/angular momentum, as the model of the algebraic approach to the study of the unitary representations of semi-simple compact Lie groups). Kirillov has explained that Casimir operators are in one-to-one correspondence with polynomial invariants characterizing orbits of the coadjoint representation. Solutions are not necessarily polynomials and the nonpolynomial solutions are called ***generalized Casimir invariants***. For certain classes of Lie algebras, all invariants of the coadjoint representation are functions of polynomial ones. In physics, Hamiltonians and integrals of motion of classical integrable Hamiltonian systems are not polynomials in the momenta [71,72,73,74,75,79,80,81,82,83,84,85,86,87,88,89,90,91,92].

### 8.2. Souriau Entropy as Generalized Casimir Invariant in Coadjoint Representation

In Souriau Lie groups Thermodynamics, we will see that coadjoint orbits lie on level sets of the Entropy that could be considered as a Casimir invariant function:

(142) $$\begin{array}{l} S:g^{*}\to R\\ \;Q\mapsto S\left(Q\right) \end{array}$$

We will consider first the case of null-cohomology, Entropy as Casimir invariant function is a conserved quantity, because Casimir function has null Lie Poisson brackets functions [93,94]:

(143) $$\begin{array}{l} \left\{S,H\right\}\left(Q\right)=\langle Q,\left[\frac{\partial S}{\partial Q},\frac{\partial H}{\partial Q}\right]\rangle =0,\forall H:g^{*}\to R,Q\in g^{*}\;,\;\langle A,B\rangle =B\left(A,B\right)\;\mathrm{Cartan-Killing}\;\mathrm{form}\\ \mathrm{with}\;\partial S\left(Q\right)={\frac{d}{d\varepsilon }S\left(Q+\delta Q\right)|}_{\varepsilon =0}=\langle \delta Q,\frac{\partial S}{\partial Q}\rangle \end{array}$$

We can observe that $\beta =\frac{\partial S}{\partial Q}$, then:

(144) $$\langle Q,\left[\beta ,\frac{\partial H}{\partial Q}\right]\rangle =\langle Q,ad_{\beta }\frac{\partial H}{\partial Q}\rangle =0,\forall H:g^{*}\to R,Q\in g^{*}\;,\;ad_{a}b=\left[a,b\right]$$

We can also write:

(145) $$\langle Q,\left[\frac{\partial S}{\partial Q},\frac{\partial H}{\partial Q}\right]\rangle =\langle Q,ad_{\frac{\partial S}{\partial Q}}\frac{\partial H}{\partial Q}\rangle =\langle ad_{\frac{\partial S}{\partial Q}}^{*}Q,\frac{\partial H}{\partial Q}\rangle =0\;,\;\forall H:g^{*}\to R$$

It means that $ad_{\frac{\partial S}{\partial Q}}^{*}Q=ad_{\beta }^{*}Q=0\;,\;\beta =\frac{\partial S}{\partial Q}$. We can remark that if we note ${\left(ad_{\frac{\partial S}{\partial Q}}^{*}Q\right)}_{j}=C_{ij}^{k}ad_{{\left(\frac{\partial S}{\partial Q}\right)}^{i}}^{*}Q_{k}=0$ with $C_{ij}^{k}$ the structure tensor, we observe that this equation is in fact the Casimir condition for invariant function in coadjoint representation as we will see hereafter. The restriction of the Lie-Poisson bracket to an orbit generates a symplectic structure on the orbit, called the KKS (Kirillov-Kostant-Souriau) structure, or the canonical symplectic structure. Casimir function is characterized as a quantity which commutes with each linear functional on the Poisson manifold, and then it is conserved by dynamics of any Hamiltonian.

Given a Hamiltonian $H:g^{*}\to R$, the equation of motion for $Q\in g^{*}$ is:

(146) $$\frac{dQ}{dt}=\left\{Q,H\right\}=ad_{\frac{\partial H}{\partial Q}}^{*}Q\;\mathrm{with}\;H=S\Rightarrow \frac{dQ}{dt}=\left\{Q,S\right\}=ad_{\frac{\partial S}{\partial Q}}^{*}Q=0$$

In case of non-null cohomology, the Lie Poisson brackets functions are given by:

(147) $$\begin{array}{l} {\left\{S,H\right\}}_{\tilde{\Theta }}\left(Q\right)=\langle Q,\left[\frac{\partial S}{\partial Q},\frac{\partial H}{\partial Q}\right]\rangle +\tilde{\Theta }\left(\frac{\partial S}{\partial Q},\frac{\partial H}{\partial Q}\right)=0,\forall H:g^{*}\to R,Q\in g^{*}\\ \mathrm{with}\;\tilde{\Theta }(X,Y)=J_{\left[X,Y\right]}-\left\{J_{X},J_{Y}\right\}\;\mathrm{where}\;J_{X}(x)=\langle J(x),X\rangle \\ \tilde{\Theta }\left(X,Y\right):g\times g\to ℜ\;\mathrm{with}\;\Theta (X)=T_{e}\theta \left(X(e)\right)\\ \;X,Y\mapsto \langle \Theta (X),Y\rangle \end{array}$$

That we can develop in the following:

(148) $$\begin{array}{l} {\left\{S,H\right\}}_{\tilde{\Theta }}\left(Q\right)=\langle Q,\left[\frac{\partial S}{\partial Q},\frac{\partial H}{\partial Q}\right]\rangle +\langle \Theta \left(\frac{\partial S}{\partial Q}\right),\frac{\partial H}{\partial Q}\rangle =0\\ {\left\{S,H\right\}}_{\tilde{\Theta }}\left(Q\right)=\langle Q,ad_{\frac{\partial S}{\partial Q}}\frac{\partial H}{\partial Q}\rangle +\langle \Theta \left(\frac{\partial S}{\partial Q}\right),\frac{\partial H}{\partial Q}\rangle =0\\ {\left\{S,H\right\}}_{\tilde{\Theta }}\left(Q\right)=\langle ad_{\frac{\partial S}{\partial Q}}^{*}Q,\frac{\partial H}{\partial Q}\rangle +\langle \Theta \left(\frac{\partial S}{\partial Q}\right),\frac{\partial H}{\partial Q}\rangle =0\\ \forall H,{\left\{S,H\right\}}_{\tilde{\Theta }}\left(Q\right)=\langle ad_{\frac{\partial S}{\partial Q}}^{*}Q+\Theta \left(\frac{\partial S}{\partial Q}\right)+,\frac{\partial H}{\partial Q}\rangle =0\Rightarrow ad_{\frac{\partial S}{\partial Q}}^{*}Q+\Theta \left(\frac{\partial S}{\partial Q}\right)=0 \end{array}$$

We have found the generalized Casimir equation for Entropy in the non-null cohomology case:

(149) $${\left\{S,H\right\}}_{\tilde{\Theta }}\left(Q\right)=0$$

That could be also written:

(150) $$ad_{\frac{\partial S}{\partial Q}}^{*}Q+\Theta \left(\frac{\partial S}{\partial Q}\right)=0$$

This equation was observed by Souriau in his paper of 1974, where he has written that geometric temperature β is a kernel of ${\tilde{\Theta }}_{\beta }$, that is written:

(151) $$\beta \in Ker{\tilde{\Theta }}_{\beta }\Rightarrow \langle Q,\left[\beta ,Z\right]\rangle +\tilde{\Theta }\left(\beta ,Z\right)=0$$

That we can develop to recover the Casimir equation:

(152) $$\begin{array}{l} \Rightarrow \langle Q,ad_{\beta }Z\rangle +\tilde{\Theta }\left(\beta ,Z\right)=0\Rightarrow \langle ad_{\beta }^{*}Q,Z\rangle +\tilde{\Theta }\left(\beta ,Z\right)=0\\ \beta =\frac{\partial S}{\partial Q}\Rightarrow \langle ad_{\frac{\partial S}{\partial Q}}^{*}Q,Z\rangle +\tilde{\Theta }\left(\frac{\partial S}{\partial Q},Z\right)=\langle ad_{\frac{\partial S}{\partial Q}}^{*}Q+\Theta \left(\frac{\partial S}{\partial Q}\right),Z\rangle =0,\forall Z\\ \Rightarrow ad_{\frac{\partial S}{\partial Q}}^{*}Q+\Theta \left(\frac{\partial S}{\partial Q}\right)=0 \end{array}$$

Then the generalized Casimir Equation in non-null cohomogy is given by:

(153) $${\left(ad_{\frac{\partial S}{\partial Q}}^{*}Q\right)}_{j}+\Theta {\left(\frac{\partial S}{\partial Q}\right)}_{j}=C_{ij}^{k}ad_{{\left(\frac{\partial S}{\partial Q}\right)}^{i}}^{*}Q_{k}+\Theta_{j}=0$$

Given a Hamiltonian $H:g^{*}\to R$, the equation of motion for $Q\in g^{*}$ is:

(154) $$\frac{dQ}{dt}=ad_{\frac{\partial H}{\partial Q}}^{*}Q+\Theta \left(\frac{\partial H}{\partial Q}\right)\;\mathrm{with}\;H=S\Rightarrow \frac{dQ}{dt}=ad_{\frac{\partial S}{\partial Q}}^{*}Q+\Theta \left(\frac{\partial S}{\partial Q}\right)=0$$

Level sets of the Casimir Entropy function, on which the coadjoint orbits lie, are symplectic manifolds.

### 8.3. Souriau Entropy Invariance in Coadjoint Representation

If we note $𝕬𝖓\left(g^{*}\right)$ the space of analytic function on the dual space of the Lie agebra $g^{*}$, a function $F^{*}\in 𝕬𝖓\left(g^{*}\right)$ is a Casimir invariant if for any $g\in G,X\in g^{*}$, we have $F^{*}\left(Ad_{g}^{*}X\right)=F^{*}(X)$. We have observed previously that Souriau’s Entropy analytic function S(Q) defined on dual space of the Lie algebra $g^{*}$ by Legendre transform of Massieu Characteric analytic function Φ(β) (minus logarithm of Laplace transform) defined on Lie algebra g was an invariant function under the affine coadjoint action $S\left[Q\left(Ad_{g}(\beta )\right)\right]=S\left[Ad_{g}^{*}(Q)+\theta \left(g\right)\right]=S\left(Q\right)$. In case of null-cohomology, Souriau cocycle cancels θ(g)=0, and we recover Casimir invariant function in coadjoint representation $S\left[Ad_{g}^{*}(Q)\right]=S\left(Q\right)$.

***We can then observe that Souriau Entropy is an extended Casimir invariant function in case of non-null cohomogy. This characteristic of Souriau Entropy could be a new characterization of Entropy. In Souriau Lie groups Thermodynamics, Entropy S(Q) is a generalized Casimir invariant function for coadjoint representation in case of non-null cohomology, and Massieu Characteristic function by Legendre duality is a generalized Casimir function for adjoint representation.***

We will explain how to prove that Souriau Entropy is invariant under the action of the group, starting from its definition:

(155) $$S(Q)=\langle Q,\beta \rangle -\Phi (\beta )\;\mathrm{with}\;Q=\frac{\partial \Phi (\beta )}{\partial \beta }\in g^{*}\;\mathrm{and}\;\beta =\frac{\partial S(Q)}{\partial Q}\in g$$

with

(156) $$\Phi (\beta )=-\log\int_{M}e^{-\langle U(\xi ),\beta \rangle }d\lambda_{\omega }\;\mathrm{and}\;U:M\to g^{*}$$

Considering Souriau Entropy S(Q) where the heat $Q=\frac{\partial \Phi (\beta )}{\partial \beta }\in g^{*}$ an element of the dual space of the Lie algebra is parameterized by β∈g an element of the Lie algebra, the Lie group G acts through g∈G by adjoint operator $Ad_{g}$, the entropy is given by $S\left[Q\left(Ad_{g}(\beta )\right)\right]$ with $Q\left(Ad_{g}(\beta )\right)$ given by fundamental Souriau equation:

(157) $$Q\left(Ad_{g}(\beta )\right)=Ad_{g}^{*}(Q)+\theta \left(g\right)$$

The invariance of Souriau Entropy is deduced from the following developments:

(158) $$\begin{array}{l} \beta \in g\to Ad_{g}(\beta )\Rightarrow \Psi (Ad_{g}(\beta ))=\int_{M}e^{-\langle U,Ad_{g}(\beta )\rangle }d\lambda_{\omega }\\ \Psi (Ad_{g}(\beta ))=\int_{M}e^{-\langle Ad_{g^{-1}}^{*}U,\beta \rangle }d\lambda_{\omega }=\int_{M}e^{-\langle U\left(Ad_{g^{-1}}\beta \right)-\theta \left(g^{-1}\right),\beta \rangle }d\lambda_{\omega }\\ \Psi (Ad_{g}(\beta ))=e^{\langle \theta \left(g^{-1}\right),\beta \rangle }\Psi \left(\beta \right)\\ \theta \left(g^{-1}\right)=-Ad_{g^{-1}}^{*}\theta (g)\Rightarrow \Psi (Ad_{g}(\beta ))=e^{-\langle Ad_{g^{-1}}^{*}\theta (g),\beta \rangle }\Psi \left(\beta \right)\\ \Phi \left(\beta \right)=-\log\Psi (\beta )\Rightarrow \Phi \left(Ad_{g}(\beta )\right)=\Phi \left(\beta \right)-\langle \theta \left(g^{-1}\right),\beta \rangle =\Phi \left(\beta \right)+\langle Ad_{g^{-1}}^{*}\theta (g),\beta \rangle \end{array}$$

Based on this expression of Massieu Characteristic function transform by action of the group, we can use Legendre transform to study how Souriau Entropy is changed:

(159) $$\begin{array}{l} S(Q)=\langle Q,\beta \rangle -\Phi (\beta )\Rightarrow S\left(Q\left(Ad_{g}\beta \right)\right)=\langle Q\left(Ad_{g}\beta \right),Ad_{g}\beta \rangle -\Phi \left(Ad_{g}\beta \right)\\ \left\{ \begin{array}{l} Q\left(Ad_{g}(\beta )\right)=Ad_{g}^{*}(Q)+\theta \left(g\right)\\ \Phi \left(Ad_{g}(\beta )\right)=-\log\Psi (Ad_{g}(\beta ))=-\langle \theta \left(g^{-1}\right),\beta \rangle +\Phi \left(\beta \right) \end{array}\right.\\ \Rightarrow S\left(Q\left(Ad_{g}\beta \right)\right)=\langle Ad_{g}^{*}(Q)+\theta \left(g\right),Ad_{g}\beta \rangle +\langle \theta \left(g^{-1}\right),\beta \rangle -\Phi \left(\beta \right)\\ \Rightarrow S\left(Q\left(Ad_{g}\beta \right)\right)=\langle Ad_{g}^{*}(Q)+\theta \left(g\right),Ad_{g}\beta \rangle -\langle Ad_{g^{-1}}^{*}\theta (g),\beta \rangle -\Phi \left(\beta \right)\\ \Rightarrow S\left(Q\left(Ad_{g}\beta \right)\right)=\langle Ad_{g^{-1}}^{*}Ad_{g}^{*}(Q)+Ad_{g^{-1}}^{*}\theta \left(g\right),\beta \rangle -\langle Ad_{g^{-1}}^{*}\theta (g),\beta \rangle -\Phi \left(\beta \right)\\ Ad_{g^{-1}}^{*}Ad_{g}^{*}(Q)=Q\Rightarrow S\left(Q\left(Ad_{g}\beta \right)\right)=\langle Q,\beta \rangle -\Phi \left(\beta \right)=S\left(\beta \right) \end{array}$$

We finally prove that Souriau Entropy is invariant in coadjoint representation $S\left(Ad_{g}^{*}(Q)+\theta \left(g\right)\right)=S\left(\beta \right)$ in general case of non-null cohomology, that we could write $S\left(Ad_{g}^{\#}(Q)\right)=S\left(\beta \right)$, if we note affine coadjoint action $Ad_{g}^{\#}(Q)=Ad_{g}^{*}(Q)+\theta \left(g\right)$. This is also true in case of null-cohomology when the Souriau cocycle cancels θ(g)=0, and we recover classical generalized Casimir invariant function definition on coadjoint representation for Entropy $S\left(Ad_{g}^{*}(Q)\right)=S\left(\beta \right)$ generalized Casimir invariant function definition on adjoint representation for Massieu Characteristic function $\Phi \left(Ad_{g}(\beta )\right)=\Phi \left(\beta \right)$.

### 8.4. Souriau Entropy Given by Casimir Invariant Functions Equations

Based on development given in the following we can state that:

As the Entropy S is a generalized Casimir invariant function in the coadjoint representation, $S\left(Ad_{e^{t\xi }}^{*}h\right)=S(h)$, then S should be solution of the following differential equation:

(160) $$C_{ij}^{k}Q_{k}\frac{\partial S\left(Q\right)}{\partial Q_{j}}=0\;,\;i,j,k=\dim g,\;\mathrm{with}\;\left\{ \begin{array}{l} C_{ij}^{k}Q_{k}=C_{ij}(Q)=B_{ij}\\ B_{Q}\left(x,y\right)=B_{ij}x^{i}y^{j}=\langle Q,\left[x,y\right]\rangle \end{array}\right.$$

where $C_{ij}^{k}$ is the structure tensor of the Lie algebra g in the basis $\left(e_{1},e_{2},\ldots ,e_{n}\right)$, while $X_{k}$ are the coordinates in $g^{*}$ in the basis $\left(e^{1},e^{2},\ldots ,e^{n}\right)$ defined by $\langle e^{j},e_{i}\rangle =\delta_{ij}$. The structure tensor s given by $\left[\phi \left(e_{i}\right),\phi \left(e_{i}\right)\right]=C_{ij}^{k}\phi \left(e_{k}\right)$ with $\phi \left(e_{i}\right)=C_{ij}^{k}X_{k}\frac{\partial }{\partial X_{j}}\;,\;i=1,\ldots ,n$.

### 8.5. Characterization of Generalized Casimir Invariant Functions in Coadjoint Representation

We will describe recent characterization of generalized Casimir invariant functions by Oleg L. Kurnyavko and Igor V. Shirokov [72,73,75] who have proposed Algebraic method for construction of Casimir invariants of Lie groups coadjoint representations (see Appendix C). Modern invariant theory based on geometric methods, which was credited classically as non-constructive, has some exception admitting a constructive solution related to the constructing invariants of Lie groups representations.

Let T be a connected Lie group, T(G) a representation of the group G in the linear space V, $T_{g}$ the operators associated to the representation of the group G on the linear space V, then the invariants are given by the following equation:

(161) $$F\left(T_{g}x\right)=F(x)\;,\;x\in V,g\in G,T_{g}\in T(G),F(x)\in C^{\infty }(V)$$

With the properties that:

(162) $$T_{e}=I\;,\;T_{g_{a}g_{b}}=T_{g_{a}}T_{g_{b}}\;,\;T_{g^{-1}}={\left(T_{g}\right)}^{-1}$$

Solution is given by the following differential equation:

(163) $$-\sum_{i,j}^{\dim V}t_{kj}^{i}x^{j}\frac{\partial F(x)}{\partial x^{i}}=0\;\mathrm{with}\;t_{kj}^{i}={\frac{\partial {\left(T_{g}\right)}_{j}^{i}}{\partial g^{k}}|}_{g=e}\;\mathrm{and}\;k=1,\ldots ,\dim G$$

$t_{kj}^{i}$ are elements of the matrices of the Lie algebra representation basis of G.

That we can write $t_{k}=-t_{kj}^{i}x^{j}\frac{\partial }{\partial x^{i}}$ and $t_{k}F(x)=0$.

If we consider the dual space $V^{*}$, the co-tangent representation is given by:

(164) $$\langle T^{*}(g)X,T(g)x\rangle =\langle X,x\rangle$$

And co-represnetation invariants are given by:

(165) $$t_{k}^{*}F^{*}(X)=0\;\mathrm{with}\;t_{k}^{*}=t_{kj}^{i}X_{i}\frac{\partial }{\partial X_{j}}$$

They have underlined the relationship between invariants of representations and conjugate representations, where the algebraic construction of Lie groups representations invariants are given by invariants of the conjugate representation with respect to the invariants of the original representation.

**Shirokov Theorem 1.**  *Let* F(x) *be a non-degenerate invariant of the representation* T(G) *, then conjugate representation invariant can be found by Legrendre tranform:*

(166) $$F^{*}(X)=x^{i}X_{i}-F(x)=\langle x,X\rangle -F(x)\;\mathrm{with}\;X=\frac{\partial F(x)}{\partial x}\;\mathrm{such}\;\mathrm{that}\;X_{i}=\frac{\partial F(x)}{\partial x^{i}}$$

*and also the converse problem:*

(167) $$F(x)=x^{i}X_{i}-F^{*}(X)=\langle x,X\rangle -F^{*}(X)\;\mathrm{with}\;x=\frac{\partial F^{*}(X)}{\partial X}\;\mathrm{such}\;\mathrm{that}\;x^{i}=\frac{\partial F^{*}(X)}{\partial X_{i}}$$

Shirokov has considered F(x) the representation invariant T(G), and $F^{*}(X)$ the representation invariant $T^{*}(G)$ conjugate to T(G), with the conditions:

(168) $$-t_{kj}^{i}x^{j}\frac{\partial F(x)}{\partial x^{i}}=0\;\mathrm{and}\;t_{lj}^{i}X_{i}\frac{\partial F^{*}(X)}{\partial X_{j}}=0$$

(169) $$\begin{array}{l} t_{lj}^{i}X_{i}\frac{\partial F^{*}(X)}{\partial X_{j}}=t_{lj}^{i}X_{i}\frac{\partial }{\partial X_{j}}\left[x^{k}(X)X_{k}-F\left(x(X)\right)\right]=t_{lj}^{i}X_{i}\frac{\partial x^{k}}{\partial X_{j}}X_{k}+t_{lj}^{i}X_{i}x^{k}\frac{\partial X_{k}}{\partial X_{j}}-t_{lj}^{i}X_{i}\frac{\partial F(x)}{\partial x^{k}}\frac{\partial x^{k}}{\partial X_{j}}\\ t_{lj}^{i}X_{i}\frac{\partial F^{*}(X)}{\partial X_{j}}=t_{lj}^{i}X_{i}\frac{\partial x^{k}}{\partial X_{j}}\frac{\partial F(x)}{\partial x^{k}}+t_{lj}^{i}\frac{\partial F(x)}{\partial x^{i}}x^{k}\delta_{k}^{j}-t_{lj}^{i}\frac{\partial F(x)}{\partial x^{i}}\frac{\partial F(x)}{\partial x^{k}}\frac{\partial x^{k}}{\partial X_{j}}\\ t_{lj}^{i}X_{i}\frac{\partial F^{*}(X)}{\partial X_{j}}=t_{lj}^{i}x_{j}\frac{\partial F(x)}{\partial x^{i}}=0 \end{array}$$

Invariant Casimir Functions of the coadjoint representation has been studied for completely integrable Hamiltonian systems, as classical systems on the orbits of the coadjoint representation. Oleg L. Kurnyavko and Igor V. Shirokov have considered the relationship between invariants of representations of Lie groups and their conjugate dual representations.

Considering the coadjoint action given by:

(170) $$\langle Ad_{g}^{*}X,x\rangle =\langle X,Ad_{g^{-1}}x\rangle \;,\;g\in G,X\in g^{*},x\in g$$

Invariants of a coadjoint representation are called Casimir functions, with the property:

(171) $$F^{*}\left(Ad_{g}^{*}X\right)=F^{*}\left(X\right)$$

the infinitesimal invariance is given by the equations:

(172) $$C_{ij}(X)\frac{\partial F^{*}\left(X\right)}{\partial X_{j}}=0\;\mathrm{with}\;C_{ij}(X)=C_{ij}^{k}X_{k},\;i,j,k=\dim g$$

The number of functionally independent invariants is given by the rank of the matrix $C_{ij}(X)$, called the index of the Lie algebra g: $indg=\dim g^{*}-\underset{X\in g^{*}}{\sup}rankC_{ij}(X)$. 

From these adjoint and coadjoint representation, Shirokov has introduced the following theorem:

**Shirokov Theorem 2.**  *Let*$F\left(Ad_{g}x\right)=F(x)$*be a non-degenerate invariant of the adjoint representation*$Ad_{G}$*, then conjugate representation invariant, invariant of coadjoint representation*$Ad_{G}^{*}$can be found by formula:

(173) $$F^{*}(X)=x^{i}X_{i}-F(x)=\langle x,X\rangle -F(x)\;\mathrm{with}\;X=\frac{\partial F(x)}{\partial x}\;\mathrm{such}\;\mathrm{that}\;X_{i}=\frac{\partial F(x)}{\partial x^{i}}$$

*and also the converse problem, let*$F^{*}\left(Ad_{g}^{*}X\right)=F^{*}\left(X\right)$*, invariant of coadjoint representation*$Ad_{G}$ is given by:

(174) $$F(x)=x^{i}X_{i}-F^{*}(X)=\langle x,X\rangle -F^{*}(X)\;\mathrm{with}\;x=\frac{\partial F^{*}(X)}{\partial X}\;\mathrm{such}\;\mathrm{that}\;x^{i}=\frac{\partial F^{*}(X)}{\partial X_{i}}$$

Nota:

(175) $$C_{ij}^{k}X_{k}\frac{\partial F^{*}\left(X\right)}{\partial X_{j}}=0\;,\;i,j,k=\dim g,\;\mathrm{with}\;\left\{ \begin{array}{l} C_{ij}^{k}X_{k}=C_{ij}(X)=B_{ij}\\ B_{X}\left(x,y\right)=B_{ij}x^{i}y^{j}=\langle X,\left[x,y\right]\rangle \end{array}\right.$$

### 8.6. Constructing Generalized Casimir Invariant Functions in Coadjoint Representation

I. V. Shirokov has proposed a method for constructing invariants of the coadjoint representation of Lie groups with an arbitrary dimension and structure based on local symplectic coordinates on the coadjoint orbits. Oleg L. Kurnyavko and Igor V. Shirokov have also proposed a general method for constructing Casimir invariants.

We will give some other developments of Casimir Invariant Functions by A.T. Fomenko and V.V. Trofimov, related to Orbits of the coadjoint representation and the associated canonical symplectic structure.

The coadjoint orbit $O_{h}$ passing through the point $h\in g^{*}$ is given by

(176) $$O_{h}=\left\{Ad_{g}^{*}h/g\in G\right\}\;\mathrm{where}\;h\in g^{*}$$

(177) $$\begin{array}{c} T_{h}O_{h}=\left\{ad_{\rho }^{*}h/\rho \in g\right\}\subset g^{*},h\in g^{*}\\ v={\frac{d\left[Ad_{\exp^{t\rho }}^{*}h\right]}{dt}|}_{t=0}\in T_{h}O_{h}\;,\;\rho \in g \end{array}$$

(178) $$\begin{array}{l} \mathrm{Let}\;\left(e_{1},\ldots ,e_{n}\right)\;\mathrm{basis}\;\mathrm{of}\;g,\;\left(e^{1},\ldots ,e^{n}\right)\;\mathrm{basis}\;\mathrm{of}\;g^{*},\;\mathrm{with}\;\langle e^{i},e_{j}\rangle =\delta_{i,j}\\ h=h_{i}e^{i}\Rightarrow v^{i}={\frac{d\left[\langle h_{i},Ad_{\exp^{t\rho }}^{*}h\rangle \right]}{dt}|}_{t=0}={\frac{d\left[\langle Ad_{\exp^{t\rho }}^{*}h,e_{i}\rangle \right]}{dt}|}_{t=0}\\ v^{i}={\frac{d\left[\langle h,Ad_{\exp^{-t\rho }}e_{i}\rangle \right]}{dt}|}_{t=0}=\langle h,{\frac{d\left[Ad_{\exp^{-t\rho }}e_{i}\right]}{dt}|}_{t=0}\rangle \\ v^{i}=\langle h,-\left[\rho ,e_{i}\right]\rangle =-\langle ad_{\rho }^{*}h,e_{i}\rangle =\langle v,e_{i}\rangle \Rightarrow v=-ad_{\rho }^{*}h \end{array}$$

Kirillov, Kostant and Souriau have introduced a KKS 2-form on co-adjoint co-orbits that then inherit a structure of homogeneous symplectic manifold:

(179) $$\begin{array}{l} \xi ,\eta \in T_{h}O_{h}=\left\{ad_{\chi }^{*}h/\rho \in g\right\}\subset g^{*},h\in g^{*}\\ \omega_{h}\left(\xi ,\eta \right)=\omega \left(ad_{\xi_{1}}^{*}h,ad_{\eta_{1}}^{*}h\right)=\langle h,\left[\xi_{1},\eta_{1}\right]\rangle \;\mathrm{with}\;\xi =ad_{\xi_{1}}^{*}h\;\mathrm{and}\;\eta =ad_{\eta_{1}}^{*}h \end{array}$$

This KKS 2-form ω is invariant with respect to the coadjoint action $\omega_{g}\left(Ad_{f}^{*}\xi ,Ad_{f}^{*}\eta \right)=\omega_{h}\left(\xi ,\eta \right)$:

(180) $$\begin{array}{l} \omega_{g}\left(Ad_{f}^{*}\xi ,Ad_{f}^{*}\eta \right)=\omega_{g}\left(Ad_{f}^{*}ad_{\xi_{1}}^{*}h,Ad_{f}^{*}ad_{\eta_{1}}^{*}h\right)\\ \mathrm{with}\;g=Ad_{f}^{*}h\;,\;\xi =ad_{\xi_{1}}^{*}h\;,\;\eta =ad_{\eta_{1}}^{*}h\;\mathrm{and}\;f\in G,g,h\in g^{*}\\ Ad_{f}^{*}ad_{\xi_{1}}^{*}h=ad_{Ad_{f}\xi_{1}}^{*}\left(Ad_{f}^{*}h\right)\;\mathrm{and}\;Ad_{f}^{*}ad_{\eta_{1}}^{*}h=ad_{Ad_{f}\eta_{1}}^{*}\left(Ad_{f}^{*}h\right)\\ \omega_{g}\left(Ad_{f}^{*}\xi ,Ad_{f}^{*}\eta \right)=\omega_{g}\left(ad_{Ad_{f}\xi_{1}}^{*}\left(Ad_{f}^{*}h\right),ad_{Ad_{f}\eta_{1}}^{*}\left(Ad_{f}^{*}h\right)\right)\\ \omega_{g}\left(Ad_{f}^{*}\xi ,Ad_{f}^{*}\eta \right)=\omega_{g}\left(ad_{Ad_{f}\xi_{1}}^{*}g,ad_{Ad_{f}\eta_{1}}^{*}g\right)=\langle g,\left[Ad_{f}\xi_{1},Ad_{f}\eta_{1}\right]\rangle \\ \omega_{g}\left(Ad_{f}^{*}\xi ,Ad_{f}^{*}\eta \right)=\langle g,Ad_{f}\left[\xi_{1},\eta_{1}\right]\rangle =\langle Ad_{f^{-1}}^{*}g,\left[\xi_{1},\eta_{1}\right]\rangle \;\mathrm{with}\;h=Ad_{f^{-1}}^{*}g\\ \omega_{g}\left(Ad_{f}^{*}\xi ,Ad_{f}^{*}\eta \right)=\langle h,\left[\xi_{1},\eta_{1}\right]\rangle \\ \omega_{g}\left(Ad_{f}^{*}\xi ,Ad_{f}^{*}\eta \right)=\omega_{h}\left(\xi ,\eta \right) \end{array}$$

The symplectic structure is given due to the property that dω=0, that could be proved making link with Jacobi identity.

(181) $$\begin{array}{l} \mathrm{Let}\;grad_{skew}m\;\mathrm{such}\;\mathrm{that}\;\omega \left(v,grad_{skew}m\right)=v(m)=\sum_{i}v^{i}\frac{\partial m}{\partial x^{i}}\;\mathrm{smooth}\;\mathrm{vector}\;\mathrm{field}\;\mathrm{on}\;M\\ \left\{m,n\right\}=\omega \left(grad_{skew}m,grad_{skew}n\right)=\sum_{i<j}\omega_{ij}{\left(grad_{skew}m\right)}^{i}{\left(grad_{skew}n\right)}^{j}\\ \mathrm{with}\;{\left(grad_{skew}m\right)}^{i}=\sum_{j}\omega^{ij}\frac{\partial m}{\partial x^{j}}\Rightarrow \left\{m,n\right\}=\sum_{i<j}\omega^{ij}\frac{\partial m}{\partial x^{i}}\frac{\partial n}{\partial x^{j}} \end{array}$$

Jacobi identity can be computed:

(182) $$\begin{array}{l} \left\{m,\left\{n,p\right\}\right\}=-\left(grad_{Skew}m\right)\left\{n,p\right\}=-L_{grad_{Skew}m}\left\{n,p\right\}\;\mathrm{with}\;L_{\zeta }:\;\mathrm{Lie}\;\mathrm{derivative}\\ \mathrm{If}\;\xi =grad_{Skew}m\\ L_{\xi }\left\{n,p\right\}=L_{\xi }\left(\omega^{ij}\frac{\partial n}{\partial x^{i}}\frac{\partial p}{\partial x^{j}}\right)=L_{\xi }{\left(\omega \right)}^{ij}\frac{\partial n}{\partial x^{i}}\frac{\partial p}{\partial x^{j}}+\omega^{ij}\frac{\partial \left(\xi n\right)}{\partial x^{i}}\frac{\partial p}{\partial x^{j}}+\omega^{ij}\frac{\partial n}{\partial x^{i}}\frac{\partial \left(\xi p\right)}{\partial x^{j}}\\ L_{\xi }\left\{n,p\right\}=L_{\xi }{\left(\omega \right)}^{ij}\frac{\partial n}{\partial x^{i}}\frac{\partial p}{\partial x^{j}}+\left\{\xi n,p\right\}+\left\{n,\xi p\right\}=L_{\xi }{\left(\omega \right)}^{ij}\frac{\partial n}{\partial x^{i}}\frac{\partial p}{\partial x^{j}}-\left\{\left\{m,n\right\},p\right\}-\left\{n,\left\{m,p\right\}\right\}\\ \Rightarrow \left\{m,\left\{n,p\right\}\right\}+\left\{\left\{m,n\right\},p\right\}+\left\{n,\left\{m,p\right\}\right\}=L_{\xi }{\left(\omega \right)}^{ij}\frac{\partial n}{\partial x^{i}}\frac{\partial p}{\partial x^{j}} \end{array}$$

Using Elie Cartan formula $L_{\xi }\omega =i\left(\xi \right)d\omega +di\left(\xi \right)\omega$. If ξ is a Hamiltonian vector field, di(ξ)ω=0 and then $L_{\xi }\omega =i\left(\xi \right)d\omega$. If dω=0, then the Jacobi identity is satisfied {m,{n,p}}+{{m,n},p}+{n,{m,p}}=0 and conversely.

Let consider the Berezin Bracket:

(183) $$\begin{array}{l} \left\{m,n\right\}=-C_{ij}^{k}x_{k}\frac{\partial m}{\partial x^{i}}\frac{\partial n}{\partial x^{j}}\;\mathrm{with}\;\left[e_{i},e_{j}\right]=C_{ij}^{k}e_{k}\\ \mathrm{where}\;\left(e_{1},e_{2},\ldots ,e_{n}\right)\;\mathrm{basis}\;\mathrm{of}\;\mathrm{Lie}\;\mathrm{algebra}\;g,\left(e^{1},e^{2},\ldots ,e^{n}\right)\mathrm{basis}\;\mathrm{of}\;\mathrm{dual}\;\mathrm{Lie}\;\mathrm{algebra}\;g^{*}\\ \mathrm{of}\;\mathrm{corresponding}\;\mathrm{coordinates}\;x^{1},\ldots ,x^{n}\;\mathrm{for}\;g,\;x_{1},\ldots ,x_{n}\;\mathrm{for}\;g^{*} \end{array}$$

This Berezin Bracket is given by:

(184) $$\begin{array}{l} {\left\{m,n\right\}}_{x}=dm_{x}\left(ad_{dn(x)}^{*}(x)\right)=\left(ad_{dn(x)}^{*}(x)\right)\left(dm_{x}\right)\\ {\left\{m,n\right\}}_{x}=\langle x,\left[dn_{x},dm_{x}\right]\rangle =C_{ij}^{k}x_{k}\frac{\partial n}{\partial x^{i}}\frac{\partial m}{\partial x^{j}}\;\mathrm{with}\;dn_{x}=\frac{\partial n}{\partial x_{i}}e_{i}\;,\;dm_{x}=\frac{\partial m}{\partial x_{j}}e_{j} \end{array}$$

By developping Berezin Bracket $\left\{m,n\right\}=-C_{ij}^{k}x_{k}\frac{\partial m}{\partial x^{i}}\frac{\partial n}{\partial x^{j}}\;\mathrm{with}\;\left[e_{i},e_{j}\right]=C_{ij}^{k}e_{k}$, we can prove that the bracket verify jacoby identy {m,{n,p}}+{{m,n},p}+{n,{m,p}}=0 and then dω=0. 

We will see that differential equation for (semi-)invariants of the coadjoint representations could be established. We will note $𝕬𝖓\left(g^{*}\right)$ the space of analytic function on the dual space of the Lie agebra $g^{*}$. A function $F^{*}\in 𝕬𝖓\left(g^{*}\right)$ is an invariant if for any $g\in G,X\in g^{*}$, we have $F^{*}\left(Ad_{g}^{*}X\right)=F^{*}(X)$, and is semi-invariant if $F^{*}\left(Ad_{g}^{*}X\right)=\chi (g)F^{*}(X)$ where χ(g) is a character of the Lie group G.

We have a representation of Lie algebras ϕ:g→Vec(Γ) defined on basis $\left(e_{1},e_{2},\ldots ,e_{n}\right)$ in g where Vec(Γ) is the space of vector fields on Γ an open subset in $g^{*}$, given by:

(185) $$\phi \left(e_{i}\right)=C_{ij}^{k}X_{k}\frac{\partial }{\partial X_{j}}\;,\;i=1,\ldots ,n$$

where $C_{ij}^{k}$ is the structure tensor of the Lie algebra g in the basis $\left(e_{1},e_{2},\ldots ,e_{n}\right)$, while $X_{k}$ are the coordinates in $g^{*}$ in the basis $\left(e^{1},e^{2},\ldots ,e^{n}\right)$ defined by $\langle e^{j},e_{i}\rangle =\delta_{ij}$. The representation is not dependent of the choice of the basis, with the property: $\left[\phi \left(e_{i}\right),\phi \left(e_{i}\right)\right]=C_{ij}^{k}\phi \left(e_{k}\right)$.

We have the property, that:

(186) $${\frac{d^{n}F^{*}\left(Ad_{e^{t\xi }}^{*}h\right)}{dt^{n}}|}_{t=0}=\left[{\left(-\phi \left(\xi \right)\right)}^{n}F^{*}\right]\left(h\right)$$

This result is obtained by the following development:

(187) $$\begin{array}{l} {\frac{dF^{*}\left(Ad_{e^{t\xi }}^{*}h\right)}{dt}|}_{t=0}=\frac{\partial F^{*}}{\partial X_{i}}(h){.\frac{d\langle Ad_{e^{t\xi }}^{*}h,X_{i}\rangle }{dt}|}_{t=0}=\frac{\partial F^{*}}{\partial X_{i}}(h){.\frac{d\langle h,Ad_{e^{-t\xi }}e_{i}\rangle }{dt}|}_{t=0}\\ {\frac{d\langle h,Ad_{e^{-t\xi }}e_{i}\rangle }{dt}|}_{t=0}=\langle h,-\left[\xi ,e_{i}\right]\rangle =-\langle h,C_{ij}^{k}\xi^{j}e_{k}\rangle \;\mathrm{with}\;\left[\xi ,e_{i}\right]=\left[\xi^{j}e_{j},e_{i}\right]=C_{ji}^{k}\xi^{j}e_{k}\\ {\frac{d\langle h,Ad_{e^{-t\xi }}e_{i}\rangle }{dt}|}_{t=0}=\langle h_{k}e^{k},-C_{ji}^{k}\xi^{j}e_{k}\rangle =-C_{ji}^{k}\xi^{j}h_{k}\\ {\frac{dF^{*}\left(Ad_{e^{t\xi }}^{*}h\right)}{dt}|}_{t=0}=-C_{ji}^{k}\xi^{j}h_{k}\frac{\partial F^{*}}{\partial X_{i}}\left(\xi \right)=\left(-\phi \left(\xi \right)F^{*}\right)(h) \end{array}$$

We use then Taylor expansion of $F^{*}\left(Ad_{e^{t\xi }}^{*}h\right)$ given by:

(188) $$F^{*}\left(Ad_{e^{t\xi }}^{*}h\right)=F^{*}(h)+\sum_{n=1}^{\infty }\frac{{\left(-\phi (\xi )\right)}^{n}F^{*}}{n!}(h).t^{n}$$

We can observe that $F^{*}$ is invariant if $F^{*}\left(Ad_{e^{t\xi }}^{*}h\right)=F^{*}(h)$ and then ${\left(-\phi (\xi )\right)}^{n}F^{*}=0$ or $\phi (\xi )F^{*}=0$ that could be written $C_{ji}^{k}\xi^{j}h_{k}\frac{\partial F^{*}}{\partial X_{i}}\left(\xi \right)=0$.

If $F^{*}$ is semi-invariant of the coadjoint representation of group if and only if:

$$\phi (e_{i})F^{*}=-\lambda_{i}F^{*}\;\mathrm{with}\;\lambda_{i}=d\chi \left(e_{i}\right)\;(d\chi \;:\;\mathrm{derivative}\;\mathrm{of}\;\chi \;\mathrm{at}\;\mathrm{the}\;\mathrm{group}\;G\;\mathrm{identity}\;\mathrm{element}$$

(189) $$\begin{array}{l} F^{*}\left(Ad_{e^{t\xi }}^{*}h\right)=\chi \left(e^{t\xi }\right)F^{*}(h)\;\mathrm{with}\;\chi \left(e^{t\xi }\right)=e^{t\chi_{*}\left(\xi \right)}\\ \left[{\left(-\phi \left(\xi \right)\right)}^{n}F^{*}\right]\left(h\right)=\chi_{*}\left(\xi \right)F^{*}(h)\\ \Rightarrow F^{*}\left(Ad_{e^{t\xi }}^{*}h\right)=\left[1+\sum_{n=1}^{\infty }\frac{{\left[\chi_{*}\left(\xi \right)\right]}^{n}}{n!}t^{n}\right].F^{*}(h) \end{array}$$

## 9. Conclusion: Lie Groups Thermodynamics for Machine Learning

With Lie groups Thermodynamics, we have presented Souriau tools to extend Gibbs density for Lie groups [95,96,97,98,99,100,101,102,103,104,105,106,107]. We can make reference to other explorations of Lie Group Representation theory to built exponential families [108,109,110,111] or Information Geometry in Quantum Physics [112,113,114,115,116,117,118,119,120,121,122,123]. Gibbs density estimation is a basic tool in statistical macine learning. Classically, we can associate to any posterior distribution an effective generalized geometric temperature, given by an element of the dual space of the Lie algebra, relating it to the Gibbs prior distribution. Classification rules could be introduced by Gibbs measures defined on parameter sets and depending on the observed sample value. A Gibbs measure is a special kind of probability measure used in statistical mechanics to describe the state of a particle system driven by a given energy function at some given temperature. Gibbs measures will be realized as minimizers of the average loss value under entropy constraints. In this extension for Lie groups, an important tool is the log-Laplace transform related to the Massieu Characteristic Function in Thermodynamics (a re-parameterization of the free energy by Planck temperature preserving Legendre transform with respect to Entropy). As we want to deal with Lie group data for Machine Learning, we will consider tools very similar to those used in statistical mechanics to describe particle systems with many degrees of freedom. Classification rules could be described by Gibbs measures defined on parameter sets and depending on the observed sample value. Comparing any posterior distribution with a Gibbs prior distribution make it possible to provide a way to build an estimator which can be proved to reach adaptively at the best possible asymptotic error rate (by temperature selection of a Gibbs posterior distribution built within a single parametric model). Estimators derived from Gibbs posteriors show excellent performance in diverse tasks, such as classification, regression and ranking. The usual recommendation is to sample from a Gibbs posterior using MCMC (Markov chain Monte Carlo). With covariant Souriau Gibbs density, it is possible to extend MCMC and Gibbs sampler approach for Lie Groups Machine Learning.

More recently, the use of perturbation techniques has been proposed as an alternative to MCMC techniques for sampling. These results have been extended in conditional random fields loss, proving that the maximum in expectation with low-rank perturbations, provides an upperbound on the log partition (what we call Massieu characteristic function). New lower bounds on the partition function and new unbiased sequential sampler for the Gibbs distribution based on low-rank perturbations have been introduced. All these methods are based on sampling from the Gibbs distribution, upper-bounding the log partition function. All these results are synthetized in [124], where they also propose a new general method, with connections to the recently-proposed Fenchel-Young losses [125], using doubly stochastic scheme for minimization of these losses, for unsupervised and supervised learning. This is a generalization to the Gibbs distribution. Methods for learning parameters of a Gibbs distribution on data ${\left(y_{i}\right)}_{i=1,\ldots ,n}$ are based on maximization of the likelihood:

(190) $${\hat{l}}_{n}\left(\theta \right)=\frac{1}{n}\sum_{i=1}^{n}\log p_{Gibbs,\theta }\left(y_{i}\right)=\frac{1}{n}\sum_{i=1}^{n}\langle y_{i},\theta \rangle -\log\psi \left(\theta \right)$$

that is optimized by gradients methods using the empirical log-likelihood, given by:

(191) $$\nabla_{\theta }{\hat{l}}_{n}\left(\theta \right)={\hat{y}}_{n}-E_{Gibbs,\theta }\left[y\right]$$

For this method of moment-matching, the expectation of the Gibbs distribution is a challenge in some cases. This approach has been replaced by computing $p_{\theta }$, with a method called “*perturb-and-MAP*” to learn the parameters in this model as a proxy for log-likelihood. This minimization is equivalent to maximizing previous equation by substituting the log-partition logψ(θ) with:

(192) $$\begin{array}{c} F_{\varepsilon }(\theta )=E\left[F\left(\theta +\varepsilon V\right)\right]=E\left[\underset{y\in C}{\max\langle y,\theta +\varepsilon V\rangle }\right]\;\mathrm{with}\;a\;\mathrm{random}\;\mathrm{noise}\;\mathrm{vector}\\ \varepsilon V,\varepsilon >0 \end{array}$$

This approach could be linked with the use of Fenchel-Young losses [125]. In the perturbed model, the Fenchel-Young loss is given by:

(193) $$L_{\varepsilon }\left(\theta ;y\right)=F_{\varepsilon }(\theta )+\varepsilon \Omega (y)-\langle \theta ,y\rangle =D_{\varepsilon \Omega }\left(y,{\hat{y}}_{\varepsilon }^{*}(\theta )\right)$$

with loss gradient $\nabla L_{\varepsilon }\left(\theta ;y\right)=\nabla F_{\varepsilon }(\theta )-y=y_{\varepsilon }^{*}(\theta )-y$ where $y_{\varepsilon }^{*}(\theta )=E_{p_{\theta }(y)}\left[y\right]=E\left[\arg\underset{y\in C}{\max}\langle y,\theta +\varepsilon V\rangle \right]$ and $D_{\varepsilon \Omega }\left(y,{\hat{y}}_{\varepsilon }^{*}(\theta )\right)$ Bregman divergence associated to εΩ. As $F_{\varepsilon }$ generalizes the log-sum-exp function on the simplex, its dual Ω is a generalization of the negative entropy (which is the Fenchel dual of log-sum-exp).These connections have been studied in [126].

To conclude, we have seen that Lie group tools based on Representation Theory and Orbits Methods could be used with Souriau-Fisher Metric on Coadjoint Orbits as an extension of Fisher Metric for Lie group through homogeneous Symplectic Manifolds on Lie group Co-Adjoint Orbits. 

**Figure 6:**
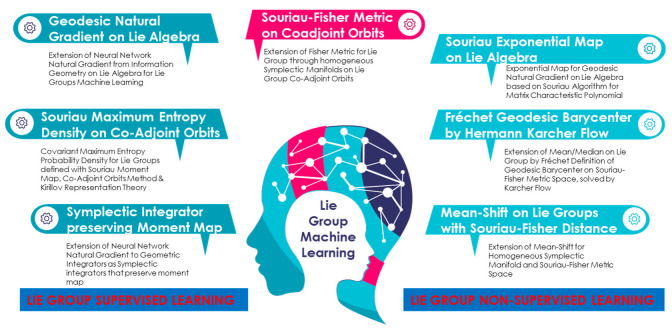
Supervised/Non-Supervised Machine Learning based on Lie Groups Thermodynamics.

We can then beneficiate of different tools based on Souriau Lie groups Thermodynamics and Kirillov Representation Theory, as illustrated in Figure 6, for:

- **Supervised Machine Learning**

⚬ Geodesic Natural Gradient on Lie Algebra: Extension of Neural Network Natural Gradient from Information Geometry on Lie Algebra for Lie Groups Machine Learning.
⚬ Souriau Maximum Entropy Density on Co-Adjoint Orbits: Covariant Maximum Entropy Probability Density for Lie groups defined with Souriau Moment Map, Co-Adjoint Orbits Method & Kirillov Representation Theory
⚬ Symplectic Integrator preserving Moment Map: Extension of Neural Network Natural Gradient to Geometric Integrators as Symplectic integrators that preserve moment map

- **Non-Supervised Machine Learning**

⚬ Souriau Exponential Map on Lie Algebra: Exponential Map for Geodesic Natural Gradient on Lie Algebra based on Souriau Algorithm for Matrix Characteristic Polynomial
⚬ Fréchet Geodesic Barycenter by Hermann Karcher Flow: Extension of Mean/Median on Lie group by Fréchet Definition of Geodesic Barycenter on Souriau-Fisher Metric Space, solved by Karcher Flow.
⚬ Mean-Shift on Lie groups with Souriau-Fisher Distance: Extension of Mean-Shift for Homogeneous Symplectic Manifold and Souriau-Fisher Metric Space.

 *[There is nothing more in physical theories than symmetry groups except the mathematical construction which allows precisely to show that there is nothing more]    « Il n’y a rien de plus dans les théories physiques que les groupes de symétrie si ce n’est la construction mathématique qui permet précisément de montrer qu’il n’y a rien de plus ».*

**Figure 7:**
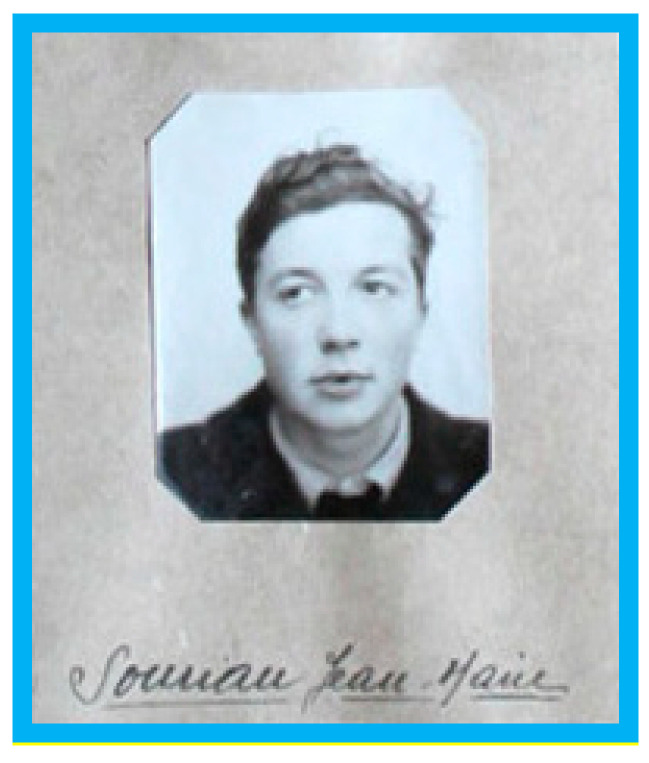
Jean-Marie Souriau, student at ENS Paris 1942.

**Jean-Marie Souriau** (see Figure 7)

  *La notion classique d’ensemble canonique de Gibbs est étendue au cas d’une variété symplectique sur laquelle un groupe de Lie possède une action symplectique (“groupe dynamique”). La définition rigoureuse donnée ici permet d’étendre un certain nombre de propriétés thermodynamiques classiques (la température est ici un élément de l’algèbre de Lie du groupe, la chaleur un élément de son dual), notamment des inégalités de convexité. Dans le cas de groupes non commutatifs, des propriétés particulières apparaissent: la symétrie est spontanément brisée, certaines relations de type cohomologique sont vérifiées dans l’algèbre de Lie du groupe [The classical notion of Gibbs’ canonical ensemble is extended to the case of a symplectic manifold on which a Lie group has a symplectic action (“dynamic group”). The rigorous definition given here makes it possible to extend a certain number of classical thermodynamic properties (the temperature here is an element of the Lie group algebra, heat an element of its dual), notably inequalities of convexity. In the case of non-commutative groups, particular properties appear: the symmetry is spontaneously broken, certain relations of cohomological type are verified in the Lie algebra of the group].*

**Jean-Marie Souriau, Mécanique Statistique, Groupes de Lie et Cosmologie, colloque CNRS n°237 – Géométrie Symplectique et physique mathématique**

## Appendix A. Coadjoint orbits and Moment Map for SU(1,1)

We give more details developpements to obtain *SU(1,1)*/K coadjoint orbit and moment map from [127]. SU(1,1) has been intensitively studied for Coherent states in Quantum Physics [128].

If we consider hyperbolic Group SL(2,R)

(A1) $$SL(2,R)=\left\{\left(\begin{array}{cc} m & p\\ q & n \end{array}\right)\in GL\left(2,R\right)/mn-pq=1\right\}$$

Elements of SL(2,R) could be written with Iwasawa decomposition:

(A2) $$g=k_{\theta }.a_{t}.n_{b}\;\mathrm{with}\;k_{\theta }\in K,a_{t}\in A,n_{b}\in N$$

(A3) $$\begin{array}{l} K=\left\{k_{\theta }=\left(\begin{array}{cc} \cos\theta & -\sin\theta \\ \sin\theta & \cos\theta \end{array}\right)/0\leq \theta <2\pi \right\}\;\mathrm{with}\;e^{-i\theta }=\frac{m-iq}{\sqrt{m^{2}+q^{2}}}\\ A=\left\{a_{t}=\left(\begin{array}{cc} e^{t} & 0\\ 0 & e^{-t} \end{array}\right)/t\in R\right\}\;\mathrm{with}\;e^{2t}=m^{2}+q^{2}\\ N=\left\{n_{b}=\left(\begin{array}{cc} 1 & b\\ 0 & 1 \end{array}\right)/b\in R\right\}\;\mathrm{with}\;b=\frac{mp+qn}{\sqrt{m^{2}+q^{2}}} \end{array}$$

K is a maximal compact sub-group of $G_{SL}$.

### Appendix A.1. Group of Unit Disk Automorphisms

Consider SU(1,1) sub-group of SL(2,R) given by

(A4) $$SU(1,1)=\left\{A=\left(\begin{array}{cc} a & b\\ b^{*} & a^{*} \end{array}\right)/\det(A)={\left|a\right|}^{2}-{\left|b\right|}^{2}=1,\;a,b\in R\right\}$$

The following interior automorphism that transforms SL(2,R) to SU(1,1), inducing an isomorphism between them:

(A5) $$\begin{array}{l} SL(2,R)\to SU(1,1)\\ \;g\mapsto CgC^{-1}\;\mathrm{with}\;C=\frac{1}{\sqrt{2}}\left(\begin{array}{cc} 1 & -i\\ 1 & i \end{array}\right) \end{array}$$

SU(1,1) acts on the Poincaré unit disk D={z∈C/|z|<1}:

(A6) $$g^{-1}.z=\frac{az+b}{b^{*}+a^{*}}\;\mathrm{with}\;g^{-1}=\left(\begin{array}{cc} a & b\\ b^{*} & a^{*} \end{array}\right)\in SU(1,1)\;\mathrm{and}\;z\in D$$

Valentine Bargmann has parameterized SU(1,1):

(A7) $$\left\{ \begin{array}{l} \gamma =\frac{b}{a}\\ \omega =\arg\left(\alpha \right)\;\mathrm{mod}\;2\pi \end{array}\right.\Rightarrow \left\{ \begin{array}{l} \left|\gamma \right|<1\\ a=e^{i\omega }{\left(1-{\left|\gamma \right|}^{2}\right)}^{-1/2}\\ b=e^{i\omega }\gamma {\left(1-{\left|\gamma \right|}^{2}\right)}^{-1/2} \end{array}\right.$$

Then SU(1,1)={(γ,ω)/|γ|<1,ω∈]−π,+π]} with Group composition law given by:

(A8) $$\begin{array}{l} \left(\gamma ,\omega \right).\left(\gamma^{\prime },\omega^{\prime }\right)=\left(\gamma^{\prime \prime },\omega^{\prime \prime }\right)\\ \left\{ \begin{array}{l} \gamma^{\prime \prime }=\left[\gamma^{\prime }+\gamma e^{-2i\omega^{\prime \prime }}\right]{\left[1+\gamma \gamma^{{*}^{\prime }}e^{2i\omega^{\prime }}\right]}^{-1}\\ \omega^{\prime \prime }=\omega +\omega^{\prime }+\frac{1}{2i}\log\left(\left[1+\gamma^{{*}^{\prime }}\gamma e^{-2i\omega^{\prime }}\right]{\left[1+\gamma^{\prime }\gamma^{*}e^{-2i\omega^{\prime }}\right]}^{-1}\right)\;\mathrm{mod}\;2\pi \end{array}\right.\\ g=\left(\gamma ,\omega \right)\Rightarrow g^{-1}=\left(-\gamma e^{2i\omega },-\omega \right) \end{array}$$

SU(1,1) is topological product of unit disk and circle.

### Appendix A.2. Universal covering of SL(2,R)

If we consider G={(γ,ω)/|γ|<1,ω∈R}, the following mapping:

(A9) $$\begin{array}{l} \Theta :G\to SU(1,1)\\ \;\Theta (\gamma ,\omega )=\left(\gamma ,\omega \;\mathrm{mod}\;2\pi \right) \end{array}\;\mathrm{with}\;Ker\Theta =\left\{\left(0,2k\pi \right)/k\in Z\right\}$$

Topological product of unit Disk D dans C and real straight line R is the universal covering of SU(1,1).

A maximal compact subgroup of SU(1,1) is $CKC^{-1}=\left\{\left(\begin{array}{cc} e^{-i\theta } & 0\\ 0 & e^{i\theta } \end{array}\right)/\theta \in R\right\}$ and the subgroup for G is $\Theta^{-1}\left(CKC^{-1}\right)=\left\{\left(0,\theta \right)/\theta \in R\right\}$.

Pukanszky and Sally have defined irreductible unitary representation of $S\tilde{L}\left(2,R\right)$, classified in principal serie, discrete serie and complemantary serie.

The Lie algebra g of G and SU(1,1) is given by:

(A10) $$l_{1}=\frac{1}{2}\left(\begin{array}{cc} 1 & 0\\ 0 & -1 \end{array}\right),l_{2}=\frac{1}{2}\left(\begin{array}{cc} 0 & 1\\ 1 & 0 \end{array}\right),l_{0}=\frac{1}{2}\left(\begin{array}{cc} 0 & -1\\ 1 & 0 \end{array}\right)\;$$

with the commutation relation: $\left[l_{0},l_{1}\right]=l_{2},\left[l_{1},l_{2}\right]=-l_{0},\left[l_{2},l_{0}\right]=l_{1}$.

The dual space of the Lie algebra $g^{*}$ of g to g thanks to Killing form:

(A11) $$B\left(\sum_{i=1,2,0}x_{i}l_{i},\sum_{i=1,2,0}y_{i}l_{i}\right)=2\left(x_{1}y_{1}+x_{2}y_{2}-x_{0}y_{0}\right)$$

### Appendix A.3. Coadjointes Orbits of SU(1,1)

Considering adjoint representation $Ad_{g}:g\to g$, and coadjointe representation as transpose linear mapping of $Ad_{g^{-1}}$, written by $Ad_{g}^{*}={\left(Ad_{g^{-1}}\right)}^{*}$, $Ad_{g}^{*}:g^{*}\to g^{*}$.

Let f=(η,0,0) in base $\left\{l_{1},l_{2},l_{0}\right\}$ with η>0 coadjoint orbit of f is:

(A12) $$\begin{array}{l} O_{\eta }=\left\{gfg^{-1}/g=\left(\begin{array}{cc} a & c\\ b & d \end{array}\right)\in SL(2,R)\right\}\\ O_{\eta }=\left\{\eta \left(1+2bc\right),\eta \left(bd-ac\right),\eta \left(bd+ac\right)/a,b,c,d\in R\;\mathrm{with}\;ad-bc=1\right\} \end{array}$$

Stabilizer of f is

(A13) $$G_{1}(f)=\left\{g\in G/g.f=f\right\}=A\cup \left\{-A\right\}\;\mathrm{with}\;A=\left\{a_{t}=\left(\begin{array}{cc} e^{t} & 0\\ 0 & e^{-t} \end{array}\right)/t\in R\right\}$$

$SL(2,R)=K^{\prime }NA^{\prime }\;\mathrm{with}\;A^{\prime }=A\cup \left\{-A\right\}$ where $N=\left\{n_{b}=\left(\begin{array}{cc} 1 & b\\ 0 & 1 \end{array}\right)/b\in R\right\}$ and $K^{\prime }=\left\{k_{\theta }=\left(\begin{array}{cc} \cos\theta & -\sin\theta \\ \sin\theta & \cos\theta \end{array}\right)/0\leq \theta <\pi \right\}$ and as $SL(2,R)/G_{1}(f)$ is a bijection with $O_{\eta }$, then $O_{\eta }$ is in bijection with $K^{\prime }N$, diffeomorph to $K/\left\{I_{d}\right\}\times N$. Then all element $x\in O_{\eta }$ can be written through this bijection $x=k_{\theta }n_{b},k_{\theta }\in K^{\prime },n_{b}\in N$. $O_{\eta }$ is set of points $l=\left(x_{1},x_{2},x_{0}\right)\in g^{*}$ such that: $x_{1}^{2}+x_{2}^{2}-x_{0}^{2}=\eta^{2}>0$ a one sheet hyperboloid in $g^{*}$.

We will study discrete sequence.

### Appendix A.4. Quantization of Kähler Manifold

Let (M,ω) a Kalherian manifold of dimension 2n (complex manifold with complex structure J) with 2-form g(X,Y)=ω(X,JY), a Riemannian structure on M.

We have seen that SU(1,1) is conjugate of $SL_{2}(R)$ in SL(2,R)

(A14) $$\begin{array}{l} \left(\begin{array}{cc} \alpha & \beta \\ \beta^{*} & \alpha^{*} \end{array}\right)\in SU(1,1),\left(\begin{array}{cc} a & b\\ c & d \end{array}\right)\in SL_{2}(R)\\ \left(\begin{array}{cc} \alpha & \beta \\ \beta^{*} & \alpha^{*} \end{array}\right)=\frac{-i}{2}\left(\begin{array}{cc} 1 & -i\\ 1 & i \end{array}\right)\left(\begin{array}{cc} a & b\\ c & d \end{array}\right)\left(\begin{array}{cc} i & i\\ -1 & 1 \end{array}\right) \end{array}$$

(A15) $$\left\{ \begin{array}{l} \alpha =\frac{1}{2}\left[\left(a+d\right)+i\left(b-c\right)\right]\\ \beta =\frac{1}{2}\left[\left(a-d\right)-i\left(b+c\right)\right] \end{array}\right.\Rightarrow \left\{ \begin{array}{l} \alpha +\alpha^{*}=a+d\\ \alpha -\alpha^{*}=i\left(b-c\right)\\ \beta +\beta^{*}=a-d\\ \beta -\beta^{*}=-i\left(b+c\right) \end{array}\right.\Rightarrow \left\{ \begin{array}{l} a=\frac{1}{2}\left[\left(\alpha +\alpha^{*}\right)+\left(\beta +\beta^{*}\right)\right]\\ b=\frac{1}{2i}\left[\left(\alpha -\alpha^{*}\right)-\left(\beta -\beta^{*}\right)\right]\\ c=\frac{-1}{2i}\left[\left(\alpha -\alpha^{*}\right)+\left(\beta -\beta^{*}\right)\right]\\ d=\frac{1}{2}\left[\left(\alpha +\alpha^{*}\right)-\left(\beta +\beta^{*}\right)\right] \end{array}\right.$$

Let $f=\left(0,0,h-\frac{1}{2}\right)$ in base $\left\{l_{1},l_{2},l_{0}\right\}$ where $l_{1}=\frac{1}{2}\left(\begin{array}{cc} 1 & 0\\ 0 & -1 \end{array}\right),l_{2}=\frac{1}{2}\left(\begin{array}{cc} 0 & 1\\ 1 & 0 \end{array}\right),l_{0}=\frac{1}{2}\left(\begin{array}{cc} 0 & -1\\ 1 & 0 \end{array}\right)\;$ with $h>\frac{1}{2}$ (respectively $f=\left(0,0,h+\frac{1}{2}\right)$ with $h<-\frac{1}{2}$), the coadjoint orbit of f is:

(A16) $$\begin{array}{l} O_{h}=\left\{gfg^{-1}/g=\left(\begin{array}{cc} a & c\\ b & d \end{array}\right)\in SL(2,R)\right\}\\ O_{h}=\left\{\left(\left|h\right|-\frac{1}{2}\right)\mathrm{sign}h\left(ac+bd\right);\frac{\left(\left|h\right|-\frac{1}{2}\right)\mathrm{sign}h}{2}\left(d^{2}+c^{2}-\left(a^{2}+b^{2}\right)\right);\frac{\left(\left|h\right|-\frac{1}{2}\right)\mathrm{sign}h}{2}\left(d^{2}+c^{2}+a^{2}+b^{2}\right)\right\} \end{array}$$

$O_{h}$ appears as points $l=\left(x_{1},x_{2},x_{0}\right)\in g^{*}$ such that:

(A17) $$x_{1}^{2}+x_{2}^{2}-x_{0}^{2}=-{\left({\left|h\right|}^{2}-\frac{1}{2}\right)}^{2}<0\;\mathrm{with}\;\left\{ \begin{array}{l} x_{0}>0\;\mathrm{if}\;h>\frac{1}{2}\\ x_{0}<0\;\mathrm{if}\;h<-\frac{1}{2} \end{array}\right.$$

$O_{h}$ is one of hyperboloid sheets of $g^{*}$, associated to representation $\pi_{h}$ of discrete serie of G.

(A18) $$\left\{ \begin{array}{l} x_{1}=h^{\prime }\left(ac+bd\right)\\ x_{2}=\frac{h^{\prime }}{2}\left(d^{2}-a^{2}+c^{2}-b^{2}\right)\\ x_{0}=\frac{h^{\prime }}{2}\left(d^{2}+a^{2}+c^{2}+b^{2}\right) \end{array}\right.\;\mathrm{with}\;h^{\prime }=\left(\left|h\right|-\frac{1}{2}\right)\mathrm{sign}h$$

Then, we obtain:

(A19) $$\left\{ \begin{array}{l} x_{1}=ih^{\prime }\left(\alpha \beta -\alpha^{*}\beta^{*}\right)\\ x_{2}=-h^{\prime }\left(\alpha \beta +\alpha^{*}\beta^{*}\right)\\ x_{0}=h^{\prime }\left(\alpha \alpha^{*}+\beta \beta^{*}\right) \end{array}\right.$$

If we set:

(A20) $$\left\{ \begin{array}{l} \alpha =\frac{e^{i\theta }}{\sqrt{1-{\left|z\right|}^{2}}}\\ \beta =\frac{ze^{-i\theta }}{\sqrt{1-{\left|z\right|}^{2}}} \end{array}\right.\Rightarrow \left\{ \begin{array}{l} x_{1}=\frac{ih^{\prime }\left(z-z^{*}\right)}{1-{\left|z\right|}^{2}}\\ x_{1}=\frac{-h^{\prime }\left(z+z^{*}\right)}{1-{\left|z\right|}^{2}}\\ x_{0}=\frac{h^{\prime }\left(1+{\left|z\right|}^{2}\right)}{1-{\left|z\right|}^{2}} \end{array}\right.$$

We use the parametrization of $O_{h}$ by unit disk D:

(A21) $$\begin{array}{l} D=\left\{z\in C/\left|z\right|<1\right\}\to O_{h}\\ \;z\mapsto \left(x_{1},x_{2},x_{0}\right) \end{array}$$

(A22) {x1=−2(|h|−12)signhIm(z)1−|z|2x1=−2(|h|−12)signhRe(z)1−|z|2x0=(|h|−12)signh1+|z|21−|z|2

This parametrisation provides a kahlerian structure for $O_{h}$, inherit from D.

We have $O_{h}=G/\tilde{K}=SL(2,R)/K$, and as SL(2,R) is isomorph to SU(1,1), $O_{h}$ is identified with $SU(1,1)/K_{0}=D$. Stabilizer of the origin is $K_{0}=\left\{\left(\begin{array}{cc} \alpha & 0\\ 0 & \alpha^{*} \end{array}\right)/\left|\alpha \right|=1\right\}$. Then $O_{h}$ is globally diffeomorph to D for all h.

We can compute Liouville measure on $O_{h}$. This measure is $\omega_{h}=\frac{\omega_{h}^{\prime }}{2\pi }$ where $\omega_{h}^{\prime }$ is the canonical 2-form on orbit. Parameterization on $O_{h}$ gives:

(A23) $$\omega_{h}=\frac{\omega_{h}^{\prime }}{2\pi }=\frac{-i\left(2\left|h\right|-1\right)}{2\pi \left(1-{\left|z\right|}^{2}\right)}\mathrm{sign}h{\left|dz\right|}^{2}$$

We can observe that:

(A24) $$\left\{ \begin{array}{l} dx_{1}=\frac{i}{\left(1-{\left|z\right|}^{2}\right)}\left[\left(z-z^{*}\right)\left(1-zz^{*}\right)dh^{\prime }+h^{\prime }\left(1-z^{*2}\right)dz-h^{\prime }\left(1-z^{2}\right)dz^{*}\right]\\ dx_{2}=\frac{-1}{\left(1-{\left|z\right|}^{2}\right)}\left[\left(z+z^{*}\right)\left(1-zz^{*}\right)dh^{\prime }+h^{\prime }\left(1+z^{*2}\right)dz-h^{\prime }\left(1+z^{2}\right)dz^{*}\right]\\ dx_{0}=\frac{1}{\left(1-{\left|z\right|}^{2}\right)}\left[\left(1+zz^{*}\right)\left(1-zz^{*}\right)dh^{\prime }+2z^{*}h^{\prime }dz-2zh^{\prime }dz^{*}\right] \end{array}\right.$$

(A25) $$dx_{1}dx_{2}dx_{0}=\frac{-i{h^{\prime }}^{2}\left(1-{\left|z\right|}^{2}\right)}{{\left(1-{\left|z\right|}^{2}\right)}^{6}}\det\left(\begin{array}{ccc} z-z^{*} & 1-{\left|z\right|}^{2} & -\left(1-z^{2}\right)\\ z+z^{*} & 1+z^{*2} & 1+z^{2}\\ 1+{\left|z\right|}^{2} & 2z^{*} & 2z \end{array}\right)$$

(A26) $$dx_{1}dx_{2}dx_{0}=\frac{-2i{h^{\prime }}^{2}}{\left(1-{\left|z\right|}^{2}\right)}dh^{\prime }{\left|dz\right|}^{2}=\frac{2}{\pi }h^{\prime }dh^{\prime }\omega_{h}$$

This the measure defined on open set of $g^{*}$ given by $l_{1}^{2}+l_{2}^{2}-l_{0}^{2}<0$.

## Appendix B. Bargman Parameterization of SU(1,1)

SU(1,1) is isomorphic to SL(2,R)=Sp(2,R) through the complex unitary matrix W:

(A27) $$SL\left(2,R\right)=\left\{g=\left(\begin{array}{cc} a & b\\ c & d \end{array}\right)/\det g=ad-bc=1\right\}$$

(A28) $$Sp\left(2,R\right)=\left\{g=\left(\begin{array}{cc} a & b\\ c & d \end{array}\right)/gJg^{T}=J,J=\left(\begin{array}{cc} 0 & +1\\ -1 & 0 \end{array}\right)\right\}$$

(A29) $$W=\frac{1}{\sqrt{2}}\left(\begin{array}{cc} \omega^{-1} & \omega^{-1}\\ -\omega & \omega \end{array}\right)={\left(W^{+}\right)}^{-1}\;\mathrm{with}\;\omega =e^{i\pi /4}=\frac{1}{\sqrt{2}}\left(1+i\right)$$

If we observe that $W^{-1}JW=-iM$, the isomorphism is given explicitely by:

(A30) (abcd)=g(u)=WuW−1=(Re(α+β)−Im(α−β)Im(α+β)Re(α−β))

(A31) $$\left(\begin{array}{cc} \alpha & \beta \\ \beta^{*} & \alpha^{*} \end{array}\right)=u(g)=W^{-1}gW=\frac{1}{2}\left(\begin{array}{cc} \left(a+d\right)-i\left(b-c\right) & \left(a-d\right)+i\left(b+c\right)\\ \left(a-d\right)-i\left(b+c\right) & \left(a+d\right)+i\left(b-c\right) \end{array}\right)$$

We can also make also a link with SO(2,1) of “1 + 2” pseudo-orthogonal matrices:

(A32) $$SO(2,1)=\left\{\Gamma \in GL\left(3,3\right)/\det\left(\Gamma \right)=1,\Gamma K\Gamma^{T}=\Gamma ,K=\left(\begin{array}{ccc} +1 & 0 & 0\\ 0 & -1 & 0\\ 0 & 0 & -1 \end{array}\right)\right\}$$

(A33) $$\Gamma (g)=\left(\begin{array}{ccc} \frac{1}{2}\left(a^{2}+b^{2}+c^{2}+d^{2}\right) & \frac{1}{2}\left(a^{2}-b^{2}+c^{2}-d^{2}\right) & -cd-ab\\ \frac{1}{2}\left(a^{2}+b^{2}-c^{2}-d^{2}\right) & \frac{1}{2}\left(a^{2}-b^{2}-c^{2}+d^{2}\right) & cd-ab\\ -bd-ac & bd-ac & ad+bc \end{array}\right)$$

with $\Gamma \left(g_{1}\right)\Gamma \left(g_{2}\right)=\Gamma \left(g_{1}g_{2}\right),\Gamma (I)=I,\Gamma \left(g^{-1}\right)=\Gamma {\left(g\right)}^{-1}$

The SO(2,1) matrix corresponds to any SU(1,1):

(A34) Γ(u)=(|α|2+|β|22Reαβ*2Imαβ*2ReαβRe(α2+β2)Im(α2−β2)−2Imαβ−Im(α2+β2)Re(α2−β2))

and $\alpha =\pm \sqrt{\frac{1}{2}\left(\Gamma_{11}+\Gamma_{12}\right)+i\left(\Gamma_{12}-\Gamma_{21}\right)},\beta =\frac{1}{2\alpha }\left(\Gamma_{10}-i\Gamma_{20}\right)$

The properties of connectivity of Sp(2,R) is described by its isomorphy with SU(1,1). Using unimodular condition:

$${\left|\alpha \right|}^{2}-{\left|\beta \right|}^{2}=1\Rightarrow \alpha_{R}^{2}+\alpha_{I}^{2}-\beta_{R}^{2}=1+\beta_{I}^{2}\geq 1\;\mathrm{with}\;\alpha =\alpha_{R}+i\alpha_{I}\;\mathrm{and}\;\beta =\beta_{R}+i\beta_{I}$$

If $\beta_{I}$ is fixed, $\left(\alpha_{R},\alpha_{I},\beta_{R}\right)$ are constrained to define a one-sheeted revolution hyperboloid, with its circular waist in the α plane.

To SU(1,1), we can associate the simply-connected universal covering group, using the maximal compact subgroup U(1) and corresponding to the Iwasawa decomposition (factorization of a noncompact semisimple group into its maximal compact subgroup times a solvable subgroup).

(A35) $$\left(\begin{array}{cc} \alpha & \beta \\ \beta^{*} & \alpha^{*} \end{array}\right)=\left(\begin{array}{cc} e^{i\omega } & 0\\ 0 & e^{i\omega } \end{array}\right)\left(\begin{array}{cc} \lambda & \mu \\ \mu^{*} & \lambda \end{array}\right)\;\mathrm{with}\;\left\{ \begin{array}{l} \omega =\arg\alpha =\frac{1}{2}i\ln\left(\alpha^{*}\alpha^{-1}\right)\\ \lambda =\left|\alpha \right|>0\\ \mu =e^{-i\omega }\beta =\sqrt{\frac{\alpha^{*}}{\alpha }}\beta \end{array}\right.$$

(A36) $$\beta =e^{i\omega }\mu ,{\left|\alpha \right|}^{2}-{\left|\beta \right|}^{2}=\lambda^{2}-{\left|\mu \right|}^{2}=1\;\mathrm{so}\;\left|\mu \right|<\lambda$$

Bargmann has generalized this parameterization for Sp(2N,R), more convenient but difficult to generalize to N dimensions. For SU(1,1):, Bargmann has used (ω,γ):

(A37) $$\gamma =\frac{\mu }{\lambda }=\frac{\beta }{\alpha }\;(\left|\gamma \right|<1)\;,\;\lambda =\frac{1}{\sqrt{1-{\left|\gamma \right|}^{2}}},\mu =\frac{\gamma }{\sqrt{1-{\left|\gamma \right|}^{2}}}$$

For SL(2,R)=Sp(2,R), the Bargman, parameterization is given by this decomposition of a non-singular matrix into the product of an orthogonal and a positive definite symmetric matrix:

(A38) (abcd)=(cosω−sinωsinωcosω)(λ+ReμImμImμλ−Reμ)

Conversely: $\omega =\arg\left[\left(a+d\right)-i\left(b-c\right)\right],\mu =e^{-i\omega }\left[\left(a-d\right)+i\left(b+c\right)\right]$

ω is counted modulo 2π, ω≡ω(mod2π).

SU(1,1) and SL(2,R)=Sp(2,R) are described when ω is counted modulo 2π, ω≡ω(mod2π).

Valentine Bargmann has proposed the covering of the general symplectic group Sp(2N,R):

(A39) $$Sp\left(2N,R\right)=\left\{g=\left(\begin{array}{cc} A & B\\ C & D \end{array}\right)/gJ_{2N}g^{T}=J_{2N},J_{2N}^{T}=-J_{2N},J_{2N}=\left(\begin{array}{cc} 0 & I_{N}\\ -I_{N} & 0 \end{array}\right)\right\}$$

with relations:

(A40) $$AB^{T}=BA^{T},AC^{T}=CA^{T},BD^{T}=DB^{T},CD^{T}=DC^{T},AD^{T}-BC^{T}=I_{N}$$

(A41) $$g\in Sp\left(2N,R\right)\Rightarrow g^{-1}=M_{2N}g^{T}M_{2N}=\left(\begin{array}{cc} D^{T} & -B^{T}\\ -C^{T} & A^{T} \end{array}\right)$$

Bargmann has observed that although Sp(2N,R) is not isomorphic to any pseudo-unitary group, its inclusion in U(N,N) will display the connectivity properties through its unitary U(N) maximal compact subgroup, generalizing the role of U(1)=SO(2) in Sp(2,R).

$W_{N}=W⊗I_{N}$ a 2N×2N matrix where $W=W_{1}=\frac{1}{\sqrt{2}}\left(\begin{array}{cc} \omega_{\pi /4}^{-1} & \omega_{\pi /4}^{-1}\\ -\omega_{\pi /4} & \omega_{\pi /4} \end{array}\right)$ with $\omega =e^{i\pi /4}=\frac{1}{\sqrt{2}}\left(1+i\right)$, which gives the N×N block coefficients.

(A42) $$u\left(g\right)=W_{N}^{-1}gW_{N}=\frac{1}{2}\left(\begin{array}{cc} \left[A+D\right]-i\left[B-C\right] & \left[A-D\right]+i\left[B+C\right]\\ \left[A-D\right]-i\left[B+C\right] & \left[A+D\right]+i\left[B-C\right] \end{array}\right)=\left(\begin{array}{cc} \alpha & \beta \\ \beta^{*} & \alpha^{*} \end{array}\right)$$

with

(A43) $$\left\{ \begin{array}{l} \alpha \alpha^{+}-\beta \beta^{+}=I_{N},\alpha^{+}\alpha -\beta^{T}\beta^{*}=I_{N}\\ \alpha \beta^{T}-\beta \alpha^{T}=0,\alpha^{T}\beta^{*}-\beta^{+}\alpha =0 \end{array}\right.$$

and

(A44) $$u^{-1}=M_{2N}u^{+}M_{2N}^{-1}=\left(\begin{array}{cc} \alpha^{+} & -\beta^{T}\\ -\beta^{+} & \alpha^{T} \end{array}\right)$$

The symplecticity property of g becomes:

(A45) $$uM_{2N}u^{+}=M_{2N},M_{2N}=iW_{N}^{-1}J_{2N}W_{N}=\left(\begin{array}{cc} I_{N} & 0\\ 0 & -I_{N} \end{array}\right)$$

(A46) (ABCD)=g(u)=WNuWN−1=(Re(α+β)−Im(α−β)Im(α+β)Re(α−β))

Valentine Bargmann has extended the well-know theorem that any real matrix R may be decomposed into the product of an orthogonal Q and a symmetric positive definite matrix S, uniquely as R=QS. Bargmann has shown that if R∈Sp(2N,R), then R=QS with Q,S∈Sp(2N,R) where Q maps onto unitary matrix and S maps onto Hermitian positive definite matrix:

(A47) $$u\left(Q\right)=\left(\begin{array}{cc} \alpha & 0\\ 0 & \alpha^{*} \end{array}\right),\alpha \alpha^{+}=I_{N},\alpha \in U(N)\;\mathrm{and}\;u\left(S\right)=\exp\left(\begin{array}{cc} 0 & \xi \\ \xi^{*} & 0 \end{array}\right),\xi =\xi^{T}$$

We can generalize Bargmann parameterization of SU(1,1) to Sp(2N,R):

(A48) $$u\left\{\omega ,\lambda ,\mu \right\}=\left(\begin{array}{cc} e^{i\omega }I_{N} & 0\\ 0 & e^{-i\omega }I_{N} \end{array}\right)\left(\begin{array}{cc} \lambda & \mu \\ \mu^{*} & \lambda^{*} \end{array}\right)⊕,\det\lambda >0$$

Then the Bargmann parameters are:

(A49) $$\omega =\frac{1}{N}\arg\det\alpha ,\lambda =e^{-i\omega }\alpha ,\mu =e^{-i\omega }\beta ,e^{iN\omega }=\frac{\det\alpha }{\left|\det\alpha \right|},\det\lambda =\left|\det\alpha \right|>0$$

The Sp(2N,R) matrices in terms of the Bargmann parameters are:

(A50) g{ω,λ,μ}=(cosωIN−sinωINsinωINcosωIN)(Re(λ+μ)−Im(λ−μ)Im(λ+μ)Re(λ−μ))

V. Bargmann has proposed the covering of the general symplectic group Sp(2N,R):

(A51) $$Sp\left(2N,R\right)=\left\{g=\left(\begin{array}{cc} A & B\\ C & D \end{array}\right)/gJ_{2N}g^{T}=J_{2N},J_{2N}^{T}=-J_{2N},J_{2N}=\left(\begin{array}{cc} 0 & I_{N}\\ -I_{N} & 0 \end{array}\right)\right\}$$

(A52) $$AB^{T}=BA^{T},AC^{T}=CA^{T},BD^{T}=DB^{T},CD^{T}=DC^{T},AD^{T}-BC^{T}=I_{N}$$

Bargmann has observed that although Sp(2N,R) is not isomorphic to any pseudo-unitary group, its inclusion in U(N,N) will display the connectivity properties through its unitary U(N) maximal compact subgroup, generalizing the role of U(1)=SO(2) in Sp(2,R):

$W_{N}=W⊗I_{N}$,2N×2N matrix where $W=W_{1}=\frac{1}{\sqrt{2}}\left(\begin{array}{cc} \omega_{\pi /4}^{-1} & \omega_{\pi /4}^{-1}\\ -\omega_{\pi /4} & \omega_{\pi /4} \end{array}\right)$ with $\omega =e^{i\pi /4}=\frac{1}{\sqrt{2}}\left(1+i\right)$.

(A53) $$u\left(g\right)=W_{N}^{-1}gW_{N}=\frac{1}{2}\left(\begin{array}{cc} \left[A+D\right]-i\left[B-C\right] & \left[A-D\right]+i\left[B+C\right]\\ \left[A-D\right]-i\left[B+C\right] & \left[A+D\right]+i\left[B-C\right] \end{array}\right)=\left(\begin{array}{cc} \alpha & \beta \\ \beta^{*} & \alpha^{*} \end{array}\right)$$

with

(A54) $$\alpha \alpha^{+}-\beta \beta^{+}=I_{N},\alpha^{+}\alpha -\beta^{T}\beta^{*}=I_{N}\;\mathrm{and}\;\alpha \beta^{T}-\beta \alpha^{T}=0,\alpha^{T}\beta^{*}-\beta^{+}\alpha =0$$

The symplecticity property of g becomes:

(A55) $$uM_{2N}u^{+}=M_{2N},M_{2N}=iW_{N}^{-1}J_{2N}W_{N}=\left(\begin{array}{cc} I_{N} & 0\\ 0 & -I_{N} \end{array}\right)$$

(A56) (ABCD)=g(u)=WNuWN−1=(Re(α+β)−Im(α−β)Im(α+β)Re(α−β))

## Appendix C. Shirokov Method to build Casimir function

I. V. Shirokov [71,72,73,74,75] has proposed a method for constructing invariants of the coadjoint representation of Lie groups with an arbitrary dimension and structure based on local symplectic coordinates on the coadjoint orbits. They also have made link with construction of the Lie algebra polarization

$B_{\lambda }\left(X,Y\right)$ be the skewsymmetric form on g defined by:

(A57) $$\begin{array}{l} B_{\lambda }\left(X,Y\right)=\langle \lambda ,\left[X,Y\right]\rangle \;,\;X,Y\in g\;,\;\lambda \in g^{*}\\ \mathrm{with}\;B_{\lambda }\left(X,Y\right)=B_{ij}X^{i}Y^{j}\;,\;B_{ij}=C_{ij}^{k}\lambda_{k}\equiv C_{ij}(\lambda )\;,\;i,j,k=\dim g \end{array}$$

(A58) $$\mathrm{Ker}B_{\lambda }=\left\{X\in g/B_{\lambda }\left(X,g\right)=0\right\}\equiv g^{\lambda }\;\mathrm{annihilator}\;\mathrm{of}\;\lambda \in g^{*}$$

The polarization 𝖍≡𝕻(g) of the the covector λ is given by:

(A59) $$B_{\lambda }\left(𝖍,𝖍\right)=0\;,\;\dim 𝖍=\frac{1}{2}\left(\dim g+\dim g^{\lambda }\right)$$

The polarization of a semisimple Lie algebra is the construction of polarization of the reductive Lie algebra g, where g=C⊕B, where C is the center in g, and B is a semisimple subalgebra, given by:

(A60) 𝖍≡𝕻(C)⊕𝕻(B)=C⊕𝕻(B)

Let g be an arbitrary Lie algebra, R its radical and $R^{⊥}=\left\{X\in g/B_{\lambda }\left(X,R\right)=0\right\}$ the orthogonal complement of R in g with respect to the form $B_{\lambda }\left(X,Y\right)$, then the polarization is given by:

(A61) $$𝕻(g)\equiv 𝕻(R)⊕𝕻(R^{⊥})=ℜ(R)⊕𝕻(R^{⊥})$$

Oleg L. Kurnyavko and Igor V. Shirokov idea of the method of constructing coadjoint invariants is to construct the canonical transition to the Darboux coordinates on the orbits of the dual Lie algebra $g^{*}$ of maximal dimension dual to the Lie algebra g of the Lie group G.

If we consider a Lie group G, its Lie algebra g and its dua Lie algebra $g^{*}$, and let each element $f\in g^{*}$ corresponds to the coordinates $\left(f_{1},f_{2},\ldots ,f_{n}\right)$ where $n=\dim g^{*}$. The action of the group G on $g^{*}$ splits the dual Lie algebra into orbits $O_{\lambda }$ where $\lambda \in g^{*}$ is some element of the given orbit. As with Souriau Theorem, these orbits is a symplectic manifold, with Darboux theorem, there is a special coordinates (p,q) on them such that the corresponding symplectic form can be written:

(A62) $$\omega_{\lambda }=dp_{i}\wedge dq^{i}\;,\;i=1,\ldots ,\frac{1}{2}\dim O_{\lambda }$$

The transition to canonical coordinates is determined by the functions:

(A63) $$f_{j}=f_{j}\left(q,p,\lambda \right)\;,\;j=1,2,\ldots ,\dim g^{*}\;,\;\mathrm{with}\;f_{j}\left(0,0,\lambda \right)=\lambda_{j}$$

Eliminating the variables q and p in these transition functions, we deduce the equation of the orbit:

(A64) $$\psi_{k}(f)=\omega_{k}\;,\;k=1,\ldots ,n-\dim O_{\lambda }\;$$

We obtain $n-\dim O_{\lambda }$ functions such that:

(A65) $$\psi_{k}\left(Ad_{g}^{*}f\right)=\psi_{k}(f)$$

Each nondegenerate orbit is determined by the set of numbers $\omega =\left\{\omega_{1},\omega_{2},\ldots ,\omega_{indg}\right\}$, that is fixed by the choice of the covector λ such that rank $C_{ij}\left(\lambda \right)$ is maximal, and then:

(A66) $$\omega_{k}=\psi_{k}(\lambda_{1})=\psi_{k}(\lambda_{2})\;,\;\mathrm{if}\;\lambda_{1},\lambda_{2}\in O_{\lambda }$$

We can then consider λ(j) orbits $O_{\lambda }$ with $j=\left(j_{1},j_{2},\ldots ,j_{indg}\right)$, with the constraint:

(A67) $$\psi_{k}(\lambda (j))=\omega_{k}(j)\;,\;\det\frac{\partial \omega_{k}}{\partial j}\neq 0\;$$

If we set $\lambda_{i}(j)=a_{i}^{l}j_{l}$ and $X_{k}^{i}$ the components of the vectors that form the basis of the annihilator of the covector λ(j) such that:

(A68) $$\det\frac{\partial \omega_{k}(j)}{\partial j_{k}}=\det\frac{\partial \psi_{k}}{\partial f_{i}}{\frac{\partial f_{i}(j)}{\partial j_{k}}|}_{f=\lambda (j)}=\det X_{k}^{i}\frac{\partial f_{i}(j)}{\partial j_{k}}=\det X_{k}^{i}(\lambda (j))a_{i}^{k}\neq 0$$

Finally, we obtain the parametrization, considering with Darboux coordiantes:

(A69) $$f_{i}=f_{i}\left(q,p,j\right)\;,\;i=1,\ldots ,indg^{*}$$

By eliminating the Darboux coordinate (q,p) in previous equation, we have new relations:

(A70) $$\varphi_{k}\left(f,j\right)=0\;\mathrm{and}\;j_{k}=j_{k}(f)\;,\;k=1,\ldots ,indg$$

These relations provide invariants of the coadjoint representation of the Lie group G.

Igor V. Shirokov has proved that the desired linear transition to the Darboux coordinates on a nondegenerate orbit $O_{\lambda }$ is defined by

(A71) $$f_{i}(p,q)=\xi_{i}^{a}(q)p_{a}+\xi_{i}^{A}(0,q)\lambda_{A}\;\mathrm{where}\;g=\left\{l_{A},l_{a}\right\}\;\mathrm{with}\;l_{A}\;\mathrm{polarization}\;\mathrm{basis}\;\mathrm{of}\;g$$
